# Design, Synthesis, and Evaluation of Naphthyl Pyrazino‐Pyrido‐Pyrimidinones Targeting the Phosphoinositide 3‐Kinase/Alpha‐Serine/Protein Kinase B/Mammalian Target of Rapamycin Pathway

**DOI:** 10.1002/open.202500615

**Published:** 2026-02-12

**Authors:** Marcelo F. Marchiori, Gabriel da Silva, Daniel F. Kawano, Andréia M. Leopoldino, Enrique Madruga, Ana Martinez, Ivone Carvalho

**Affiliations:** ^1^ School of Pharmaceutical Sciences of Ribeirão Preto ‐ University of São Paulo Ribeirão Preto Brazil; ^2^ Faculty of Pharmaceutical Sciences State University of Campinas Campinas Brazil; ^3^ Centro de Investigaciones Biológicas (CIB) del Consejo Superior de Investigaciones Científicas (CSIC) Madrid Spain; ^4^ Centro de Investigación Biomédica en Red en Enfermedades Neurodegenerativas (CIBERNED) Instituto de Salud Carlos III Madrid Spain

**Keywords:** antitumoral evaluation, cancer, molecular docking, phosphoinositide 3‐kinase/alpha‐serine/protein kinase B/mammalian target of rapamycin (PI3K/AKT/mTOR) pathway, pyrazino‐pyrido[2,3‐d]pyrimidine‐5,7‐dione

## Abstract

Cancer remains a leading global cause of death and a major public health concern, with rising incidence and mortality rates. Current treatments are often limited by tumor complexity and heterogeneity, emphasizing the need for novel, targeted, and personalized therapies. Aberrant activation of the phosphoinositide 3‐kinase/alpha‐serine/protein kinase B/mammalian target of rapamycin (PI3K/AKT/mTOR) pathway plays a key role in cancer development, making it an attractive therapeutic target. In this study, we performed in silico and in vitro analyses to assess the antitumor potential of two pyrazino‐pyrido[2,3‐d]pyrimidine‐5,7‐dione Series (A and B) across various cancer cell lines, focusing on possible PI3K/AKT/mTOR inhibition. Guided by these results, we designed a new Series (C) with a fixed C‐9 naphthyl group and variable C‐6 substitutions. The compounds were synthesized via an optimized one‐pot process followed by intramolecular cyclization. Molecular docking and biological assays revealed notable antitumor activity for Series C, particularly for compounds 3 and 4, in BT20, HGC, and CAL‐27 cell lines, while showing selectivity over normal fibroblasts (GNP5). These compounds also affected cell cycle progression and phosphorylation of key proteins involved in autophagy and survival (ULK1, LC3, p‐AKT, p‐STAT3). Overall, this study introduces a promising new scaffold with potent, selective antitumor properties.

## Graphical abstract

Rationally designed naphthyl pyrazino‐pyrido‐pyrimidinones **3** and **4** inhibited 85%–94% of cancer cell growth. Compound **4** exhibited IC_50_ = 7.8–12.8 µM with tumor selectivity. Both induced G1 arrest and modulated ULK1, STAT3, alpha‐serine/protein kinase B (AKT), and autophagy via phosphoinositide 3‐kinase/mammalian target of rapamycin (PI3K/mTOR) inhibition, supported by docking studies, confirming their promise as potent antitumor candidates.

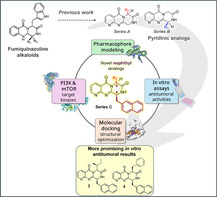

## 1 Introduction

Cancer, characterized by uncontrolled cellular proliferation and potential metastasis, remains a leading cause of morbidity and mortality worldwide. Such dysfunction has taken on alarming proportions in modern society and has become a public health problem, with the most common types of cancer worldwide being female breast cancer (2.3 million cases), lung cancer (2.2 million), colorectal cancer (1.9 million), prostate cancer (1.4 million), and nonmelanoma skin cancer (1.2 million) [1]. The risk of developing cancer rises significantly with age, with incidence rates doubling after the age of 50 and then again after the age of 60. As the global population ages, cancer cases rise accordingly. This association is linked to factors such as cellular senescence, chronic inflammation, DNA damage, mitochondrial dysfunction, and epigenetic modifications [2].

**FIGURE 1:**
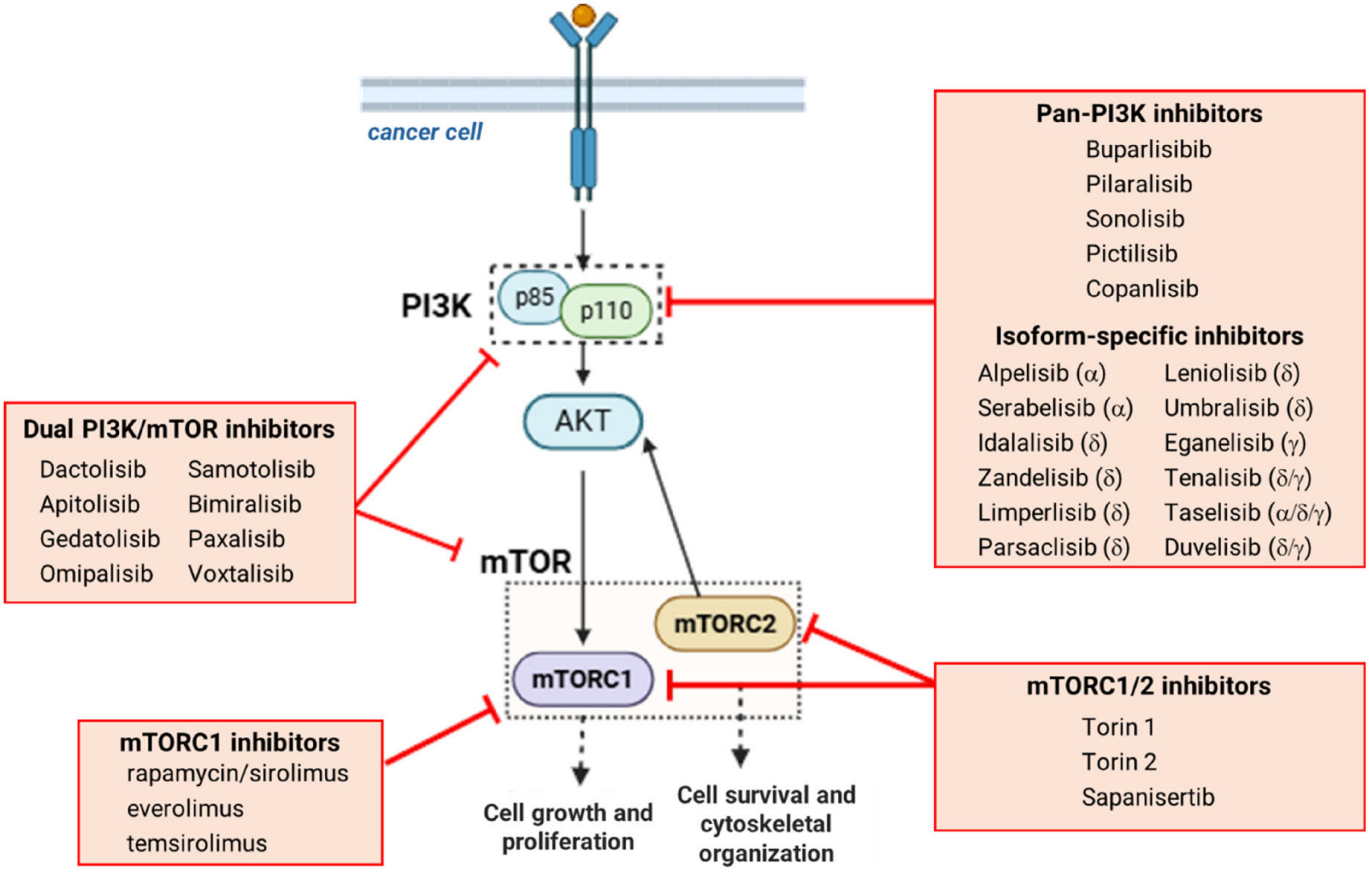
Simplified overview of PI3K/AKT/mTOR inhibitors with some examples of already described compounds in the literature and/or in use in therapy, focusing on PI3K inhibitors, mTOR inhibitors, mTORC1 complex inhibitors, or dual PI3K/mTOR inhibitors (Adapted from MISHRA et al., 2021, under the terms of the Creative Commons Attribution License (CC BY 4.0).).

The phosphoinositide 3‐kinase (PI3K)/alpha‐serine/protein kinase B (AKT)/mammalian target of rapamycin (mTOR) pathway, or PI3K/AKT/mTOR, is a crucial intracellular signaling cascade that regulates metabolism, autophagy, apoptosis, cell growth, survival, transcription, and protein synthesis (Figure 1). Given its role in cell cycle control, it is linked to aging‐related diseases such as cancer, type 2 diabetes, cardiovascular diseases, and neurodegenerative disorders [3]. Amongst the three main classes of PI3K (I, II, and III) based on substrate specificities and structural characteristics, class I PI3Ks are of significant therapeutic interest due to their crucial role in the development of human cancers. These enzymes are heterodimers, consisting of a regulatory subunit (p85) and a catalytic subunit (p110) with four isoforms (*α*, *β*, δ, and *γ*). All currently approved PI3K inhibitors target one or more p110 isoforms of class I PI3Ks and function as ATP‐competitive inhibitors [4, 5]. Furthermore, the catalytic subunit of the mTOR signaling pathway belongs to the serine/threonine kinase family related to phosphoinositide 3‐kinase (PIKK) and is present in two multiprotein complexes, mTORC1 and mTORC2. This subunit is a key target in mTOR inhibition strategies, with its affinity for the classic inhibitor rapamycin determining whether it is sensitive to rapamycin inhibition (mTORC1) or resistant to it (mTORC2) [6].

**FIGURE 2:**
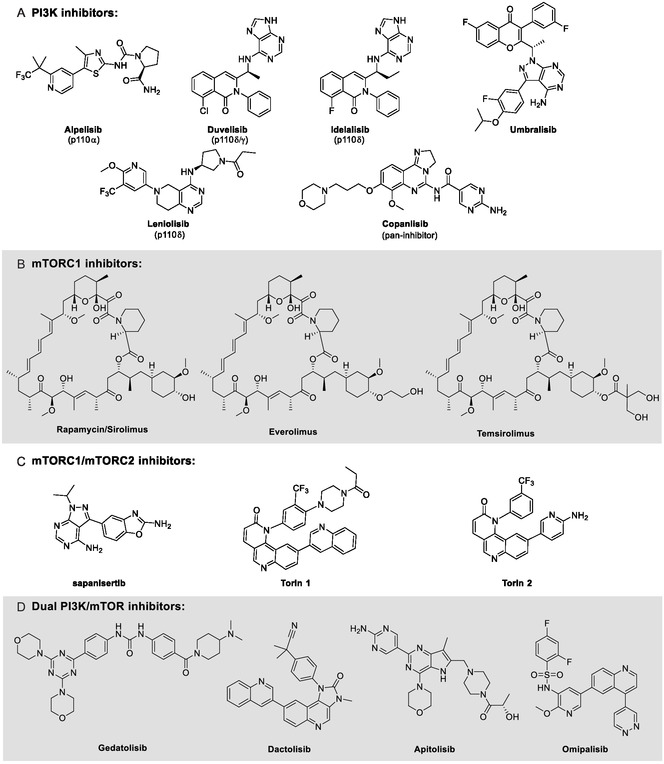
Chemical structures of some inhibitors targeting the PI3K/AKT/mTOR pathway. (A) Pan and isoform‐specific PI3K inhibitors. (B) mTORC1 inhibitors. (C) Inhibitors of both mTORC1 and mTORC2 complexes. (D) Dual PI3K/mTOR inhibitors.

The PI3K/AKT/mTOR signaling pathway has been described as one of the most commonly disrupted pathways in cancer, making it an attractive candidate for therapeutic intervention [7]. In response to this, several targeted inhibitors have been developed and FDA‐approved in the treatment of cancer patients with abnormal activation of the PI3K/AKT/mTOR pathway. This includes pan and isoform‐specific PI3K inhibitors (alpelisib, duvelisib, copanlisib, idelalisib, umbralisib, and leniolisib) as well as the first generation of mTOR inhibitors, known as rapalogs, which consist of rapamycin/sirolimus and its derivatives (everolimus and temsirolimus) [8, 9]. These structures are shown in Figure 2A,B, respectively.

However, in some cases, the individual inhibition of PI3K or mTORC1 fails to suppress a negative feedback loop, leading to the phosphorylation and activation of AKT by mTORC2 and other kinases. As a result, PI3K/AKT/mTOR signaling is not fully blocked. These unsuccessful significant single‐agent antitumor efficacy in most types of cancer led to limited clinical success and to the development of the second generation of mTOR inhibitors, aiming both mTORC1 and mTORC2 (torin 1, torin 2, and sapanisertib—Figure 2C) [10, 11], and dual PI3K/mTOR inhibitors, targeting both PI3K and mTOR kinases (gedatolisib, dactolisib, apitolisib, imipalisib, and others—Figure 2D) [5, 12]. Such a multitarget compounds strategy has emerged as a valuable approach in cancer research, aiming to overcome the challenges posed by the complexity and adaptability of the disease. Unlike traditional single‐target therapies, which often face limitations such as resistance and reduced effectiveness, multitarget strategies offer a more comprehensive treatment by simultaneously modulating multiple molecular pathways. By disrupting essential oncogenic processes, including tumor proliferation, angiogenesis, and apoptosis resistance, these compounds enhance therapeutic efficacy, reduce toxicity, and minimize the risk of resistance development [13].

Dual PI3K/mTOR inhibitors exhibit potent activity against all catalytic isoforms (p110) of PI3K and both mTORC1 and mTORC2. By integrating multiple therapeutic effects into a single molecule, these inhibitors can effectively overcome the feedback activation seen with the individual inhibition of PI3K or mTORC1 [14]. Thus, the concurrent inhibition of both PI3K and mTOR enzymes presents a promising strategy for achieving effective therapeutic outcomes and the development of novel inhibitors targeting the PI3K/AKT/mTOR pathway remains a key focus in anticancer research.

**FIGURE 3:**
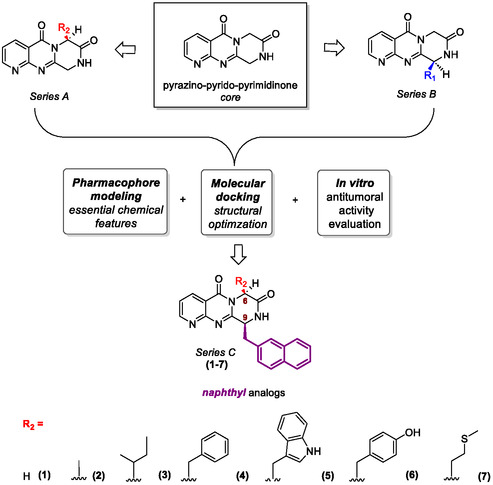
Chemical structures of proposed naphthyl analogs possessing the pyrazino‐pyrido[2,3‐d]pyrimidine‐5,7‐dione scaffold (**1–7**), classified as Series C. The 2‐methylnaphthalene substituent at C‐9 is highlighted in purple and different substituents at C‐6 (R_2_) in red.

In a previous study, we successfully synthesized novel pyrazino‐pyrido‐pyrimidinone derivatives, designed as analogs of the natural alkaloid class of fumiquinazolines (Series A and B, Figure 3) [15]. We analyzed the structural similarity of these compounds with established PI3K/mTOR dual inhibitors. Following pharmacophore modeling, we identified the potential of this tricyclic core for anticancer research. Moreover, *molecular docking* studies were performed with PI3K and mTOR in order to evaluate the theoretical capacity of such structures to bind to these kinases’ active sites. Based on the initial findings, structural modifications were proposed to enhance the affinity of these cores as PI3K and/or mTOR inhibitors. This effort led to the development of a novel naphthyl‐based series (Series C, Figure 3), synthesized using an adapted one‐pot methodology established by our group. Following successful isolation and purification of novel target compounds ‐ achieved with a 30% yield after silica gel column chromatography (CCC) and HPLC purification ‐ these analogs were subjected to in vitro antitumor evaluation across different cancer cell lines, demonstrating promising growth inhibition and potential as anticancer agents. To further elucidate the mechanisms by which the most active compounds exert their effects on BT20, HGC, and CAL‐27 cell lines, we analyzed their impact on cell cycle progression. The results indicated that these compounds induce cell cycle arrest at the G1 phase, a hallmark of mTOR inhibition. Additionally, we investigated the phosphorylation status of key proteins involved in autophagy and survival pathways by Western Blot, including ULK1, LC3, p‐AKT, and p‐STAT3. The phosphorylation patterns varied among the different cell lines, and compounds **3** and **4** consistently demonstrated inhibitory effects on downstream targets regulated by mTORC1 and mTORC2, leading to potential induction of autophagy in the examined cancer cell lines.

## 2 Results and Discussion

### 2.1 Dual PI3K/mTOR Inhibitor Pharmacophore Validation

The design and synthesis of quinazolines with intra—molecular hydrogen bonding scaffolds [16], 4‐methylpteridinones [17], and 4‐methyl pyrido pyrimidinones [18, 19, 20] as dual inhibitors of PI3K/mTOR have been pursued to improve potency and selectivity, using computational modeling, docking studies, and cocrystal structures to optimize binding affinities. In addition, lead optimization through chemical modifications were accomplished to improve drug‐like properties, such as solubility, permeability, and metabolic stability, and overcoming resistance mechanisms associated with PI3K/mTOR inhibition. Given the structural similarity of quinazolines, pteridinones, and pyrido‐pyrimidinones to tricyclic pyrazino‐pyrido‐pyrimidinone derivatives [15], these compounds were analyzed to develop a pharmacophore model. The goal was to identify the essential shared structural and chemical features responsible for key intermolecular interactions that facilitate ligand binding at the target site [21].

**FIGURE 4:**
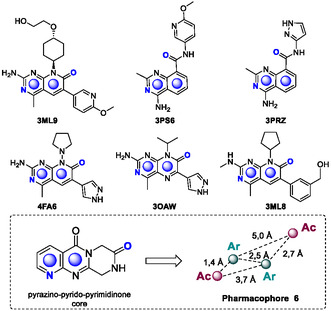
Chemical structures of the six dual PI3K/mTOR inhibitors used to generate shared structural and chemical features, highlighting the common pharmacophoric features related to the pyrazino‐pyrido‐pyrimidinone core. Abbreviations for pharmacophoric groups: Ar = aromatic; Ac = hydrogen bond acceptor. The features that contributed to the pharmacophore of Class 01 are shown in blue.

Therefore, six dual PI3K/mTOR inhibitors (PDB codes: 3PRZ [16], 3PS6 [16], 3OAW [17], 3ML9 [18], 4FA6 [19], and 3ML8 [20] were selected for pharmacophore model generation using the PharmaGist server [22] (Figure 4). This server ranks models based on a scoring system that assesses the extent of overlap among common pharmacophoric features during multiple structure alignment [23]. As the original bioactive conformations of the crystallographic structures are lost during alignment, the ligand positions generated by PharmaGist were compared to their native crystallographic poses by calculating the root–mean‐square deviation (RMSD) values for heavy atoms, excluding hydrogen. Models that, despite high alignment scores and shared pharmacophoric features, did not accurately represent the actual ligand‐target interactions were excluded. The exclusion criterium was based on the thresholds previously described by Caroli et al. [24], where an RMSD ≤ 2 Å would represent poses correctly aligned and, for 2 Å < RMSD ≤ 3 Å, the poses would be only partially correctly aligned. The RMSD values for the generated pharmacophores are summarized in Table 1. Although three models displayed RMSD ≤ 2 Å, the model with the lowest RMSD also displayed the best score and, therefore, was considered the most promising pharmacophore.

**TABLE 1:** Pharmacophore models generated based on the pyrazino‐pyrido‐pyrimidinone core and six quinazolines, 4‐methylpteridinones, and 4‐methyl pyrido pyrimidinones as dual PI3K/mTOR inhibitors.

Model	Score	**Composition** a	**Average RMSD** b
**1**	33.20	Ar, Ar, Ac, Ac, Ac	4.01
**2**	31.50	Ar, Ar, Do, Ac, Ac	11.12
**3**	28.46	Ar, Ar, Do, Ac	3.65
**4**	28.46	Ar, Ar, Do, Ac	3.07
**5**	28.46	Ar, Ar, Ac, Ac	2.35
**6**	**28.46**	**Ar, Ar, Ac, Ac**	**1.17**
**7**	28.46	Ar, Ar, Ac, Ac	4.01
**8**	28.46	Ar, Ar, Ac, Ac	3.25
**9**	23.72	Ar, Ar, Do	1.93
**10**	23.72	Ar, Ar, Ac	1.21

a Abbreviations for pharmacophoric groups: Ar = aromatic; Do = hydrogen bond donor; Ac = hydrogen bond acceptor.

b Average RMSD, values were calculated using the root mean square deviations (RMSD) for heavy atoms (excluding hydrogens) of the crystallographic ligands, expressed in Å.

Within the context of pharmacophore modeling, slight deviations in ligand alignment are anticipated due to conformational flexibility and inherent differences in molecular scaffolds. These variations can be explained by the induced‐fit theory, which suggests that the side chains of amino acid residues in the active site of proteins may undergo slight conformational adjustments to accommodate different ligands, ensuring an optimal interaction profile [25]. Finally, the pharmacophore generated for the pyrazino‐pyrido‐pyrimidinone core was validated, demonstrating key structural features that enable interactions with both mTOR and PI3K, which consist of 2 aromatic interactions and 2 hydrogen bond acceptors groups (Figure 4).

### 2.2 Molecular Docking Studies of Classes A and B

**FIGURE 5:**
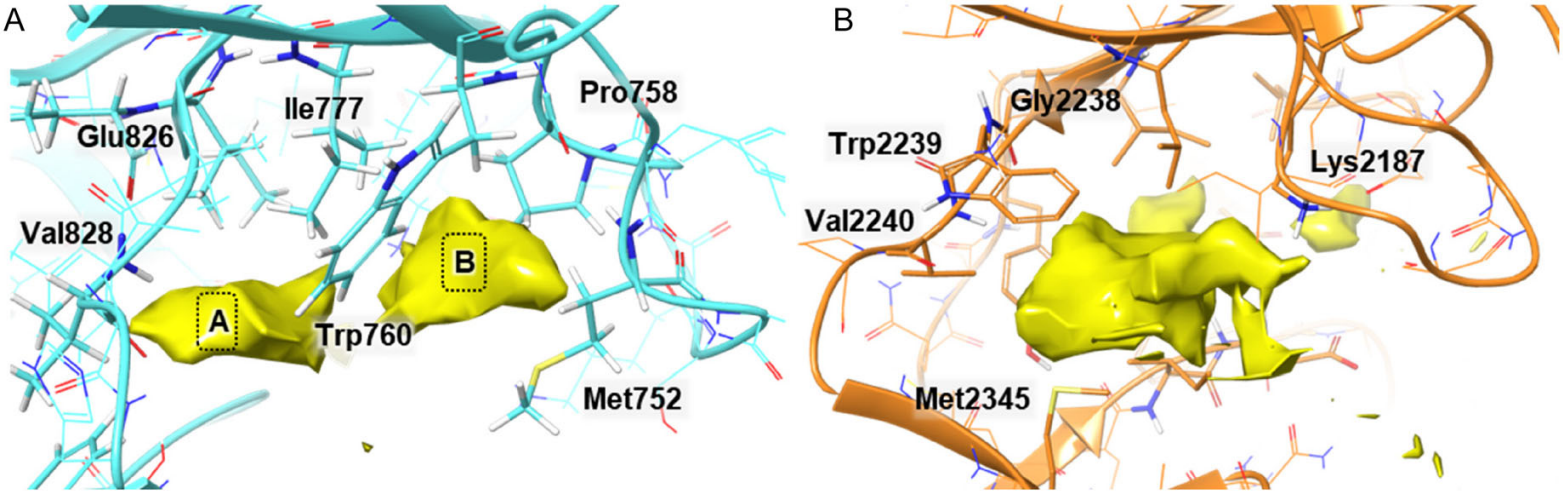
PI3K (PDB:4XE0) and mTOR (PDB:4JT5) active sites. (A) Spatial arrangement of the PI3K active site, highlighting the hydrophobic pocket (yellow), which is split (A and B) by the presence of different hydrophobic amino acids. (B) Spatial arrangement of the key components of the mTOR active site, highlighting the hydrophobic space (yellow).

The evaluation of the binding poses for all proposed ligands was conducted using the kinases PI3K (PDB:4XE0) and mTOR (PDB:4JT5). Similar to other kinases, the PI3K active site is composed of a “hinge” region where the ATP purine ring interacts primarily with residues Glu826 and Val828. Notably, the presence of Trp760, Ile777, Met752, and Pro758 creates a structural division within the PI3K active site, forming two distinct hydrophobic pockets: one adjacent to the “hinge” region (“space A”) and another located behind Trp760 (“space B”) (Figure 5A).

Similar to PI3K, the mTOR kinase active site is composed of another “hinge” region, where key interacting amino acids, Glu2238 and Val2240, are located. However, unlike PI3K, the mTOR active site contains a single well‐defined hydrophobic cavity, where the cocrystallized ligand is found (Figure 5B).

**FIGURE 6:**
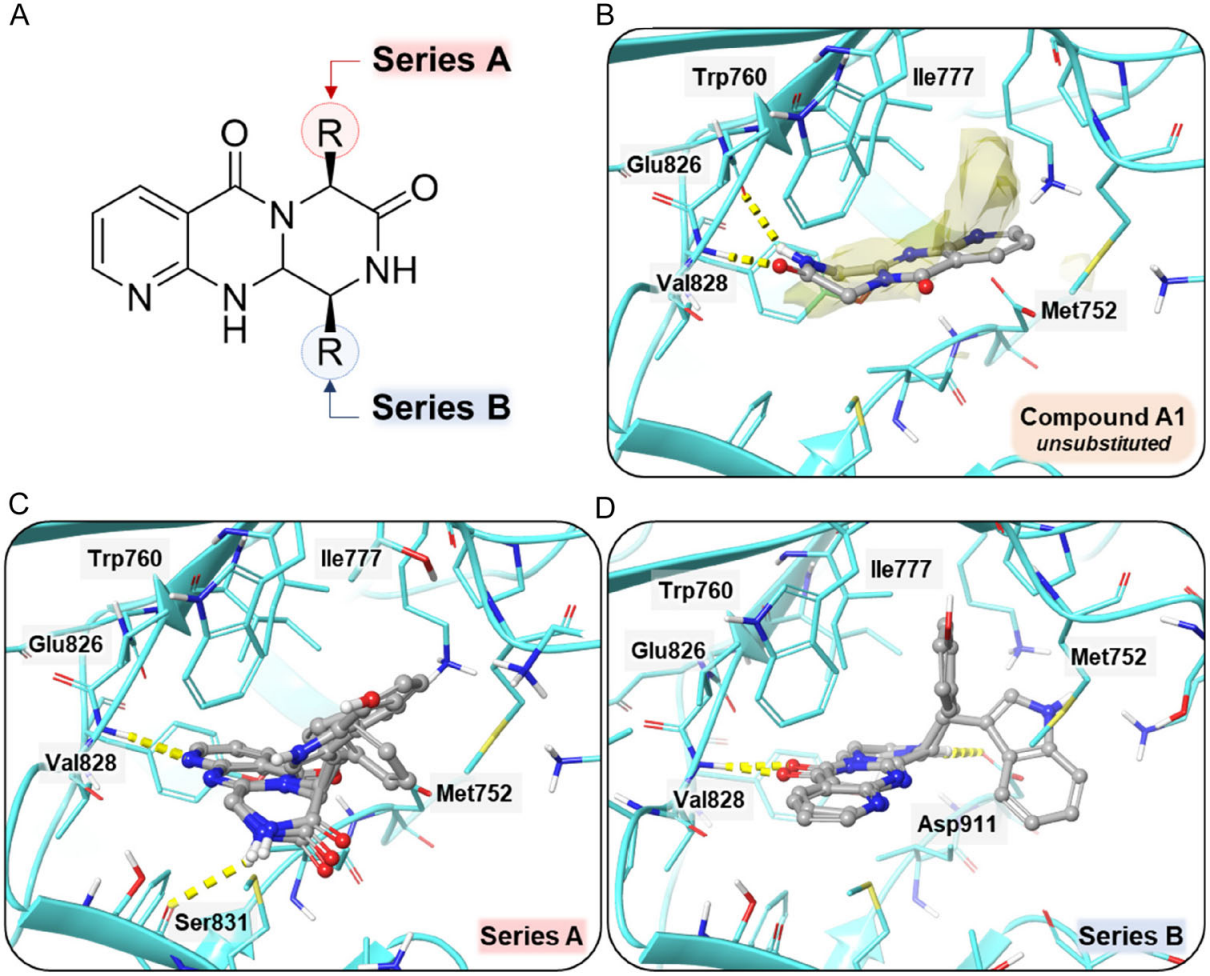
Docking experiments on PI3K (PDB ID: 4XE0) with Series A and B compounds. (A) Overview of the molecules from both series based on their substitution patterns. (B) Docking simulation of the unsubstituted reference compound **A1**. The scaffold occupies only the first hydrophobic pocket (represented as a yellow surface), leaving the second one unoccupied, and forms two hydrogen bonds with Glu 826 and Val 828. (C) Docking simulation of a representative set of compounds from Series A (**A4‐6**). Substituents extend into the second hydrophobic pocket, maintaining two hydrogen bond interactions with Val 828 and Ser 831. (D) Docking simulation of a representative set compound from Series B (**B4‐6**), where substituents tend to occupy the full hydrophobic space. Two hydrogen bonds are observed with Val 828 and Asp 911*.* Yellow dashed line: hydrogen bond.

Both Series, A and B, were preliminary evaluated in silico through docking methodologies to decipher their potential inhibitory activity against PI3K and mTOR, using the unsubstituted compound **A1** as the reference. In case of PI3K, **A1** assumes a classical pose in which the amide group is responsible for the interaction with the hinge region (with the two main amino acids, Glu826 and Val828). Meanwhile, the scaffold occupies the first hydrophobic space (Figure 6B). However, in this configuration, the ligand is unable to occupy both spaces. Furthermore, due to the configuration of the pocket, substituents may not be accommodated optimally within the hydrophobic layer. Increasing the volume and hydrophobicity of the substituents in the Series A results in a complete reordering of the pose, with the substituents orienting to space B. This configuration enables a single interaction with the hinge region (Val 828) via the pyridine ring (Figure 6C). In the context of Series B, substitutes are positioned in close proximity to a voluminous tyrosine (Tyr813) in the original conformation of **A1**. To mitigate the potential for steric clashes, a comprehensive reordering of the conformation is implemented. In this case, the carbonyl group of the pyrimidinone ring establishes an interaction with the hinge region (Val 828), and the substituents are located in space B (Figure 6D). Consequently, there is a slight tendency to increase the docking score value by increasing the size of the substituent (compounds **A3, A6, A7, B4, B6**). Preliminary findings suggest that Series A is a more suitable option than Series B according to the docking scores values (Table 2).

**TABLE 2:** Docking scores values for Series A, B, and C compounds tested with PI3K (PDB:4XE0) and mTOR (PDB:4JT5) kinases.

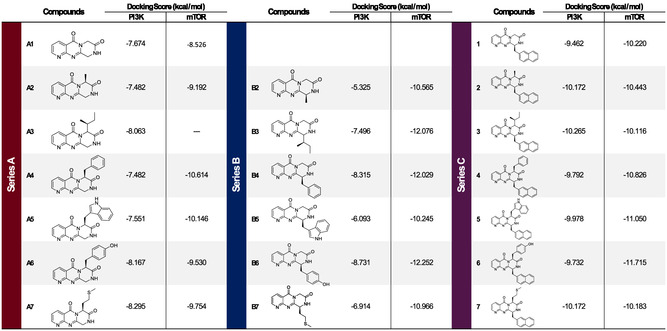

**FIGURE 7:**
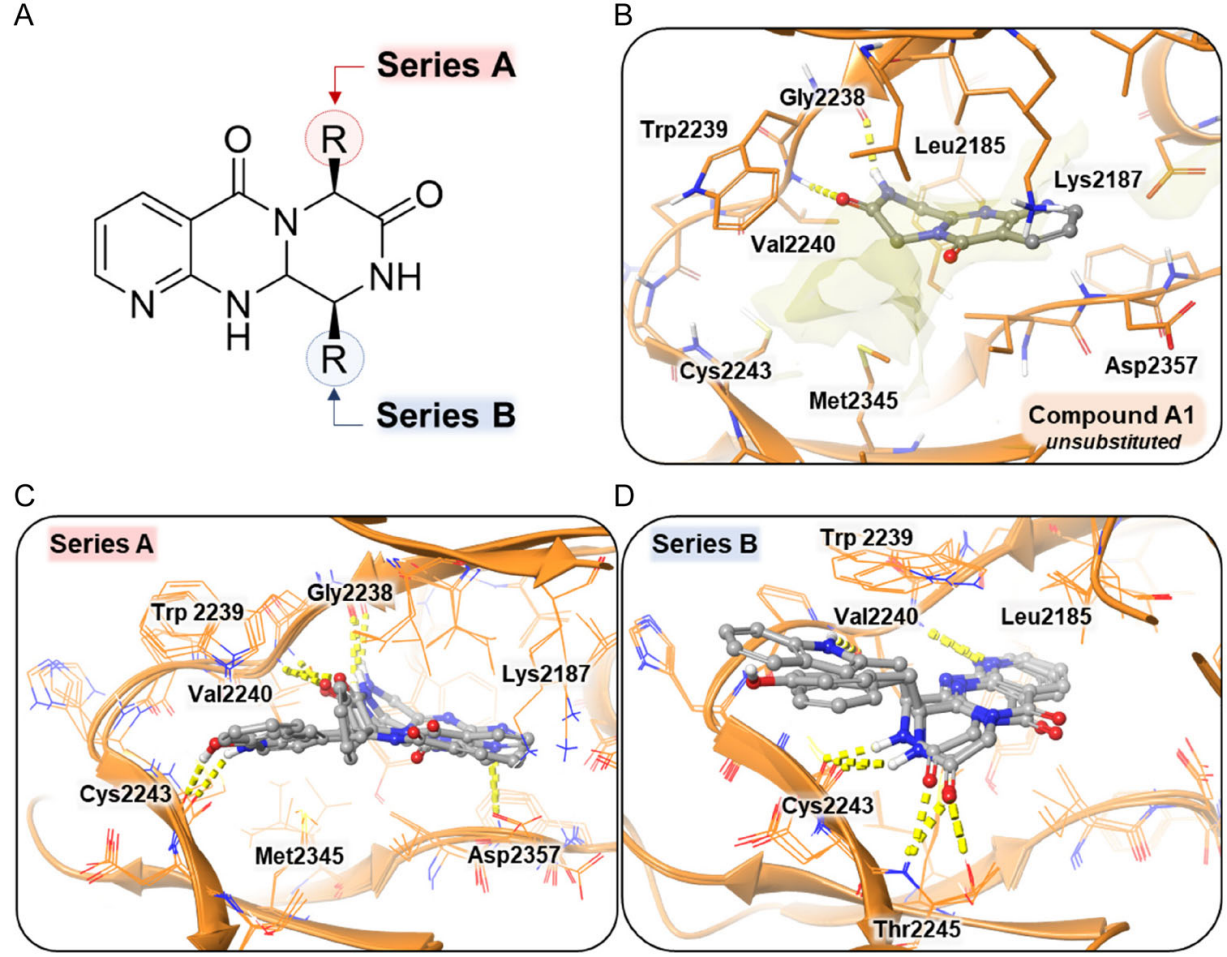
Docking experiments on mTOR (PDB ID: 4JT5) with compounds from Series A and B**.** (A) Structural overview of the molecules from both series, highlighting their substitution patterns. (B) Docking simulation of the reference unsubstituted compound **A1**. The core scaffold partially occupies the hydrophobic pocket (yellow surface), leaving a large area unoccupied, and forms two hydrogen bonds with Gly2238 and Val2240. (C) Docking simulation of representative compounds from Series A (**A4–A6**). All compounds in this series maintain the double hydrogen bond interaction (with Gly2238 and Val2240) and fully occupy the hydrophobic surface through their substituents. (D) Docking simulation of a representative compound from Series B (**B4–B6**). In this case, the binding pose is significantly altered, retaining only one interaction with the hinge region (Val2240), along with two hydrogen bond interactions (Cys2243 and Thr2245). As in Series A, the substituents are well‐positioned to fill the hydrophobic pocket. Yellow dashed lines: hydrogen bonds.

For mTOR, the ligand of reference, **A1**, is able to partially cover the hydrophobic space and interact with the hinge region with the amide group, similar to PI3K (Figure 7B). Classical docking approaches did not demonstrate a consistent pose for both series, which can be attributed to the steric hindrance caused by Trp2239 and Leu2185, impeding the accommodation of bulky substituents. Consequently, the induced fit docking approach was employed, with the active site residues designated as flexible. In the case of Series A, the double interaction with the hinge region is maintained, while the substituents are arranged to occupy the remaining hydrophobic space (except for the compound **A3**, which is unable to obtain this kind of pose) (Figure 7C). In the case of Series B, the necessity to occupy the entire hydrophobic space is also a driving force behind the search for new poses. Consequently, the pyridine ring establishes a hydrogen bond interaction with Val 2240, while the remaining substituents are oriented toward the hydrophobic space (Figure 7D). In all cases, a positive correlation is observed between the size of the substituent and the docking score, particularly in Series B, which demonstrates a slight improvement in performance compared to Series A (Table 2).

### 2.3 Antitumoral Assays of Series A and B

**FIGURE 8:**
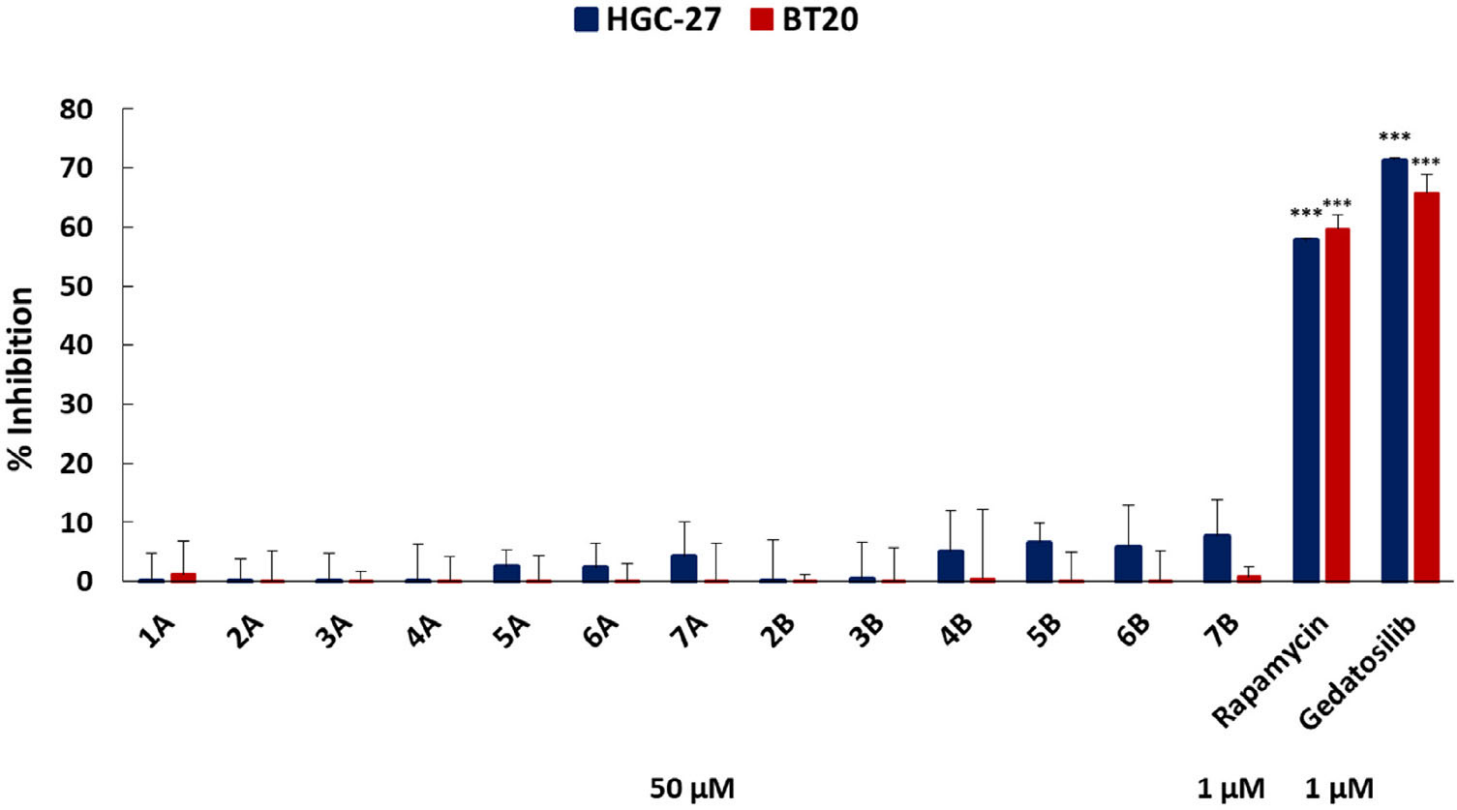
Antitumoral assay demonstrating the percentages of cell inhibition toward tumor cell lines HGC‐27 (blue) and BT20 (red) using Series A and B compounds (50 µM) after 72 h of treatment. Cell viability was determined by the resazurin assay, with rapamycin (1.0 µM) and gedatolisib (1.0 µM) used as positive controls. (***p < 0.001; negative control versus treatment using Student's t‐test).

After the docking studies, the in vitro antitumoral evaluation of both Series A and B was performed. The resazurin assay was used to determine the cell viability of two distinct tumor cell lines originating from gastric cancer (HGC‐27) and breast cancer (BT20). Rapamycin, a selective mTORC1 inhibitor, and gedatolisib, a dual PI3K/mTOR inhibitor, were included as positive controls. As shown in Figure 6, rapamycin at a concentration of 1.0 µM reduced cell viability by approximately 58% in both HGC‐27 and BT‐20 cell lines. On the other hand, the dual PI3K/mTOR inhibitor Gedatolisib, at the same concentration, exhibited greater inhibitory activity, reducing cell viability by 72% in HGC‐27 and 66% in BT‐20 cells. These findings suggest that the dual inhibition of PI3K and mTOR within the PI3K/AKT/mTOR signaling pathway results in greater suppression of cell proliferation and viability compared to selective inhibition of the mTORC1 complex (Figure 8).

At a concentration of 50 µM, none of the tested compounds achieved inhibition levels comparable to those of the positive controls. However, compounds from Series B demonstrated slightly higher antitumoral activity than those from series A. This trend was particularly evident in the HGC‐27 cell line, where compounds **4‐7B** reduced cell viability by 5%, 7%, 6%, and 7%, respectively. These findings suggest a modest but promising potential for Series B compounds, supporting the continued optimization and development of new derivatives.

Collectively, the in silico and in vitro findings revealed a divergent trend regarding the relative potential of Series A and B bearing bulky substituents. Molecular docking studies predicted slightly higher binding affinities for series A with PI3K and Series B with mTOR. Additionally, in vitro antitumor assays indicated a more pronounced biological activity for Series B. Despite this discrepancy, both approaches consistently demonstrated that increasing the size of the substituents correlated with enhanced activity. Guided by these insights, we designed a new series of analogs (Series C), derived from Series B, incorporating a fixed naphthyl group at the C‐9 position to preserve the favorable interactions observed. Furthermore, modifications at the C‐6 position were introduced to increase hydrophilicity and steric volume, while maintaining the naphthyl moiety at C‐9, as illustrated in Figure 3.

### 2.4 Synthesis of Series C

To the best of our knowledge, the synthesis of disubstituted pyrazino[1,2‐a]pyrido[3,2‐d]pyrimidinones bearing a naphthyl moiety has not yet been reported. Moreover, only a single study has described the synthesis of a disubstituted pyrazino‐pyrido‐pyrimidinone scaffold, incorporating an indole group at the C‐6 position and either an isobutyl or sec‐butyl substituent at C‐9 as active antiparasitic compounds against *Plasmodium* and trypanosomatids [26]. Moreover, after developing a new protocol using an adapted method based on the sequential one‐pot coupling of two L‐amino acids [27], we successfully synthesized disubstituted derivatives containing the pyrazino‐pyrido[2,3‐d]pyrimidine‐5,7‐dione core by sequentially coupling two different amino acids under different conditions. Relying on the same approach, we proposed the synthesis of novel naphthyl analogs (**1–7**) starting from 2‐amino nicotinic acid (**8**), N‐Boc‐3‐(2‐naphthyl)‐L‐alanine (**9**), and different L‐amino acids (glycine, valine, isoleucine, phenylalanine, tryptophan, tyrosine, and methionine) [15].

**SCHEME 1:**
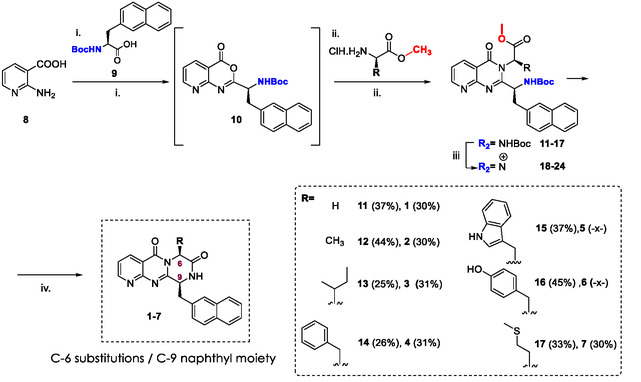
Synthesis of pyrazino‐pyrido[2,3‐d]pyrimidine‐5,7‐diones, **1–6** (Series A). Reaction conditions: (i) P(OPh)_3_, anhydrous pyr, *N*Boc‐aa (R_1_); MW: 100°C, 10 min; (ii) anhydrous pyr., L‐tryptophan methyl ester aa **22**, MW: 150°C, 2.0–2.5 min; (iii) TFA/DCM 1:1 v/v (2.0 mL), RT, 2.0–3.0 h; and (iv) NaHCO_3_ sat. solution until pH 7–8, RT.

Initially, the coupling reaction between precursor **8** and the naphthyl derivative **9** at microwave irradiation at 100°C for 10 min afforded the 4H‐pyrido[2,3‐d [1, 3] oxazin‐4‐one intermediate **10** (not isolated), featuring a naphthyl moiety at the C‐9 position. Subsequently, intermediate **10** was individually treated with a series of methyl ester–protected amino acid derivatives and subjected to a second microwave irradiation at 150°C for 2.0–2.5 min. This one‐pot procedure afforded the pyrido[2,3‐d]pyrimidin‐4(3H)‐one intermediates **11**–**17**, each bearing side chains corresponding to the respective amino acid moieties introduced. The reaction sequence enabled the rapid generation of structural diversity at the C‐6 position (Scheme 1). After CCC purification, the ^1^H NMR spectra of intermediates **11–17** revealed characteristic hydrogen signals, such as the presence of aromatics (H‐2, H‐3, and H‐4) and those corresponding to OMe and *N*Boc protective groups δ 3.74–3.32 (s, 3H) and δ 1.35–1.17 (s, 9H), respectively. In addition, major m/z consistent with the theoretical values of the protonated adducts [M + H^+^] of **11–17** were also detected at high‐resolution electrospray ionization mass spectrometry (HRESI‐MS) positive mode.

It is noteworthy that in our previous investigations, disubstituted analogs exhibited significant restraint to the terminal intramolecular cyclization step required for tricyclic core formation, predominantly due to steric hindrance imposed by the C‐6 and C‐9 amino acid side chains. To address this issue, an additional deprotection step was implemented to remove the *N*‐Boc protecting group [28], followed by neutralization of the resulting ammonium salt, thereby generating the free amine necessary for subsequent transformations Consequently, the intramolecular nucleophilic attack of the free amino group on the ester carbonyl proceeded efficiently, affording the desired pyrazino‐pyrido[2,3‐d]pyrimidine‐5,7‐dione core structure [15].

Efforts to achieve *N*‐Boc deprotection followed by neutralization to facilitate the final cyclization were unsuccessful in two out of the seven intermediates examined. Specifically, the tryptophan‐ and tyrosine‐derived intermediates (**22** and **23**, respectively) failed to furnish the corresponding cyclized products **5** and **6**. This outcome persisted despite adjustments to reaction conditions, including variations in TFA/DCM ratios, reaction time, temperature, and the amount of NaHCO_3_ used for pH neutralization. It was hypothesized that the combined steric bulk of the indole and naphthyl carbonyl substituents may introduce significant spatial hindrance, thereby impeding the efficiency of the final cyclization step. Furthermore, the phenol hydroxyl group (pKa ~ 10) may undergo deprotonation upon NaHCO_3_ addition, increasing its reactivity and polarity, which could promote the formation of undesirable byproducts.

Therefore, after HPLC purifications, five novel naphthyl analogs (**1–4,7**) in 30%–31% yields were afforded. The pyrazino‐pyrido‐pyrimidinone core for all derivatives exhibited characteristic ^1^H NMR signals for the pyridine moiety which were identified as characteristic double‐doublet related to H‐2, H‐4, and H‐3 protons around δ 8.99, 8.72, and 7.52, respectively. In addition, aromatic hydrogens related to naphthyl moiety were observed ranging from δ 7.90–6.99 and H‐6 and H‐9 methine hydrogens between δ 5.58–5.32, and δ.5.01−4.90, respectively. The carbon signals were indirectly identified using 2D NMR techniques and showed C‐2, C‐4, C‐3, C‐6, and C‐9 signals nearby δ 157.0, 137.5, 122.8, 58.8–44.3, and 58.1−53.3, respectively. Furthermore, the structures of the products were confirmed by HRESI‐MS, which displayed mass‐to‐charge ratio (m/z) relations of protonated [M + H^+^] or deprotonated adducts [M − H^+^] . These data are comprehended in the Supplementary Information file.

### 2.5 Antitumoral In Vitro Assays for Series C

**FIGURE 9:**
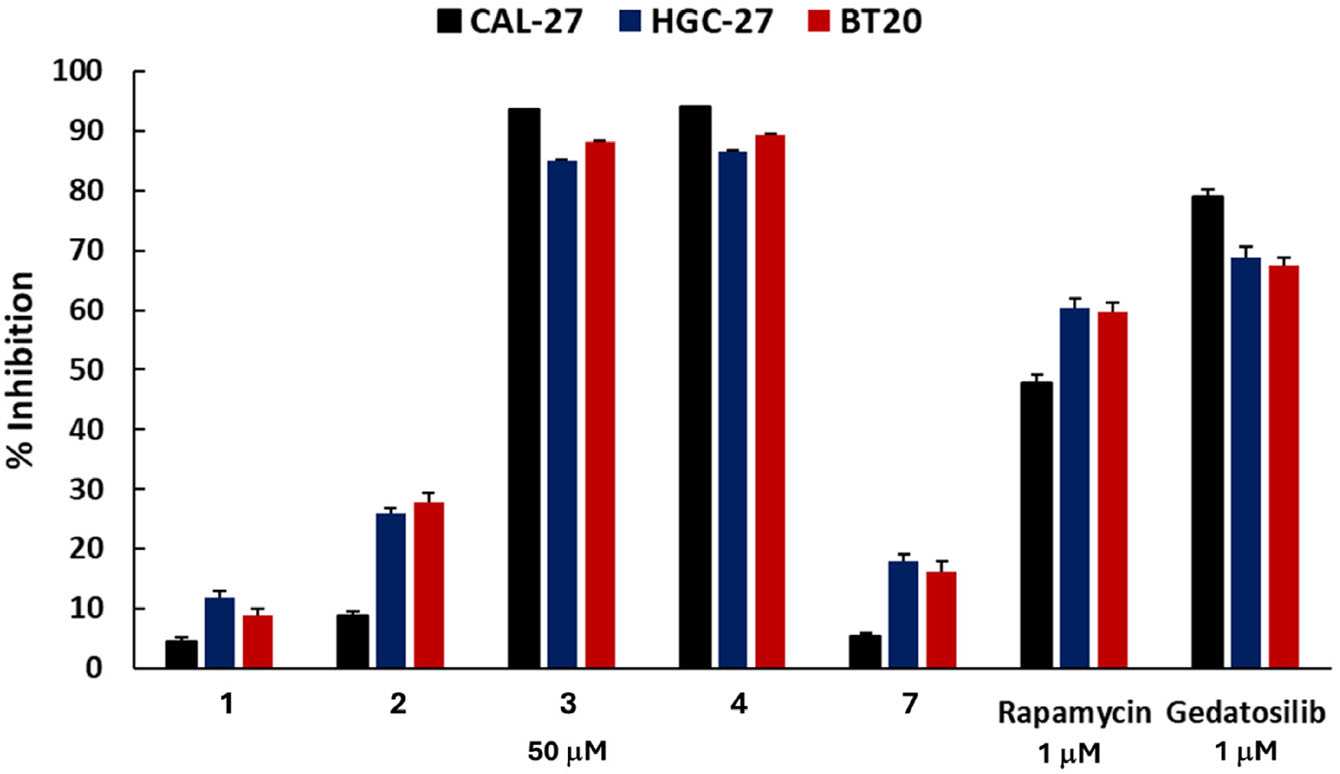
Percentages of cellular inhibition obtained for the tumor cell lines CAL‐27 (black), HGC‐27 (blue), and BT20 (red) incubated with Series C compounds (**1, 2, 3, 4,** and **7**) (50 µM) after 72 h of treatment and with rapamycin and gedatolisib used as positive controls (1 μM). Cell viability was determined by the resazurin assay. (*p < 0.05, **p < 0.01, ***p < 0.001; negative control versus treatment using Student's t‐test).

The incubation protocol followed the same method as previously described for the Series A and B derivatives. As illustrated in Figure 9, the antitumor results for Series C demonstrate that compound **1** (with a methylene group at C‐6 and a naphthyl group at C‐9) exhibited moderately superior activity against HGC‐27 cell line (12%) when compared to the series B compounds at same concentration of 50 μM (Figure 8), further confirming that increasing the volume of the C‐9 substituent enhances activity. Notably, as the volume of the C‐6, bearing alkyl and benzyl substituents, increased while retaining the naphthyl group at C‐9, the inhibition of tumor cells also improved. The most promising results were observed for compounds **3** and **4**, which displayed significantly higher inhibitory activities than any of the Series A or B compounds, achieving inhibition rates of 85% to 94% at 50 µM across all three cell lines (HGC‐27, BT20, and CAL‐27).

Based on these results, compounds **3** and **4** were selected for further biological evaluation, including the determination of their IC_50_ values in these cell lines. Thus, serial dilutions of control rapamycin and gedatolisib were prepared in the ranges of 50–5 µM and 50–2.5 µM, respectively. For the Series C compounds—**3** and **4—**dilutions were prepared in the range of 100–6.25 µM. After conducting a new inhibition test on the CAL‐27, HGC‐27, and BT20 tumor cell lines, the IC_50_ results were compiled in Table 3. It shows that the controls were more potent and selective than the derivatives **3** and **4** across all three tumor cell lines, with gedatolisib demonstrating a better profile than rapamycin, consistent with literature data and the observations in Figures 8 and 9. Additionally, derivative **4** exhibited lower IC_50_ values for the three tumor cell lines compared to **3** and demonstrated greater selectivity when comparing the IC_50_ values of the tumor cell lines with those of the normal oral fibroblast line (GNP5), used as a control. It is worth recalling that while the derivative **3** has a sec‐butyl radical chain at C‐6, compound **4** has a methyl‐benzene radical at this position.

**TABLE 3:** IC_50_ values calculated for the Series C derivatives (compounds 3 and 4) against the normal fibroblast line (GNP5) and tumor cell lines HGC‐27, BT20, and CAL‐27, with rapamycin and gedatolisib used as controls.

Cell Lines	**IC**_**50**_, **µM**			
	Rapamycin	Gedatolisib	3	4
Normal oral fibroblast (GNP5)	>50	>50	24.0 [16.6–34.5]	19.2 [14.6–24.9]
HGC‐27	<5	<2.5	12.5 [9.4–16.3]	7.8 [6.8–8.9]
BT20	<5	<2.5	10.6 [8.1–13.6]	6.9 [5.9–7.9]
CAL‐27	<5	<2.5	14.2 [8.0–23.7]	12.9 [6.3–17.6]

**FIGURE 10:**
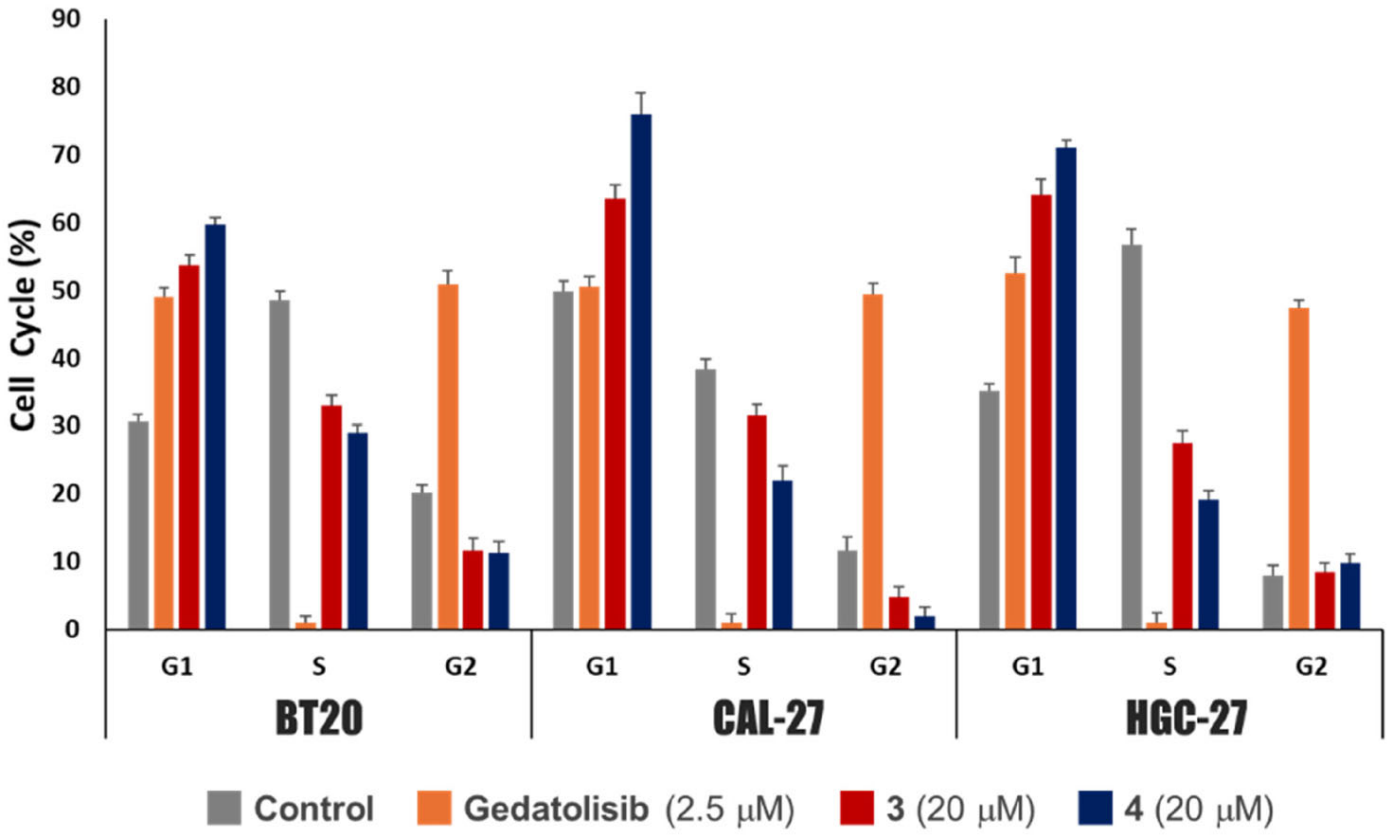
Analysis of the cell cycle phases in the tumor cell lines BT20, CAL‐27, and HGC‐27 after 24 h of treatment with compounds **3** and **4** (20 µM) and with the dual PI3K/mTOR inhibitor gedatolisib (2.5 µM). Flow cytometry analysis evidenced the distribution of subpopulations during cell cycle phases G0/G1, S, and G2/M with strong arrest in G2/M by Gedatolisib and G0/G1 by compounds 3 and 4, accompanied by reduction in S phase. (*p < 0.05, **p < 0.01, ***p < 0.001; negative control versus treatment using Student's t‐test).

Furthermore, an analysis was conducted to evaluate the influence of the derivatives **3** and **4** on the cell cycle. As shown in Figure 10, compounds **3** and **4** at 20 μM induced a significant arrest of the HGC‐27, BT20, and CAL‐27 cell lines in the G1 phase of the cell cycle, suggesting a potential mTOR inhibitory capacity, as mTOR inhibitors are known to cause cell cycle arrest at this phase [29]. This phenomenon occurs because several downstream pathways of mTOR are involved in regulating the cell cycle. For instance, mTOR inhibition can lead to G1 arrest by reducing p‐STAT3 levels, since STAT3 is associated with the activation of various genes involved in cell cycle progression, such as cyclin A, D1, D2, D3, Cdc25A, and WDHD1 [30, 31, 32, 33].

**FIGURE 11:**
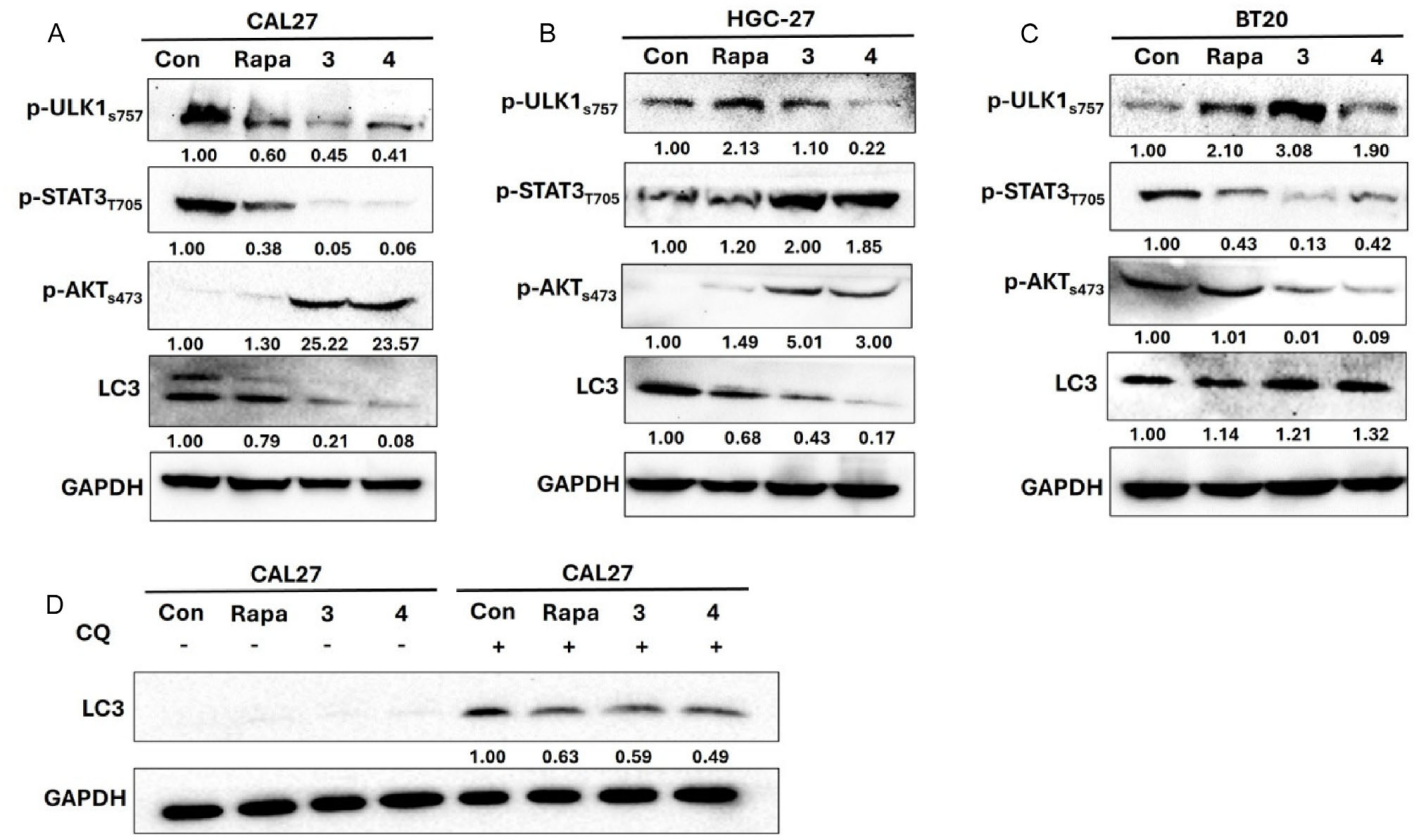
PI3K/AKT/mTOR pathway signaling was affected by compounds **3** and **4** in a cell line‐dependent manner. (A–C) Cells were treated with 10 µM Rapamycin (Rapa), 20 µM compounds **3** and **4** for 6 h, then the cells were collected and lysed to obtain the protein lysate. Subsequently, 40 µg of proteins were subjected to Western Blot analysis. (D) CAL27 cells were treated with 20 µM chloroquine (CQ) for 30 min and, subsequently, with derivatives **3** and **4** (20 µM) for another 6 h. Then, the cells were collected and the proteins were isolated and subjected to Western blot analysis for LC3. The numbers shown below the protein bands in the Western blot correspond to the densitometric analysis. GAPDH is used as a protein loading control in the gel.

**FIGURE 12:**
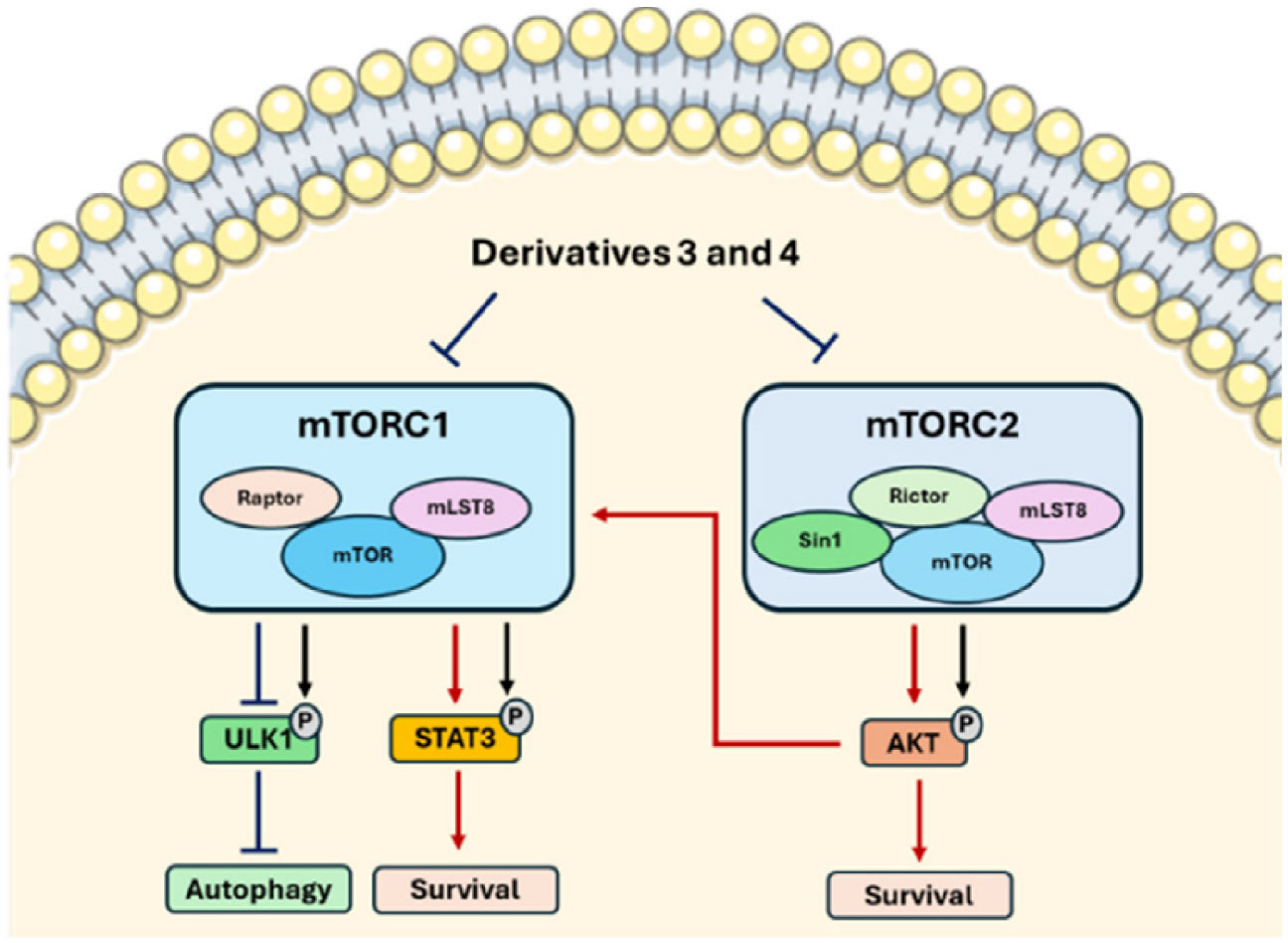
mTORC1/2 inhibition by derivatives **3** and **4** leads to reduced phosphorylation of key downstream targets. This coordinated suppression of phospho‐signaling impacts autophagy and concurrently inhibits STAT3‐ and AKT‐driven cell survival and proliferation.

Finally, the impact of the derivatives **3** and **4** on the levels of proteins involved in the PI3K/AKT/mTOR pathway was assessed in the tumor cell lines BT20, CAL‐27, and HGC‐27 using western blot analysis. The results indicate that compounds **3** and **4** affect signaling pathways in a cell line‐dependent manner. As shown in Figure 11A–C, treatment with derivatives **3** and **4** caused a reduction in ULK1 phosphorylation (Ser757) in CAL‐27 and HGC‐27 cells, indicating inhibition of mTOR1, while BT‐20 had an increase under exposure to compound **3**. The mTORC1 complex is responsible for the phosphorylation of ULK1 in several residues, including S757, which regulates (negatively) the initiation of autophagy under nutrient‐rich conditions [34]. However, in CAL‐27 cells, there was an apparent reduction in LC3 compared to control cells (Figure 11A), even in the presence of chloroquine (Figure 11D), indicating a possible lower autophagic flux in the presence of the derivatives **3** and **4** compared to control [35]. Similarly, HGC‐27 showed a reduction in LC3, while BT‐20 presented an apparent accumulation in LC3 (Figure 11B and C). Interestingly, a significant decrease in p‐AKT (S473) was observed in BT20 cells (Figure 11C), which did not occur in CAL‐27 cells (Figure 11A) and HGC‐27 (Figure 11B). AKT S473 phosphorylation can be mediated by mTORC2 [36], which indicates inhibition of mTORC1 or mTORC2 by compounds **3** and **4**, depending on the cell type. This is reinforced by the fact that the increase in p‐AKT (S473) in CAL27 may have occurred via mTORC1 inhibition [37]. CAL27 and BT20 cell lines present high basal levels of p‐STAT3 (Tyr705), and derivatives **3** and **4** were able to reduce STAT3 phosphorylation in both cell lines, which may be related to their action on mTORC1/2, since both complexes can activate STAT3 via phosphorylation [38, 39]. HGC‐27 showed an increase in pSTAT3 levels. In Figure 12, we summarize the relationship between the mTORC1/2 complexes and the phosphorylation status of the analyzed proteins. Under a specific cellular context, derivatives **3** and **4** may inhibit mTORC1/2 activity, resulting in a reduction of phosphorylation of ULK1, STAT3, and AKT.

These results demonstrate that compounds **3** and **4** inhibit downstream targets regulated by mTORC1/2, thereby disrupting survival signaling pathways and autophagy in a cancer‐ and cell line–specific manner. Such variability is expected, given the distinct genetic backgrounds of the head and neck, gastric, and breast cancer cell lines evaluated.

### 2.6 Molecular Docking of Series C

**FIGURE 13:**
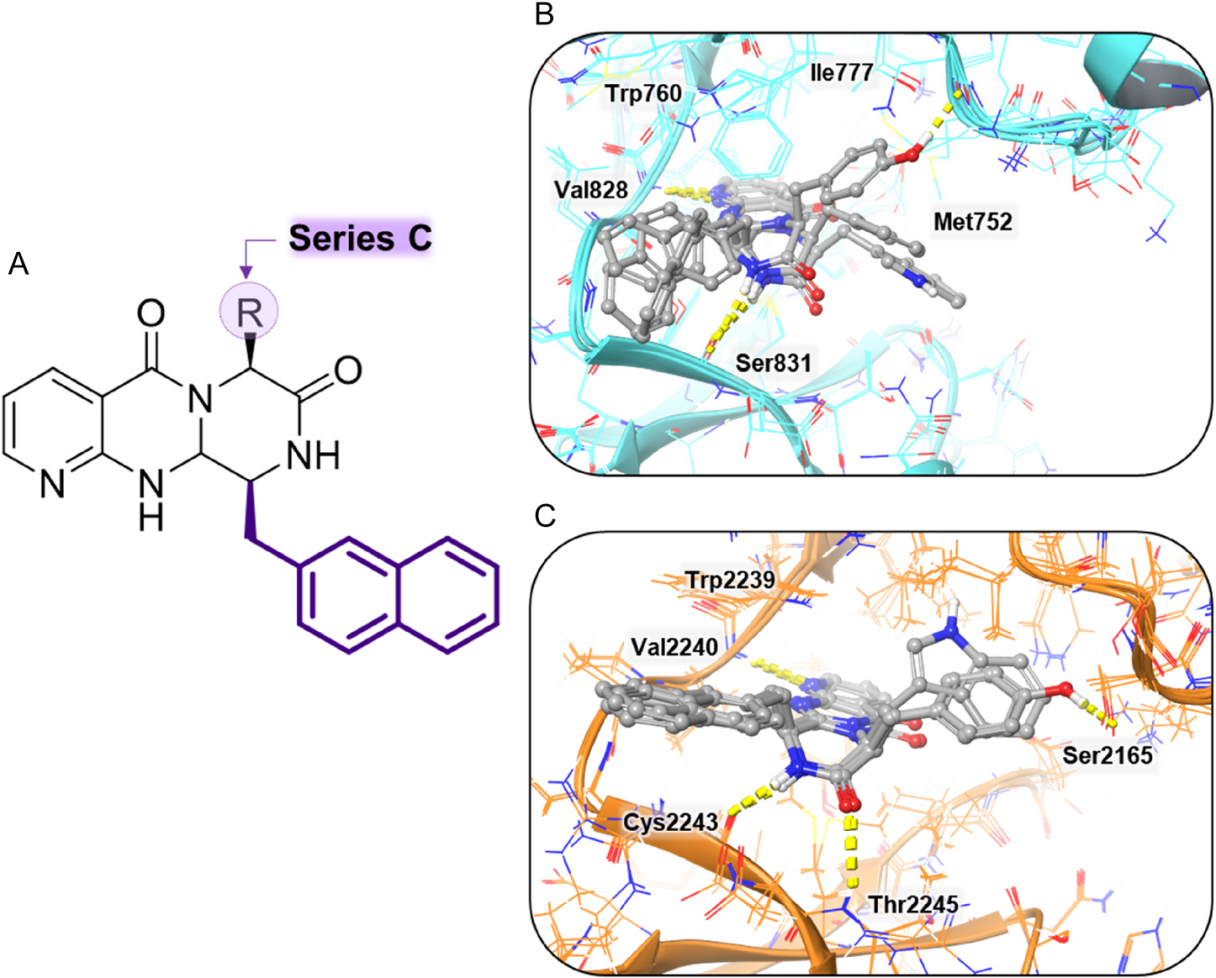
Docking experiments on PI3K (PDB ID: 4XE0) and mTOR (PDB ID: 4JT5) with Series C compounds. (A) Structural overview of the molecules from Series C. (B) Docking simulation of representative compounds from Series C (**C4–C6**) on PI3K. The compounds establish one hydrogen bond with the hinge region (Val828) and a second with Ser831, while occupying both hydrophobic pockets, similar to compounds from Series A. (C) Docking simulation of representative compounds from Series C (**C4–C6**) on mTOR. The molecules interact with the hinge region through a hydrogen bond with Val2240, and form two additional hydrogen bonds with Cys2243 and Thr2245. As observed with Series B, the naphthalene moiety is positioned to fully occupy the hydrophobic pocket.

Due to the large volume of substituents at both positions, no coherent poses were identified when the protein was regarded as rigid. Consequently, the induced‐fit docking methodology was employed for both targets. In the case of PI3K, the compounds interact with the hinge region in a manner analogous to Series A, forming a hydrogen bond through the pyridine ring at position (Val828). The naphthyl ring, along with the tricyclic scaffold, is known to occupy hydrophobic pocket A, while the substituent at position 6 tends to occupy hydrophobic pocket B (Figure 13B). Docking analysis revealed that compounds from Series C exhibited more favorable binding affinities toward PI3K kinase compared to those from Series A and B. Specifically, the average docking scores for Series A and B were −7.67 and −7.21, respectively, while Series C compounds demonstrated a significantly lower average score of −9.98. Even when comparing the best docking score of each class (compound A7 with −8.295 kcal/mol; compound B6 with −8.731 kcal/mol and compound C3 with −10.265 kcal/mol), it is possible to observe a stronger predicted interaction for Series C, as shown in Table 2.

In the case of mTOR, the compounds exhibit a binding tendency analogous to that of Series B, forming a hydrogen bond with Val2240 through the nitrogen of the pyridine ring (Figure 13C). The molecular docking scores of Series C compounds against mTOR ranged from −10.116 to −11.715 kcal/mol, indicating slightly improved binding affinities compared to Series A, which showed an average score of −9.642kcal/mol. However, Series C scores were still less favorable than those of Series B, which ranged from −10.245 to −12.252 kcal/mol (Table 2).

The double interaction binding mode (comparable to Series A for PI3K and analogous to Series B for mTOR), in conjunction with the aggregated docking scores, may provide a rationale for the enhanced efficacy observed with Series C.

## 3 Conclusions

The synthesis, in vitro antitumor evaluation across two distinct cancer cell lines, and molecular docking studies of monosubstituted pyrazino‐pyrido[2,3‐d]pyrimidine‐5,7‐diones (Series A and B) collectively guided the rational design of a novel series of disubstituted analogs bearing a naphthyl moiety, herein designated as Series C. The synthetic approach for these new compounds was based on a previously established protocol developed by our group, employing microwave‐assisted sequential one‐pot coupling reactions between naphthyl derivative **9** and various amino acids. This strategy afforded protected bicyclic intermediates (**11–17**), which underwent final deprotection and intramolecular cyclization to furnish five out of the seven targeted compounds in moderate yields of 30%–31% after HPLC purifications.

The initial in vitro antitumor screening of compounds from Series A and B (50 µM) against BT20 and HGC‐27 cancer cell lines revealed modestly enhanced cytotoxic activity for the Series B derivatives. Although both compound series demonstrated favorable docking scores, Series A generally showed higher predicted affinity for PI3K. In contrast, compounds from Series B exhibited stronger binding interactions with mTOR, indicating a potentially enhanced inhibitory effect on this target. These preliminary findings were instrumental in guiding the design and synthesis of a novel series of compounds, designated as Series C, featuring a bulkier and more hydrophobic naphthyl substituent at the C‐9 position and diverse substituents at C‐6. Molecular docking studies demonstrated a marked improvement in the predicted binding affinity toward PI3K. Moreover, docking scores for the mTOR kinase remained within the same range as those observed for Series A and B. This theoretical potential was subsequently validated through an additional round of in vitro antitumor assays against the same cell lines (HGC‐27, BT20, and CAL‐27), yielding inhibition rates of 85%–94% at 50 µM across all three models for compounds **3** and **4**.

Between the two derivatives, compound **4** demonstrated superior antiproliferative activity compared to compound **3**, exhibiting lower IC_50_ values of 7.8, 6.9, and 12.8 µM against HGC‐27, BT20, and CAL‐27 cancer cell lines, respectively. Moreover, compound **4** showed improved selectivity toward cancer cells over normal oral fibroblasts (GNP5). Additionally, treatment with compounds **3** and **4** at 20 µM induced G1 phase arrest in HGC‐27, BT20, and CAL‐27 cell lines, which is consistent with mTOR inhibition.

The observed effects of compounds **3** and **4** on downstream targets, including ULK1, STAT3, and AKT, together with their modulation of the autophagy process, support the hypothesis that these compounds function as mTOR inhibitors. This confirms their potential as antitumor candidates, influencing both cell proliferation and viability. Further studies are warranted to validate their kinase inhibitory activity, with an emphasis on enzymatic assays and comparative profiling.

## 4 Experimental

### 4.1 Pharmacophore Validation

The 3D structures of the ligands were generated using the online version of the Corina software (https://www.mn‐am.com/online_demos/corina_demo), which assigned predefined bond lengths and angles based on bond type, atomic composition, and hybridization state [40]. To generate the pharmacophore models, the structures of the six dual PI3K/mTOR inhibitors shown in Figure 4 were downloaded from the PDB and uploaded to PharmaGist (http://bioinfo3d.cs.ta.ac.ac.il/PharmaGist/), allowing the program to assign the least flexible structure as the pivot. For visualization of binding modes, the crystallographic complexes of mTOR (PDB code: 4JT6) and PI3K (PDB code: 3IHY) were aligned based on the common catalytic site residues, as described by Poulsen et al., 2004 [41].

### 4.2 Protein Preparation

4XE0 (PI3K) and 4JT5 (mTOR) crystal structures were obtained from the Protein Data Bank database. Structures were prepared with the Protein Preparation Wizard module (Schrödinger v.2021−3) [42]: proteins were preprocessed by default, cosolvent molecules and nonstructural waters were deleted and missing hydrogen and side chains were modeled. Using PROPKA, the ionization state of all the residues was calculated at pH 7.4. Finally, the hydrogen‐bond network was optimized, and the minimization of the whole system was performed with the OPLS3e force field using a default constraint of 0.3 Ä.

### 4.3 Ligand Preparation

Ligands were sketched using 2D panel from Maestro (Schrödinger v.2021−3). Library was prepared with LigPrep: hydrogens were added, protonation states were simulated at pH 7.4 ± 2 with Epik [43], salts were eliminated, and ligand geometry was optimized with the OPLS3e force field.

### 4.4 Docking Methodology

For each protein, a grid box was generated using the cocrystallized ligand as the center. The grid dimensions were set to default values, and ligands were docked using Glide (Schrödinger) [44] with extra precision (XP) mode. Interactions with critical amino acids in the hinge region (Glu826/Val828 for 4XE0 and Glu2238/Val2240 for 4JT5) were used as constraints during the conformational search. When induced‐fit docking was applied (Prime, Schrödinger, v.2021−3) [45], side chains were trimmed based on B‐factor values and subsequently refined after the initial docking step. The conformations of amino acids located within 5.0 Å of the ligand were then minimized. Finally, a redocking step was performed using XP precision.

### 4.5 Synthesis

All reagents, chemicals, and solvents were purchased from commercial suppliers and used without further purification. Reactions were monitored by thin‐layer chromatography (TLC) using commercially available precoated plates and visualized with UV light at 254 nm. Chromatographic purifications on the column were performed using flash silica gel (40–63 μm). Microwave chemical reactor CEM—model: Discover—serial number: DU 8608. The hydrogen nuclear magnetic resonance (^1^H NMR) and carbon (^13^C NMR) spectra were recorded on Bruker Avance DRX 300 (300 MHz), DRX 400 (400 MHz), or DPX 500 (500 MHz) spectrometers at the University of São Paulo ‐ Ribeirão Preto. The chemical shifts (δ) are reported in parts per million (ppm) relative to tetramethyl silane (TMS), with the multiplicity (s = singlet, d = doublet, t = triplet, dd = double doublet, m = multiplet), coupling constant (*J*), given in Hertz (Hz), and the number of hydrogens deduced from the relative integral in parentheses. The assignments described were made with the aid of 2D analyses, *g*COSY, *g*HMQC, and *g*HMBC. Mass spectrometry analyses by ESI‐MS (electrospray Ionization) were performed using positive and negative ionization modes on a Bruker Daltonics UltrOTOF‐Q–ESI‐TOF instrument (USP‐RP). Shimadzu HPLC system with Shim‐PaK C‐18 reverse‐phase column, Shim‐PaK CLC‐ODS (M) (250 x 10.0 mm or 4.6 mm) was applied to further purification under different gradient conditions (A: H_2_O, B: MeCN) and flow rates, with UV detection (190 and 250 nm).

#### 4.5.1 General Protocol for Obtaining Intermediates (11–17)

In different flasks, 2‐amino nicotinic acid (60 mg, 0.43 mmol, 1.0 Eq.) was added, along with N‐Boc‐3‐(2‐naphthyl)‐L‐alanine (140 mg, 0.43 mmol, 1.0 Eq.). Each flask was then added anhydrous pyridine (1.5 mL) and triphenyl phosphite (124 μL, 0.47 mmol, 1.1 Eq.) and then irradiated in a microwave reactor (M.W.) for 10 min at 100°C. After cooling to room temperature, each mixture was then separately added to each methyl ester derived from glycine, alanine, isoleucine, phenylalanine, tryptophan, tyrosine, and methionine (0.43 mmol, 1.0 equiv.) then irradiated again in the M.W. at 150°C for 2.0–2.5 min. After purification by flash CCC with silica gel (hexane 100% → AcOEt 100%), the protected intermediates with the groups ‐*N*Boc and ‐OMe (**11–17**) were obtained as slightly yellowish oils with yields ranging from 25% to 45%. Their structures were subsequently confirmed by spectroscopic analyses (^1^H NMR) and by HRESI‐MS.

**11** (39.2 mg, 0.08 mmol, 37%): %): Microwave irradiation time in the second step: 2,0 min. ^1^H NMR (CDCl_3_, 300 MHz) δ: 9.02 (dd, 1H, *J*_2,3_ 4.4, *J*_2,4_ 1.5 Hz, H‐2), 8.59 (dd, 1H, *J*_3,4_ 8.1 Hz, *J*_2,4_ 1.5 Hz, H‐4), 7.80–7.74 (m, 3H, arom‐H), 7.66 (s, 1H, H‐17), 7.49–7.41 (m, 3H, arom‐H), 7.30 (apparent d, 1H, *J*_3,4_ 8.1 Hz, H‐3), 5.60 (d, 1H, *J*_9, NH_ 9.1 Hz, NH), 5.11 (apparent dd, 1H, *J*_9,15a_ 7.9, *J*_9,15b_ 15.7 Hz, H‐9), 4.99 (d, 1H, *J*_6a, 6b_ 17.8 Hz, H‐6a), 4.79 (d, 1H, *J*_6a, 6b_ 17.8 Hz, H‐6b), 3.74 (s, 3H, OCH3), 3.53 (dd, 1H, *J*_9,15a_ 6.2 Hz, *J*_15a, 15b_ 13.9 Hz, H‐15a), 3.43 (dd, 1H, *J*_9,15b_ 7.9 Hz, *J*_15a, 15b_ 13.8 Hz, H‐15b), 1.29 (s, 3H, NHBoc). HRESI‐MS m/z calcd. for C_27_H_28_N_4_O_5_^+^ [M + H]^+^: 489.2132, found: 489.2129.

**12** (48.9 mg, 0.097 mmol, 44%): Microwave irradiation time in the second step: 2.0 min. NMR ^1^H (CDCl_3_, 300 MHz) δ: 8.98 (dd, 1H, *J*_2,3_ 4.6 Hz, *J*_2,4_ 2.0 Hz, H‐2), 8.52 (dd, 1H, *J*_3,4_ 7.8 Hz, *J*_2,4_ 2.0 Hz, H‐4), 7.77–7.63 (m, 4H, arom‐H), 7.44–7.38 (m, 3H, H‐3, 2 arom‐H), 7.33 (dd, 1H, *J*_17,22_ 1.4 Hz, *J*_17,23_ 8.4 Hz, H‐17), 5.60 (d, 1H, *J*_9, NH_ 9.4 Hz, NH), 5.35 (apparent dd, 1H, *J*_9,15a_ 7.1 Hz, *J*_9,15b_ 16.8 Hz, H‐9), 4.93 (q, 1H, *J*_6,8_ 6.7 Hz, H‐6), 3.58 (dd, 1H, *J*_9,15a_ 6.9 Hz, *J*_15a, 15b_ 13.8 Hz, H‐15a), 3.44–3,39 (m, 1H, H‐15b), 3.35 (s, 3H, OCH_3_), 1.70 (d, 3H, *J*_6,8_ 6.7 Hz, CgH_3_−8), 1.30 (s, 9H, N*H*Boc). HRESI‐MS m/z calcd. para C_28_H_31_N_4_O_5_^+^ [M + H]^+^ 503.2289, found 503.2289.

**13** (29.7 mg, 0.055 mmol, 25%): Microwave irradiation time in the second step: 2.0 min. NMR ^1^H (CDCl_3_, 300 MHz) δ: 8.98 (dd, 1H, *J*_2,3_ 4.5 Hz, *J*_2,4_ 1.9 Hz, H‐2), 8.53 (dd, 1H, *J*_3,4_ 8.2 Hz, *J*_2,4_ 1.8 Hz, H‐4), 7.76–7.69 (m, 3H, arom‐H), 7.62 (s, 1H, H‐17), 7.46–7.39 (m, 3H, 3 arom‐H), 7.32 (apparent d, 1H, *J*_3,4_ 8.2 Hz, *J*_3,4_ 8.0 Hz, H‐3), 5.81 (d, 1H, *J*_9, NH_ 9.5 Hz, NH), 5.41 (apparent dd, 1H, *J*_9,15a_ 6.9 Hz, *J*_9,15b_ 15.9 Hz, H‐9), 4.62–4.53 (m, 1H, H‐6), 3.59 (dd, 1H, *J*_9,15a_ 6.9 Hz, *J*_15a, 15b_ 13.9 Hz, H‐15a), 3.44–3.39 (m, 1H, H‐15b), 3.32 (s, 3H, OCH_3_), 2.66–2.56 (m, 1H, CH_2_−8), 1.31 (s, 9H, N*H*Boc), 1.13–1.10 (m, 2H, CH_2_−26), 0.93 (t, 3H, *J*_26,27_ 7.3 Hz, CH_3_−26), 0.80 (d, 3H, *J*_8,28_ 6.7 Hz, CH_3_−27). HRESI‐MS m/z calcd. para C_31_H_37_N_4_O_5_^+^ [M + H]^+^ 545.2758, found 545.2751.

**14** (32.6 mg, 0.056 mmol, 26%): Microwave irradiation time in the second step: 2.0 min. NMR ^1^H (CDCl_3_, 300 MHz) δ: 8.92 (dd, 1H, *J*_2,3_ 4.6 Hz, *J*_2,4_ 2.0 Hz, H‐2), 8.60 (dd, 1H, *J*_3,4_ 8.2 Hz, *J*_2,4_ 2.0 Hz, H‐4), 7.74–7.71 (m, 1H, arom‐H), 7.67–7.64 (m, 2H, arom‐H), 7.50–7.43 (m, 3H, arom‐H), 7.40–7.36 (m, 2H, arom‐H), 7.20 (apparent d, 1H, *J*_3,4_ 8.2 Hz, H‐3), 7.13–7.09 (m, 3H, arom‐H), 7.02–6.99 (m, 2H, arom‐H), 5.70 (d, 1H, *J*_9, NH_ 8.2 Hz, NH), 5.06–4.97 (m, 1H, H‐9), 4.87 (dd, 1H, *J*_6,8a_ 6.0 Hz, *J*_6,8b_ 14.2 Hz, H‐6), 3.70–3.62 (m, 2H, CH_2_−8), 3.32 (s, 3H, OCH_3_), 3.35 (dd, 1H, *J*_9,15a_ 5.9 Hz, *J*_15a, 15b_ 13.7 Hz, H‐15a), 3.16 (dd, 1H, *J*_9,15b_ 6.0 Hz, *J*_15a, 15b_ 13.7 Hz, H‐15b), 1.34 (s, 9H, N*H*Boc). HRESI‐MS m/z calcd. para C_34_H_35_N_4_O_5_^+^ [M + H]^+^ 579.2602, found 579.2599.

**15** (50.3 mg, 0.081 mmol, 37%). Microwave irradiation time in the second step: 2.5 min. NMR ^1^H (CD_3_OD, 300 MHz) δ: 8.66 (dd, 1H, *J*_2,3_ 4.6 Hz, *J*_2,4_ 1.8 Hz, H‐2), 8.60 (dd, 1H, *J*_3,4_ 8.1 Hz, *J*_2,4_ 1.8 Hz, H‐4), 8.48 (dd, 1H, *J*_22,23_ 6.0 Hz, *J*_17,22_ 1.6 Hz, H‐22), 7.51–7.45 (m, 3H, arom‐H), 7.40–7.33 (m, 3H, arom‐H), 7.30–7.27 (m, 1H, arom‐H), 7.10 (dd, 1H, *J*_3,4_ 8.1 Hz, *J*_2,3_ 6.0 Hz, H‐3), 6.94 (s, 1H, H‐27), 6.82 (t, 1H, *J* 7.7 Hz, arom‐H), 6.55 (t, 1H, *J* 7.5 Hz, arom‐H), 5.48–5.43 (m, 1H, H‐6), 4,82 (apparent t, 1H, *J*_9,15a =_ *J*_9,15b_ 5.3 Hz, H‐9), 3.73 (s, 3H, OCH_3_), 3.27–3.24 (m, 3H, H‐15a, H‐8a, H‐8b), 3.09 (dd, 1H, *J*_9,15b_ 5.1 Hz, *J*_15a, 15b_ 13.7 Hz, H‐15b), 1.33 (s, 9H, N*H*Boc). HRESI‐MS m/z calcd. para C_36_H_36_N_5_O_5_^+^ [M + H]^+^ 618.2711, found 618.2707.

**16** (58.6 mg, 0.099 mmol, 45%). Microwave irradiation time in the second step: 2.5 min. NMR ^1^H (CD_3_OD, 300 MHz) δ: 8.76 (dd, 1H, *J*_2,3_ 4.7 Hz, *J*_2,4_ 1.9 Hz, H‐2), 8.59 (dd, 1H, *J*_3,4_ 8.0 Hz, *J*_2,4_ 1.9 Hz, H‐4), 7.69–7.65 (m, 1H, arom‐H), 7.50–7.48 (m, 3H, arom‐H), 7.45 (s, 1H, H‐17), 7.41–7.36 (m, 1H, arom‐H), 7.34–7.30 (m, 2H, arom‐H), 7.09 (apparent d, 1H, *J*_3,4_ 8.0 Hz, H‐3), 6.6 (d, 1H, *J*_27,28 __=_ *J*_30,31_ 8.4 Hz, H‐27 and H‐31), 6.42 (d, 1H, *J*_27,28 __=_ *J*_30,31_ 8.4 Hz, H‐28 and H‐30), 5.49–5.42 (m, 1H, H‐6), 4.95 (apparent t, *J*_9,*15a *_= *J*_9,*15b*_ 5.4 Hz, H‐9), 3.69 (s, 3H, OMe), 3.50 (dd, 1H, *J*_15a, 15b_ 13.5 Hz, *J*_9,15a_ 4.4 Hz, H‐15a), 3.42–3.27 (m, 2H, H‐8a, H‐8b), 3.13 (dd, 1H, *J*_15a, 15b_ 13.5 Hz, *J*_9,15b_ 5.8 Hz, H‐15b), 1.35 (s, 9H, NHBoc). HRESI‐MS m/z calcd. para C_34_H_35_N_4_O_6_^+^ [M + H]^+^ 595.2551, found 595.2538.

**17** (40.2 mg, 0.072 mmol, 33%): Microwave irradiation time in the second step: 2.0 min. NMR ^1^H (CD_3_OD, 300 MHz) δ: 8.94 (dd, 1H, *J*_2,3_ 4.6 Hz, *J*_2,4_ 1.9 Hz, H‐2), 8.53 (dd, 1H, *J*_3,4_ 8.0 Hz, *J*_2,4_ 1.6 Hz, H‐4), 7.79 (s, 1H, H‐17), 7.75–7.67 (m, 3H, arom‐H), 7.54 (dd, 1H, *J*_3,4_ 8.0 Hz, *J*_2,3_ 4.6 Hz, H‐3), 7.49–7.40 (m, 1H, arom‐H), 7.39–7.33 (m, 2H, arom‐H), 5.57 (apparent s, 1H, H‐6), 4.00 (apparent t, 1H, *J*_9,15a _= *J*_9,15b_ 6.6 Hz, H‐9), 3.71–3.64 (m, 1H, H‐15a), 3.59 (s, 3H, OMe), 3.45 (dd, 1H, *J*_9,15b_ 8.8 Hz, *J*_15a, 15b_ 13.4 Hz, H‐15b), 2.64–2.55 (m, 1H, H‐26b), 2.35–2.27 (m, 1H, H‐26a), 1.90 (s, 3H, S‐CH_3_), 1.58–1.50 (m, 1H, H‐8b), 1.34 (dd, 1H, *J*_26,8a_ 7.5 Hz, *J*_8a, 8b_ 15.1 Hz, H‐8a), 1.17 (s, 9H, N*H*Boc). HRESI‐MS m/z calcd. para C_30_H_35_N_4_O_5_S^+^ [M + H]^+^ 563.2323, found 563.2320.

#### 4.5.2 General Protocol for Synthesis of Final Products (1–7)

Each protected intermediate (**11–17**) was treated with a mixture of TFA/DCM in a 1:1 v/v ratio (2.0 mL) and stirred at room temperature for 2.0 h. Subsequently, the solvents were removed under reduced pressure, and the crude reaction mixtures were neutralized with a 5% NaHCO_3_ solution until pH ~ 7.0 (~2.0 mL). Finally, DCM was added, and extraction was performed using a separation funnel. The organic phase was separated, dried, and purified by CCC using flash silica gel (hexane 100% → AcOEt 100%), followed by purification using Shimadzu Shim‐PaK HPLC system under gradient conditions (A: H_2_O, B: MeCN, 0‐80% B) and of 1.0 min^.–1^. In this manner, the final products of the series (**1–4,7**) were obtained as slightly yellow oils with yields around 30%. These compounds had their structures confirmed and signals assigned by a set of spectroscopic analyses (^1^H NMR, *g*COSY, *g*HMQC, *g*HMBC) and by HRESI‐MS.

**9‐(Naphthalen‐2‐ylmethyl)−8,9‐dihydro‐5H‐pyrazino[1,2‐a]pyrido[2,3‐d]pyrimidine‐5,7(6H)‐dione 1** (5.5 mg, 0.015 mmol, 30%). ^1^H NMR (DMSO‐d_6_, 500 MHz) δ: 9.01 (dd, 1H, *J*_2,3_ 4.5 Hz, *J*_2,4_ 1.9 Hz, H‐2), 8.80 (d, 1H, *J*_9, NH_ 3.8 Hz, NH), 8.53 (dd, 1H, *J*_3,4_ 7.9 Hz, *J*_2,4_ 1.9 Hz, H‐4), 7.89 (dd, 1H, *J*_18,19_ 6.0 Hz, *J*_18,20_ 3.3 Hz, H‐18), 7.84–7.75 (m, 2H, H‐22, H‐21), 7.69 (s, 1H, H‐17), 7.59 (dd, 1H, *J*_3,4_ 7.9 Hz, *J*_2,3_ 4.6 Hz, H‐3), 7.49 (dd, 2H, J_18,19 _= J_20,21_ 6.0 Hz, *J*_18,20 =_ *J*_19,21_ 3.3 Hz, H‐19, H‐20), 7.26 (dd, 1H, *J*_22,23_ 8.4 Hz, *J*_21,23_ 1.4 Hz, H‐23), 4.90 (dd, 1H, *J*_9,15a_ 9.7 Hz, *J*_9,15b_ 5.8 Hz, H‐9), 4,41 (d, 1H, *J*_6a, 6b_ 17.8 Hz, H‐6a), 3.62 (d, *J*_6a, 6b_ 17.8 Hz, H‐6b). ^13^C NMR (CDCl_3_, 150 MHz) δ: 168.8 (C‐7), 165.0 (C‐5), 157.3 (C‐11), 156.7 (C‐2), 145.2 (C‐14), 138.2–123.1 (C‐3, C‐4, C‐16, C‐17, C‐18, C‐19, C‐21, C‐22, C‐23, C‐24, C‐25), 115.5 (C‐12), 57.3 (C‐9), 45.7 (C‐6), 29.9 (C‐15). HRMS (ESI) *m/z* calcd. for C_21_H_17_N_4_O_2_^+^ [M + H]^+^ 357.1346, found 357.1349. Under the reported chromatographic conditions, the compound's retention time (TR) was 14.79 min.

**6‐Methyl‐9‐(naphthalen‐2‐ylmethyl)−8,9‐dihydro‐5H‐pyrazino[1,2‐a]pyrido[2,3‐d]pyrimidine‐5,7(6H)‐dione 2** (4.0 mg, 0.011 mmol, 30%). ^1^H NMR (CDCl_3_, 400 MHz) δ: 9.04 (dd, 1H, *J*_2,3_ 4.6 Hz, *J*_2,4_ 2.0 Hz, H‐2), 8.65 (dd, 1H, *J*_3,4_ 7.9 Hz, *J*_2,4_ 2.0 Hz, H‐4), 7.89 (d, 1H, *J*_22,23_ 8.4 Hz, H‐22), 7.87–7.80 (m, 2H, H‐18, H‐21), 7.75 (s, 1H, H‐17), 7.53–7.50 (m, 3H, H‐3, H‐19, H‐20), 7.41 (dd, 1H, *J*_22,23_ 8.4 Hz, *J*_21,23_ 1.7 Hz, H‐23), 6.03 (apparent s, 1H, NH), 5.40 (q, 1H, *J*_6,26_ 7.2 Hz, H‐6), 4.96 (dd, 1H, *J*_9,15a_ 10.6 Hz, *J*_9,15b_ 3.5 Hz, H‐9), 4.43 (dd, 1H, *J*_15a, 15b_ 14.4 Hz, *J*_9,15b_ 3.5 Hz, H‐15b), 3.18 (dd, 1H, *J*_15a, 15b_ 14.4 Hz, *J*_9,15a_ 10.6 Hz, H‐15a), 1.61 (d, 3H, *J*_6,26_ 7.2 Hz, C*H*_3_−26). ^13^C NMR (CDCl_3_, 150 MHz) δ: 170.2 (C‐7), 165.9 (C‐5), 157.2 (C‐11), 156.7 (C‐2), 147.9 (C‐14), 137.4–119.0 (C‐3, C‐4, C‐12, C‐16, C‐17, C‐18, C‐19, C‐20, C‐21, C‐22, C‐23, C‐24, C‐25), 53.3 (C‐9), 52.3 (C‐6), 29.8 (C‐15), 19.3 (C‐26). HRMS (ESI) *m/z* calcd. for C_22_H_17_N_4_O_2_^−^ [M − H]^+^ 369.1357, found 369.1328. Under the reported chromatographic conditions, the compound's TR was 15.77 min.

**6‐(sec‐Butyl)‐9‐(naphthalen‐2‐ylmethyl)−8,9‐dihydro‐5H‐pyrazino[1,2‐a]pyrido[2,3‐d]pyrimidine‐5,7(6H)‐dione 3** (6.0 mg, 0.014 mmol, 31%). ^1^H NMR (CDCl_3_, 400 MHz) δ: 9.04 (dd, 1H, *J*_2,3_ 4.6 Hz, *J*_2,4_ 2.0 Hz, H‐2), 8.65 (dd, 1H, *J*_3,4_ 8.0 Hz, *J*_2,4_ 2.0 Hz, H‐4), 7.89 (d, 1H, *J*_22,23_ 8.4 Hz, H‐22), 7.86–7.80 (m, 2H, H‐18, H‐21), 7.74 (s, 1H, H‐17), 7.53–7.49 (m, 3H, H‐3, H‐19, H‐20), 7.41 (dd, 1H, *J*_22,23_ 8.4 Hz, *J*_21,23_ 1.7 Hz, H‐23), 6.02 (apparent s, 1H, NH), 5.32 (d, 1H, *J*_6,26_ 6.8 Hz, H‐6), 5.02 (dd, 1H, *J*_9,15a_ 10.6 Hz, *J*_9,15b_ 3.5 Hz, H‐9), 4.42 (dd, 1H, *J*_15a, 15b_ 14.3 Hz, *J*_9,15b_ 3.4 Hz, H‐15b), 3.10 (dd, 1H, *J*_15a, 15b_ 14.4 Hz, *J*_9,15a_ 10.7 Hz, H‐15a), 2.06–1.98 (m, 1H, H‐26), 1.61–1.52 (m, 1H, H‐27a), 1.36–1.29 (m, 1H, H‐27b), 1.01 (d, 3H, *J*_26,29_ 6.8 Hz, C*H*_3_−29), 0.96 (t, 3H, *J*_27,28_ 7.4 Hz, C*H*_3_−28). ^13^C NMR (CDCl_3_, 150 MHz) δ: 166.8 (C‐7), 160.7 (C‐5), 157.0 (C‐2), 154.9 (C‐11), 149.1 (C‐14), 137.2–118.4 (C‐3, C‐4, C‐12, C‐16, C‐17, C‐18, C‐19, C‐20, C −21, C‐22, C‐23, C‐24, C‐25), 58.9 (C‐6), 54.8 (C‐9), 38.7 (C‐15), 29.6 (C‐26), 26.4 (C‐27), 15.3 (C‐29), 11.3 (C‐28). HRMS (ESI) *m/z* calcd. for C_25_H_23_N_4_O_2_^+^ [M − H]^+^ 411.1826, found 411.1803. Under the reported chromatographic conditions, the compound's TR was 18.26 min.

**6‐Benzyl‐9‐(naphthalen‐2‐ylmethyl)−8,9‐dihydro‐5H‐pyrazino[1,2‐a]pyrido[2,3‐d]pyrimidine‐5,7(6H)‐dione 4** (5.5 mg, 0.012 mmol, 31%). ^1^H NMR (CDCl_3_, 400 MHz) δ: 9.06 (dd, 1H, *J*_2,3_ 4.6 Hz, *J*_2,4_ 2.0 Hz, H‐2), 8.72 (dd, 1H, *J*_3,4_ 7.9 Hz, *J*_2,4_ 2.0 Hz, H‐4), 7.86–7.75 (m, 3H, H‐18, H‐21, H‐22), 7.55 (dd, 1H, *J*_3,4_ 7.9 Hz, *J*_2,3_ 4.6 Hz, H‐3), 7.53–7.50 (m, 2H, H‐19, H‐20), 7.40 (s, 1H, H‐17), 7.21 (t, 2H, *J*_28,29 =_ *J*_29,30_ / *J*_30,31 _= *J*_31,32_ 7.7 Hz, H‐29, H‐30), 6.99 (dd, 1H, *J*_22,23_ 8.4 Hz, *J*_21,23_ 1.7 Hz, H‐23), 6.89 (apparent d, 2H, *J*_28,29_ / *J*_31,32_ 7.0 Hz, H‐28, H‐32), 5.70 (apparent s, 1H, NH), 5.58 (t, 1H, *J*_6,26a =_ *J*_6,26b_ 4.2 Hz, H‐6), 4.04 (dd, 1H, *J*_9,15a_ 14.2 Hz, *J*_9,15b_ 3.2 Hz, H‐9), 3.45–3.43 (m, 2H, H‐26a, H‐26b), 2.98 (dd, 1H, *J*_15a, 15b_ 11.0 Hz, *J*_9,15b_ 3.2 Hz, H‐15b), 2.78 (dd, 1H, *J*_15a, 15b_ 14.3 Hz, *J*_9,15a_ 11.0 Hz, H‐15a). ^13^C NMR (CDCl_3_, 150 MHz) δ: 167.7 (C‐7), 161.1 (C‐5), 156.6 (C‐2), 154.8 (C‐11), 148.5 (C‐14), 137.2–123.0 (C‐3, C‐4, C‐16, C‐17, C‐18, C‐19, C‐20, C‐21, C‐22, C‐23, C‐24, C‐25, C‐27, C‐28, C‐29, C‐30, C‐31, C‐32), 115.7 (C‐12), 58.1 (C‐9), 53.4 (C‐6), 38.1 (C‐26). 36.9 (C‐15). HRMS (ESI) *m/z* calcd. for C_28_H_21_N_4_O_2_^−^ [M − H]^+^ 445.1670, found 445.1652. Under the reported chromatographic conditions, the compound's TR was 18.14 min.

**6‐(2‐(Methylthio)ethyl)‐9‐(naphthalen‐2‐ylmethyl)−8,9‐dihydro‐5H‐pyrazino[1,2‐a]pyrido[2,3‐d]pyrimidine‐5,7(6H)‐dione 7** (4.9 mg, 0.011 mmol, 30%). ^1^H NMR (CDCl_3_, 400 MHz) δ: 9.05 (dd, 1H, *J*_2,3_ 4.6 Hz, *J*_2,4_ 2.0 Hz, H‐2), 8.65 (dd, 1H, *J*_3,4_ 7.9 Hz, *J*_2,4_ 2.0 Hz, H‐4), 7.90 (d, 1H, *J*_22,23_ 8.4 Hz, H‐22), 7.88–7.80 (m, 2H, H‐18, H‐21), 7.75 (s, 1H, H‐17), 7.54–7.50 (m, 3H, H‐3, H‐19, H‐20), 7.41 (dd, 1H, *J*_22,23_ 8.4 Hz, *J*_21,23_ 1.7 Hz, H‐23), 6.02 (apparent s, 1H, NH), 5.47 (t, 1H, *J*_6,15a =_ *J*_6,15b_ 7.0 Hz, H‐6), 5.01 (dd, 1H, *J*_9,15a_ 10.7 Hz, *J*_9,15b_ 3.5 Hz, H‐9), 4.42 (dd, 1H, *J*_15a, 15b_ 14.4 Hz, *J*_9,15b_ 3.5 Hz, H‐15b), 3.16 (dd, 1H, *J*_15a, 15b_ 14.4 Hz, *J*_9,15a_ 10.7 Hz, H‐15a), 2.69–2.64 (m, 2H, CH_2_−25), 2.28–2.20 (m, 2H, CH_2_−26), 2.07 (S, 3H, SC*H*_3_−29). ^13^C NMR (CDCl_3_, 150 MHz) δ: 168.2 (C‐7), 161.0 (C‐5), 156.7 (C‐2), 153.8 (C‐11), 147.0 (C‐14), 137.3–123.0 (C‐3, C‐4, C‐16, C‐17, C‐18, C‐19, C‐20, C‐21, C‐22, C‐23, C‐24, C‐25), 116.3 (C‐12), 55.7 (C‐9), 44.3 (C‐6), 38.7 (C‐15), 31.2 (C‐26), 29.9 (C‐27), 14.3 (C‐29). HRMS (ESI) *m/z* calcd. for C_24_H_21_N_4_O_2_S^−^ [M − H]^+^ 429.1391, found 429.1407. Under the reported chromatographic conditions, the compound's TR was 17.21 min.

### 4.6 Biological Assays

#### 4.6.1 Cultivation Conditions for Cancer Cell Lines

The normal gingival fibroblast lineages [46], gastric carcinoma HGC‐27 (Bank of cells of Rio de Janeiro, BCRJ), tongue squamous cell carcinoma CAL‐27 (ATCC: CRL‐2095, EUA), and breast carcinoma BT‐20 (ATCC: HTB‐19, EUA) were cultured in DMEM medium (DMEM, Sigma–Aldrich, St. Louis, MO, USA) supplemented with 10% fetal bovine serum, 100 U/mL penicillin, and 100 μg/mL streptomycin. The cultures were maintained in a humidified incubator with 5% CO_2_ at 37°C.

#### 4.6.2 Cell Viability Assay with Series A and B

The resazurin assay was used to determine cell viability. The cells were seeded in 96‐well plates (1,000 cells/well) and treated with different compounds for 72 h in culture medium containing 2% fetal bovine serum. After treatment, the culture medium was replaced with phenol‐free DMEM medium containing resazurin (44 µM in PBS; Sigma–Aldrich), and the plates were incubated in the dark for 4 h at 37°C. Finally, the cell viabilities of the BT‐20 and HGC‐27 lineages were measured by fluorescence detection (excitation 530/25 nm, emission 590/35 nm) using a Synergy 2 microplate fluorometer (Biotek).

#### 4.6.3 Cell Viability Assay with Series C ‐ naphthyl Analogs (1–4,7)

The viability of CAL‐27, HGC‐27, and BT‐20 cells was measured using the resazurin assay. For this, cells were plated in 96‐well plates (1,000 cells/well) and incubated at 37°C and 5% CO_2_ for 16–24 h to allow cell adhesion. Subsequently, the cells were treated with different compounds (0–50 µM) for 72 h. After treatment, the medium containing the compounds was replaced with phenol‐free DMEM medium (Sigma–Aldrich) containing resazurin at a concentration of 44 µM (Sigma–Aldrich). Following incubation for 4 h at 37°C, fluorescence detection (excitation 530/25 nm, emission 590/35 nm) was performed using a microplate fluorometer (Synergy 2, Biotek). Independent biological experiments were conducted in quadruplicate. The data were plotted, and the IC_50_ value was estimated using nonlinear regression.

#### 4.6.4 Cell Cycle Assay

The CAL‐27, HGC‐27, and BT‐20 cells were seeded in 10 cm plates (1.5 × 10^6^ cells/plate) and incubated in an incubator for 16–24 h. Subsequently, the cells were treated for 24 h with compounds **1** and **4** at a concentration of 20 µM and with Gedatolisib at 2.5 µM. After treatment, the cells were collected by centrifugation and fixed in 70% ethanol for 1 h at 4°C. Following a wash with PBS, the cells were treated with RNase A (100 μg/mL, Sigma–Aldrich) for 30 min at 37°C and stained with propidium iodide (50 μg/mL) for 15 min at 37°C. Finally, the percentages of cells in each phase of the cell cycle were determined using a FACSCalibur flow cytometer (BD Biosciences ‐ San Jose, CA, USA). The cell cycle distribution was analyzed using the ModFit LT V3.3 software.

#### 4.6.5 Western Blot

The CAL‐27, HGC‐27, and BT‐20 cells were seeded in 10 cm plates (2 × 10^6^ cells/plate) and incubated in an incubator for 16–24 h. Subsequently, the cells were treated with rapamycin (10 µM), **3** and **4** (20 µM) for 6 h. In the conditions with chloroquine (20 µM), chloroquine was added 30 min before treatment and then maintained with the other compounds for up to 6 h. After treatment, the cells were collected, and proteins were isolated. The electrophoretically resolved proteins were transferred to a PVDF membrane (polyvinylidene fluoride ‐ GE Healthcare) using the PowerBlotter Station (Invitrogen) following the manufacturer's recommendations. After transfer, the membranes were blocked with a 5% (w/v) skim milk solution in TBS‐T (0.1 M Tris (pH 7.5), 0.9% NaCl, 0.1% Tween‐20) at room temperature for 1 h under agitation. After blocking, the membranes were washed with TBS‐T for 30 s and incubated in TBS‐T containing the primary antibody for 16 h at 4°C under agitation. Following incubation with the primary antibody, the membranes were washed with TBS‐T and incubated at room temperature for 1 h in a solution of TBS‐T and 5% skim milk containing the peroxidase‐conjugated secondary antibody. After washing with TBS‐T, the membranes were incubated with the SuperSignal West Dura substrate. For the detection of immunostaining, the membranes were developed using the ChemiDoc photodocumentation system (Bio‐Rad).

## Supporting Information

Additional supporting information can be found online in the Supporting Information Section. **Supporting Fig. S1**: ^1^H NMR spectrum (300 MHz, CDCl_3_) of 11. **Supporting Fig. S2:** HRESI‐MS spectrum of 11 (m/z calcd. for C_27_H_28_N_4_O_5_+ [M+H]^+^ 489.2132, found 489.2129). **Supporting Fig. S3:** ^1^H NMR spectrum (300 MHz, CDCl_3_) of 12. **Supporting Fig. S4:** HRESI‐MS spectrum of 12 (m/z calcd. for C_28_H_31_N_4_O_5_+ [M+H]^+^ 503.2289, found 503.2289). **Supporting Fig. S5:** ^1^H NMR spectrum (300 MHz, CDCl_3_) of 13. **Supporting Fig. S6:** HRESI‐MS spectrum of 13 (m/z calcd. for C_31_H_37_N_4_O_5_+ [M+H]_+_ 545.2758, found 545.2751). **Supporting Fig. S7:** ^1^H NMR spectrum (300 MHz, CDCl_3_) of 14. **Supporting Fig. S8:** HRESI‐MS spectrum of 14 (m/z calcd. for C_34_H_35_N_4_O_5_+ [M+H]^+^ 579.2602, found 579.2599). **Supporting Fig. S9:** ^1^H NMR spectrum (300 MHz, CDCl_3_) of 15. **Supporting Fig. S10:** HRESI‐MS spectrum of 15 (m/z calcd. for C_36_H_36_N_5_O_5_+ [M+H]^+^ 618.2711, found 618.2707). **Supporting Fig. S11:** ^1^H NMR spectrum (300 MHz, CDCl_3_) of 16. **Supporting Fig. S12:** HRESI‐MS spectrum of 16 (m/z calcd. for C_34_H_35_N_4_O_6_+ [M+H]^+^ 595.2551, found 595.2538). **Supporting Fig. S13:** ^1^H NMR spectrum (300 MHz, CDCl_3_) of 17. **Supporting Fig. S14:** HRESI‐MS spectrum of 17 (m/z calcd. for C_30_H_35_N_4_O_5_S+ [M+H]^+^ 563.2323, found 563.2320). **Supporting Fig. S15:** ^1^H NMR spectrum (400 MHz, DMSO‐d6) of 1. **Supporting Fig. S16:** gGMQC NMR spectrum (150 MHz, CDCl_3_) of 1. **Supporting Fig. S17:** gGMBC spectrum (150 MHz, CDCl_3_) of 1. **Supporting Fig. S18:** ^13^C NMR spectrum (150 MHz, CDCl_3_) of 1. **Supporting Fig. S19:** HRESI‐MS spectrum of 1 (m/z calcd. for C_21_H_17_N_4_O_2_+ [M+H]^+^ 357.1346, found 357.1349). **Supporting Fig. S20:** Chromatogram of 1 after purification by HPLC (RT = 14.79 min). **Supporting Fig. S21:** ^1^H NMR spectrum (400 MHz, CDCl_3_) of 2. **Supporting Fig. S22:** gGMQC NMR spectrum (150 MHz, CDCl_3_) of 2. **Supporting Fig. S23:** gGMBC spectrum (150 MHz, CDCl3) of 2. **Supporting Fig. S24:** ^13^C NMR spectrum (150 MHz, CDCl3) of 2. **Supporting Fig. S25:** HRESI‐MS spectrum of 2 (m/z calcd. for C_22_H_17_N_4_O_2_‐ [M‐H]+ 369.1357, found 369.1328). **Supporting Fig. S26:** Chromatogram of 2 after purification by HPLC (RT = 15.72 min). **Supporting Fig. S27:** ^1^H NMR spectrum (400 MHz, CDCl3) of 3. **Supporting Fig. S28:** gGMQC NMR spectrum (150 MHz, CDCl_3_) of 3. **Supporting Fig. S29:** gGMBC spectrum (150 MHz, CDCl3) of 3. **Supporting Fig. S30:** ^13^C NMR spectrum (150 MHz, CDCl_3_) of 3. **Supporting Fig. S30:** ^13^C NMR spectrum (150 MHz, CDCl_3_) of 3. **Supporting Fig. S31:** HRESI‐MS spectrum of 3 (m/z calcd. for C_25_H_23_N_4_O_2_‐ [M‐H]^+^ 411.1826, found 411.1803). **Supporting Fig. S32:** Chromatogram of 3 after purification by HPLC (RT = 18.26 min). **Supporting Fig. S33:** ^1^H NMR spectrum (400 MHz, CDCl_3_) of 4. **Supporting Fig. S34:** gGMQC NMR spectrum (150 MHz, CDCl_3_) of 4. **Supporting Fig. S35:** gGMBC spectrum (150 MHz, CDCl_3_) of 4. **Supporting Fig. S36:** ^13^C NMR spectrum (150 MHz, CDCl_3_) of 4. **Supporting Fig. S37:** HRESI‐MS spectrum of 4 (m/z calcd. for C_28_H_21_N_4_O_2_‐ [M‐H]+ 445.1670, found 445.1652). **Supporting Fig. S38:** Chromatogram of 4 after purification by HPLC (RT = 18.14 min). **Supporting Fig. S39:** ^1^H NMR spectrum (400 MHz, CDCl_3_) of 7. **Supporting Fig. S40:** gGMQC NMR spectrum (150 MHz, CDCl_3_) of 7. **Supporting Fig. S41:** gGMBC spectrum (150 MHz, CDCl_3_) of 7. **Supporting Fig. S42:** ^13^C NMR spectrum (150 MHz, CDCl_3_) of 7. **Supporting Fig. S43:** HRESI‐MS spectrum of 7 (m/z calcd. C_24_H_21_N_4_O_2_S‐ [M‐H]^+^ 429.1391, found 429.1407). **Supporting Fig. S44:** Chromatogram of 7 after purification by HPLC (RT = 17.21 min). **Supporting Fig. S45:** Results from cell viability (inhibition) assays in HGC‐27 and BT20 tumor cell lines following 72‐hour exposure to series A and B derivatives (50 μM). **Supporting** **Fig.** **S46:** Results from cell viability (inhibition) assays in HGC‐27, BT20 and BT20 tumor cell lines following 72‐hour exposure to series C derivatives (50 μM). **Supporting Fig. S47:** Cell cycle phases analysis from tumor cell lines (BT20, CAL‐27 and HGC‐27) after 24 hours‐exposure with compounds 3 and 4 (20 µM) and with gedatolisib (2.5 µM).

## Funding

This study was supported by Fundação de Amparo à Pesquisa do Estado de São Paulo (Grant 2018/13518‐1), Conselho Nacional de Desenvolvimento Científico e Tecnológico (Grant 420395/2018‐0, 307125/2025‐4, 309871/2021‐2), Coordenação de Aperfeiçoamento de Pessoal de Nível Superior (Grant 001), Instituto de Salud Carlos III (Grant CB18/05/00040), and Ministerio de Ciencia, Innovación y Universidades (Grant FPU20/03743).

## Conflicts of Interest

The authors declare that they have no known competing financial interests or personal relationships that could have appeared to influence the work reported in this paper.

## Supporting information

Supplementary Material

## Data Availability

Supplementary material (detailed NMR spectra, HRMS‐ESI analysis, chromatographic data and cell viability data) can be found online.
