# Supplementary material for: Design, Synthesis, and Evaluation of Naphthyl Pyrazino‐Pyrido‐Pyrimidinones Targeting the Phosphoinositide 3‐Kinase/Alpha‐Serine/Protein Kinase B/Mammalian Target of Rapamycin Pathway
Source: ChemistryOpen. 2026 Feb 12;15(2):e202500615. doi: 10.1002/open.202500615 (PMC12895471; doi:10.1002/open.202500615)
Supplement: Supplementary file 1 — Supplementary Material [file OPEN-15-e202500615-s001.pdf]

Supplementary Information

Design, Synthesis of Novel Naphthyl Pyrazino-pyrido-pyrimidinones Targeting the PI3K/AKT/mTOR Pathway

Marcelo F. Marchiori[a], Gabriel da Silva[a], Daniel Fábio Kawano[b], Andréia Machado Leopoldino[a], Enrique Madruga[c,d], Ana Martinez [c,d], and Ivone Carvalho[a*]

[a] *School of Pharmaceutical Sciences of Ribeirão Preto - University of São Paulo, 14040-903 Ribeirão Preto-SP, Brazil.*

[b] *Faculty of Pharmaceutical Sciences, State University of Campinas, 13083-970, Campinas-SP, Brazil.*

[c] *Centro de Investigaciones Biológicas (CIB) del Consejo Superior de Investigaciones Científicas (CSIC), Ramiro de Maeztu 9, 28040 Madrid, Spain.*

[d] *Centro de Investigación Biomédica en Red en Enfermedades Neurodegenerativas, (CIBERNED), Instituto de Salud Carlos III, Av. Monforte de Lemos, 3-5, 28029 Madrid, Spain.*

*carronal@usp.br

List of Figures

Figure S1. ^{1}H NMR spectrum of **11**.

Figure S2. HRESI-MS spectrum of **11**.

Figure S3. ^{1}H NMR spectrum of **12**.

Figure S4. HRESI-MS spectrum of **12**.

Figure S5. ^{1}H NMR spectrum of **13**.

Figure S6. HRESI-MS spectrum of **13**.

Figure S7. ^{1}H NMR spectrum of **14**.

Figure S8. HRESI-MS spectrum of **14**.

Figure S9. ^{1}H NMR spectrum of **15**.

Figure S10. HRESI-MS spectrum of **15**.

This is an open-access article distributed under the terms of the Creative Commons Attribution Licence.

Figure S11. ^{1}H NMR spectrum of **16**.

Figure S12. HRESI-MS spectrum of **16**.

Figure S13. ^{1}H NMR spectrum of **17**.

Figure S13. HRESI-MS spectrum of **17**.

Figure S14. ^{1}H NMR spectrum of **1**.

Figure S15. *g*GMQC NMR spectrum of **1**.

Figure S16. *g*GMBC NMR spectrum of **1**.

Figure S17. ^{13}C NMR spectrum of **1**

Figure S18. HRESI-MS spectrum of **1**.

Figure S19. Chromatogram of **1**.

Figure S20. ^{1}H NMR spectrum of **2**.

Figure S21. *g*GMQC NMR spectrum of **2**.

Figure S22. *g*GMBC NMR spectrum of **2**.

Figure S23. ^{13}C NMR spectrum of **2**

Figure S24. HRESI-MS spectrum of **2**.

Figure S25. Chromatogram of **2**.

Figure S26. ^{1}H NMR spectrum of **3**.

Figure S27. *g*GMQC NMR spectrum of **3**.

Figure S28. *g*GMBC NMR spectrum of **3**.

Figure S29. ^{13}C NMR spectrum of **3**

Figure S30. HRESI-MS spectrum of **3**.

Figure S31. Chromatogram of **3**.

Figure S32. ^{1}H NMR spectrum of **4**.

Figure S33. *g*GMQC NMR spectrum of **4**.

Figure S34. *g*GMBC NMR spectrum of **4**.

Figure S35. ^{13}C NMR spectrum of **4**

Figure S36. HRESI-MS spectrum of **4**.

Figure S37. Chromatogram of **4**.

This is an open-access article distributed under the terms of the Creative Commons Attribution Licence.

Figure S38. ^{1}H NMR spectrum of **7**.

Figure S39. *g*GMQC NMR spectrum of **7**.

Figure S40. *g*GMBC NMR spectrum of **7**.

Figure S41. ^{13}C NMR spectrum of **7**

Figure S42. HRESI-MS spectrum of **7**.

Figure S43. Chromatogram of **7**.

Figure S44. Cell inhibition data obtained for HGC-27 and BT20 – Series A and B.

Figure S45. Cell inhibition data obtained for HGC-27, BT20 and CAL-27 – Series C.

Figure S46. Cell cycle phases in tumor cell lines (BT20, CAL-27, and HGC-27).

This is an open-access article distributed under the terms of the Creative Commons Attribution Licence.

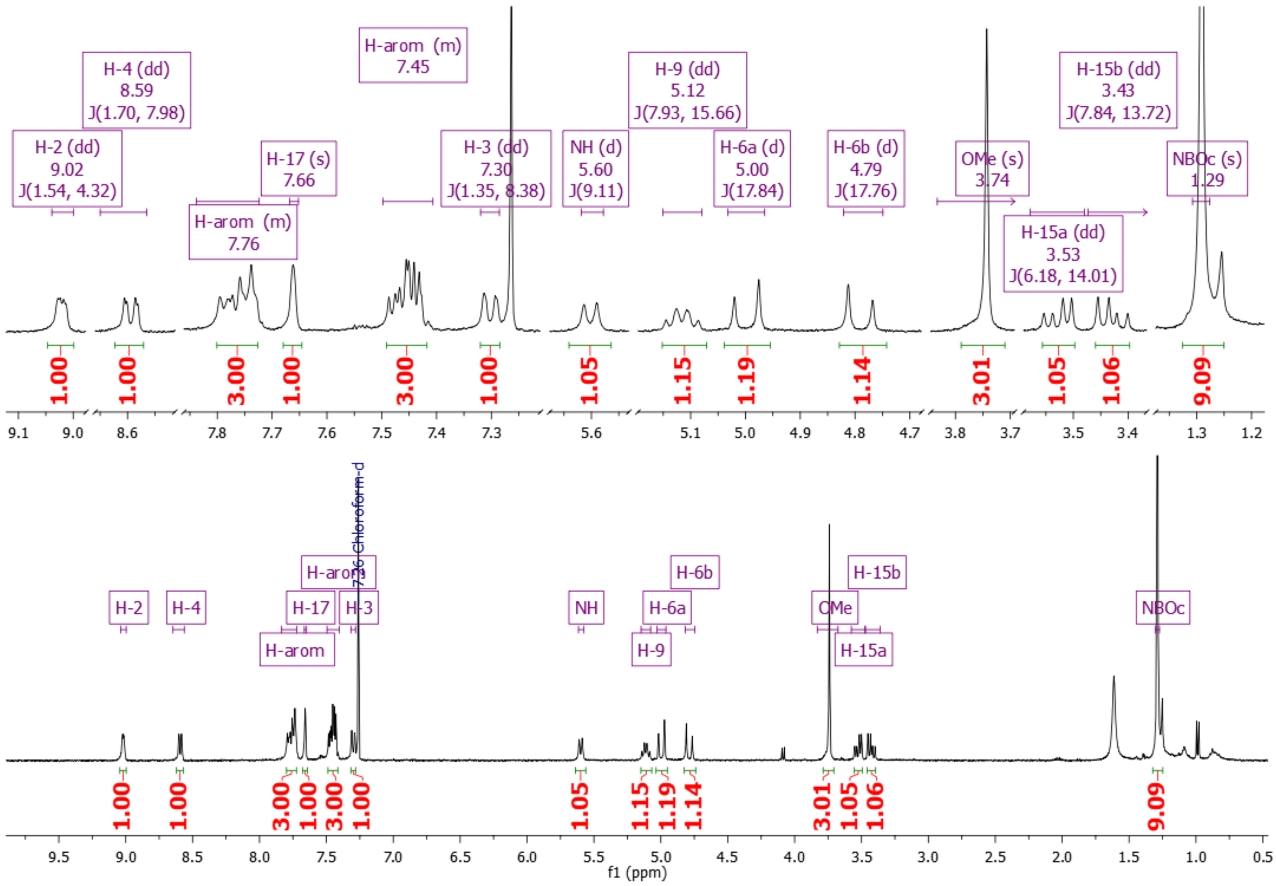

Figure S1. ^{1}H NMR spectrum (300 MHz, $CDCl_3$) of **11**.

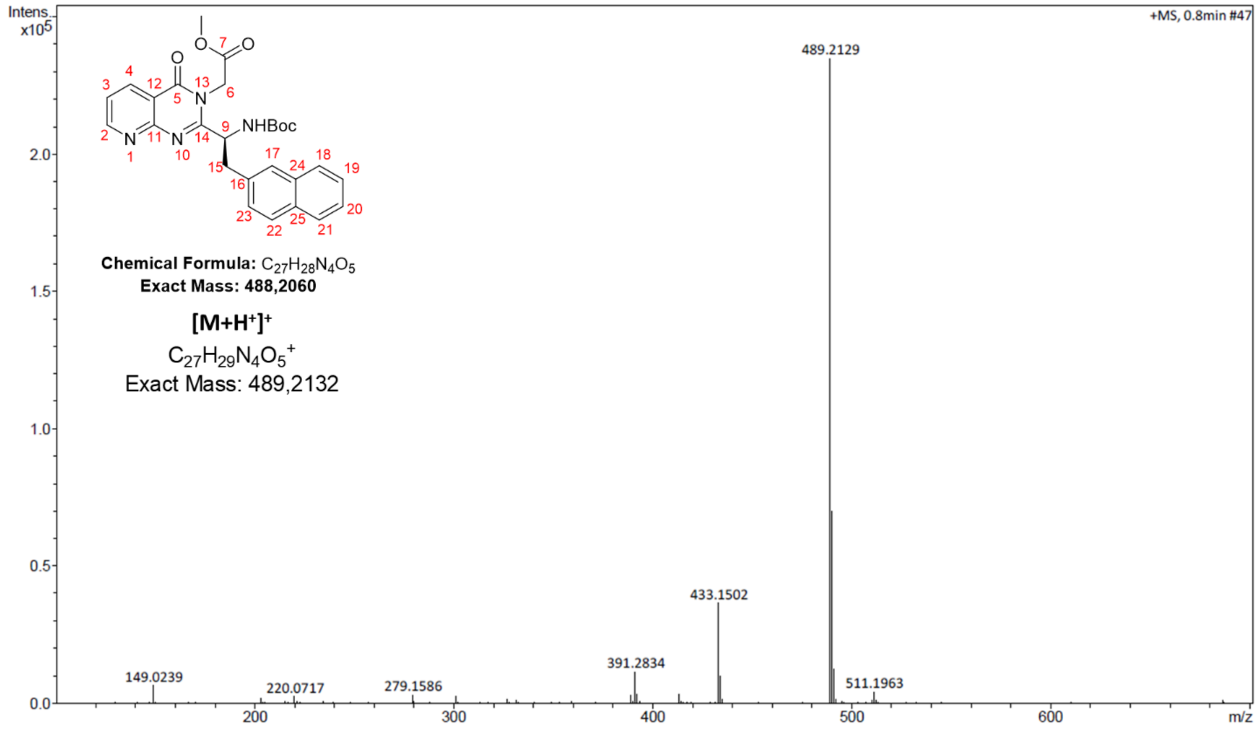

Figure S2. HRESI-MS spectrum of **11** (m/z calcd. for $C_{27}H_{28}N_4O_5^+$ $[M+H]^+$ 489.2132, found 489.2129).

This is an open-access article distributed under the terms of the Creative Commons Attribution Licence.

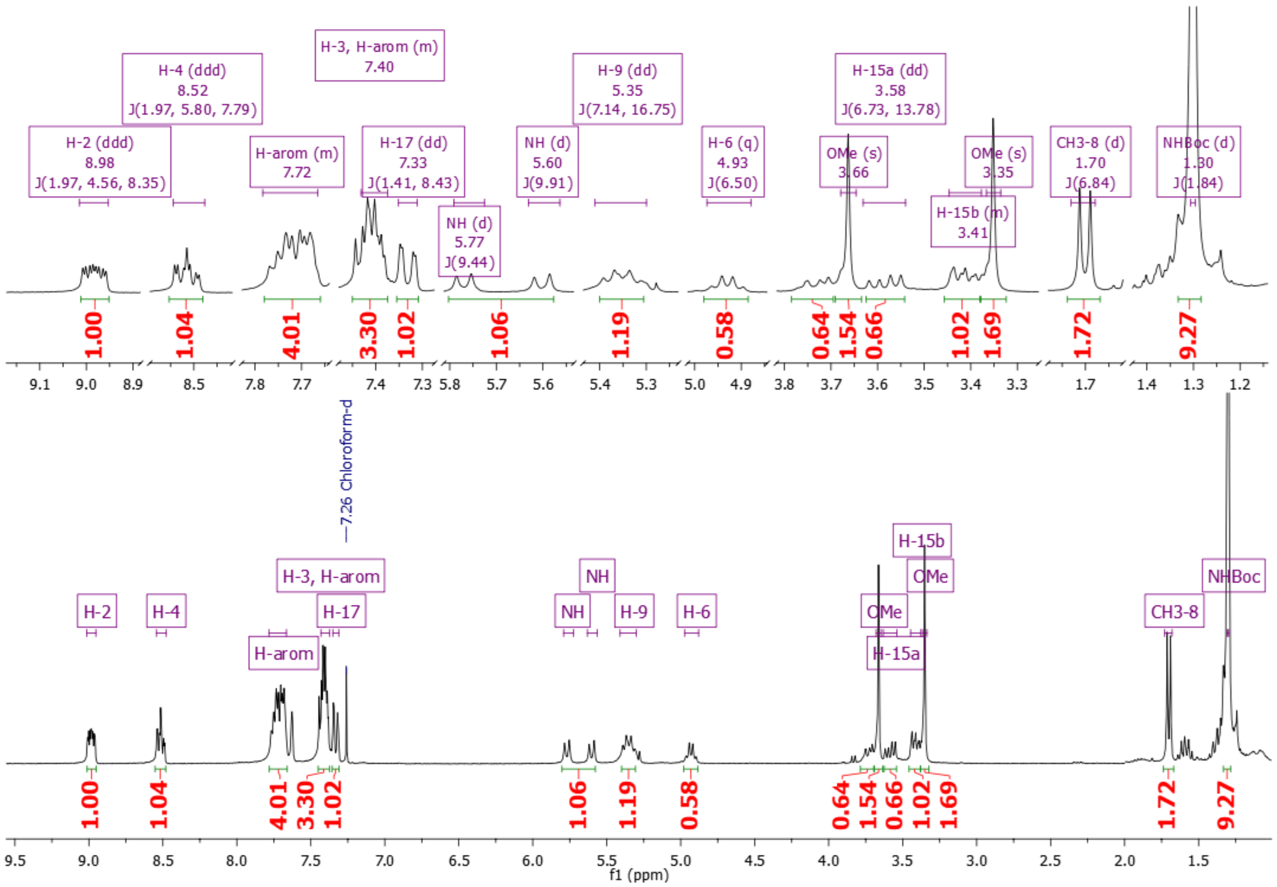

Figure S3. ^{1}H NMR spectrum (300 MHz, $CDCl_3$) of **12**.

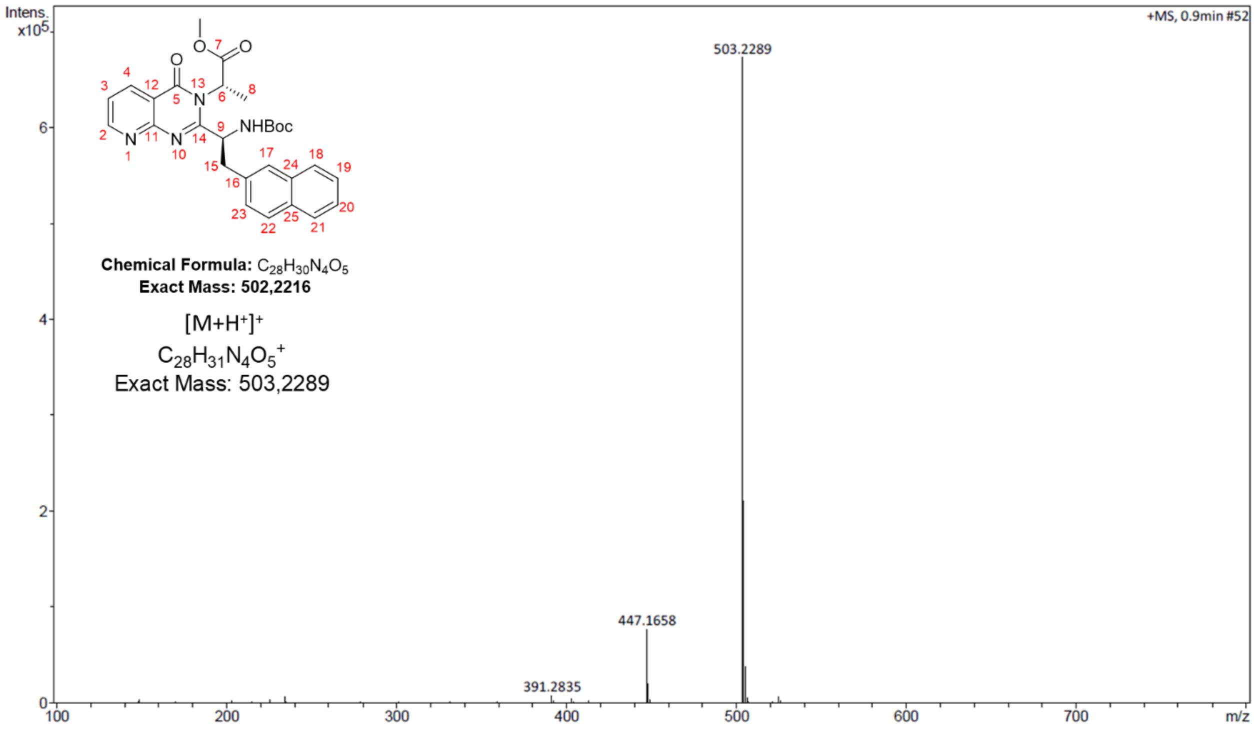

Figure S4. HRESI-MS spectrum of **12** (m/z calcd. for $C_{28}H_{31}N_4O_5^+$ $[M+H]^+$ 503.2289, found 503.2289).

This is an open-access article distributed under the terms of the Creative Commons Attribution Licence.

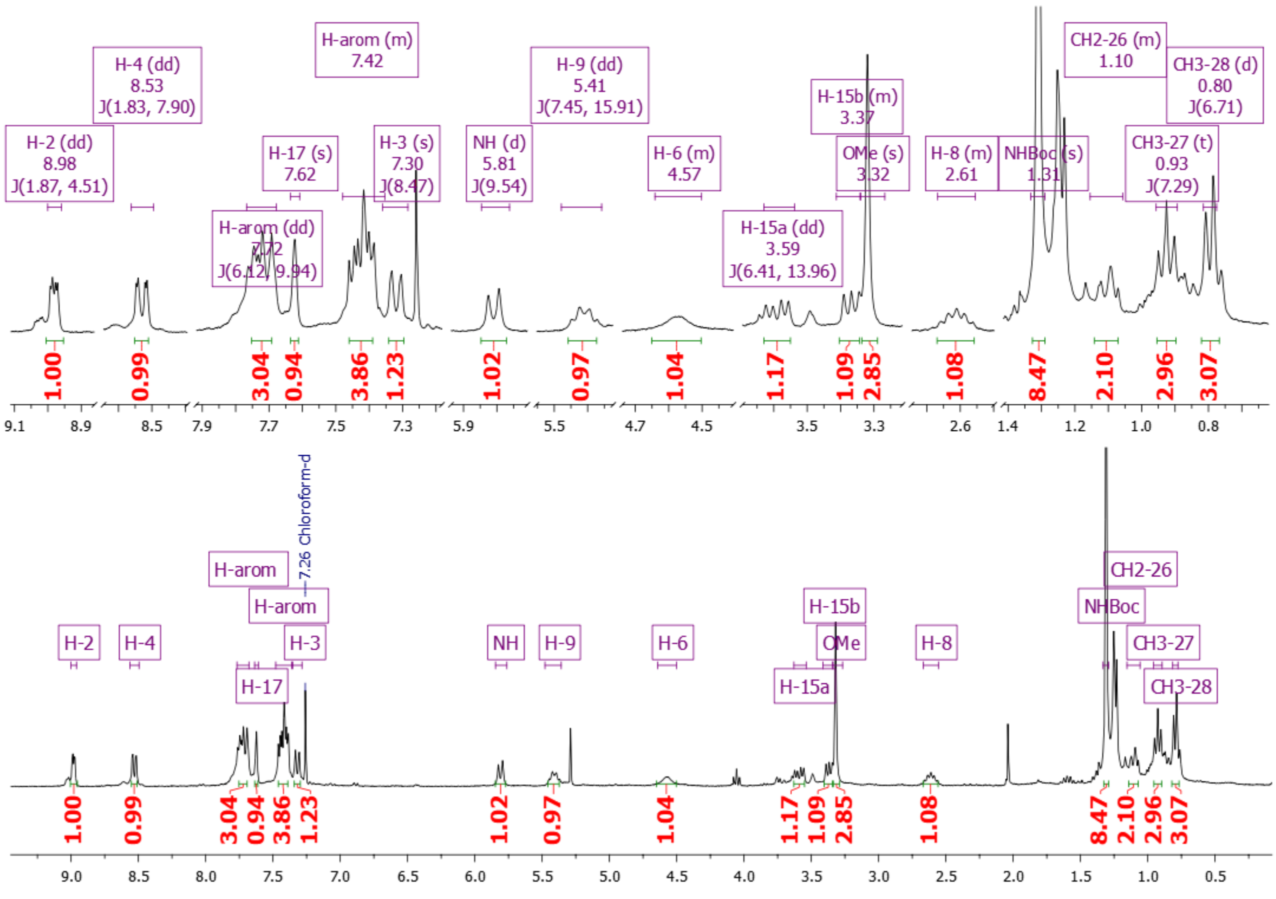

Figure S5. ^{1}H NMR spectrum (300 MHz, $CDCl_3$) of **13**.

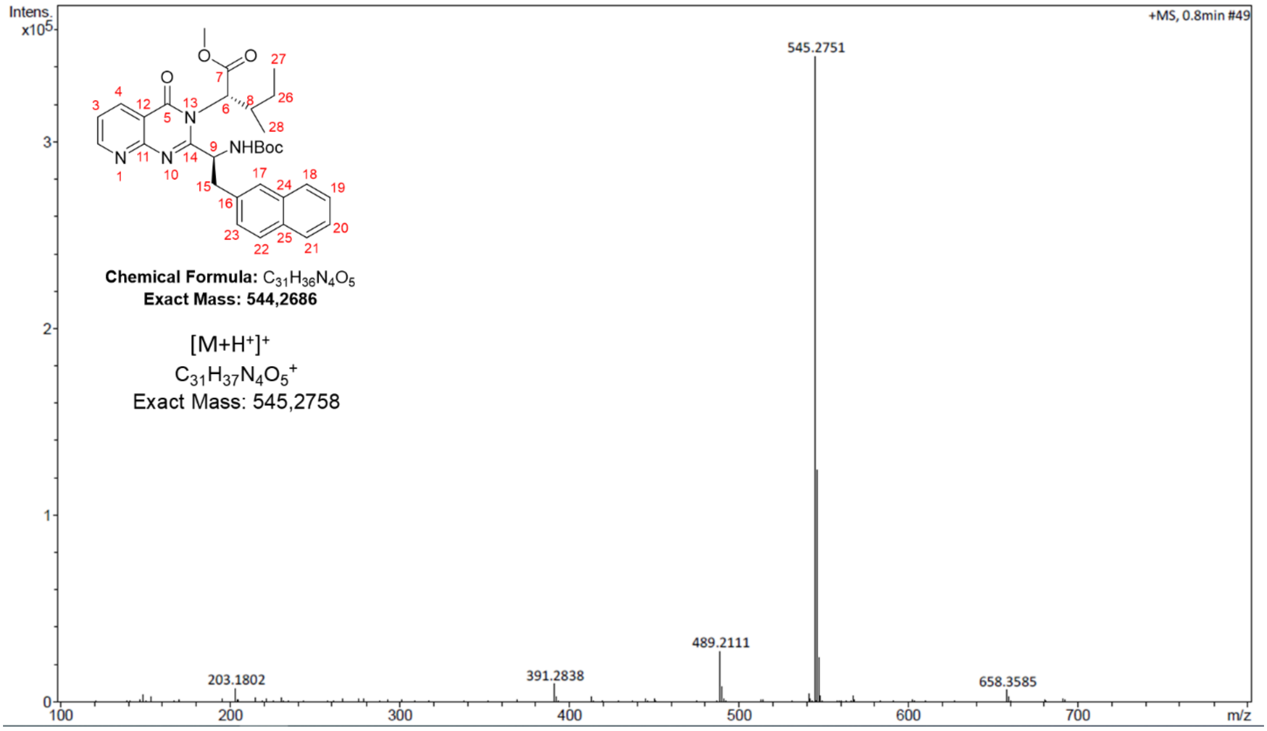

Figure S6. HRESI-MS spectrum of **13** (m/z calcd. for $C_{31}H_{37}N_4O_5^+$ $[M+H]^+$ 545.2758, found 545.2751).

This is an open-access article distributed under the terms of the Creative Commons Attribution Licence.

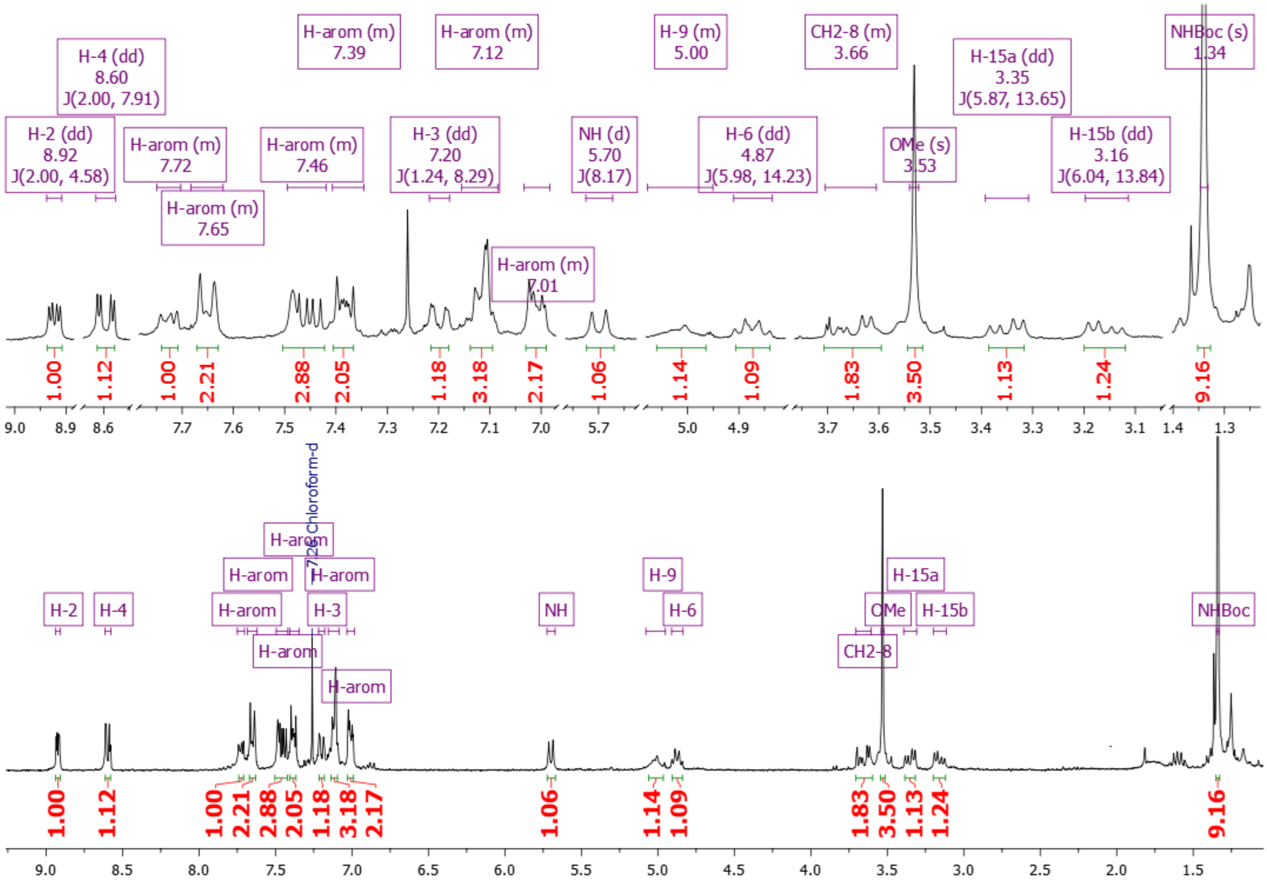

Figure S7. ^{1}H NMR spectrum (300 MHz, $CDCl_3$) of **14**.

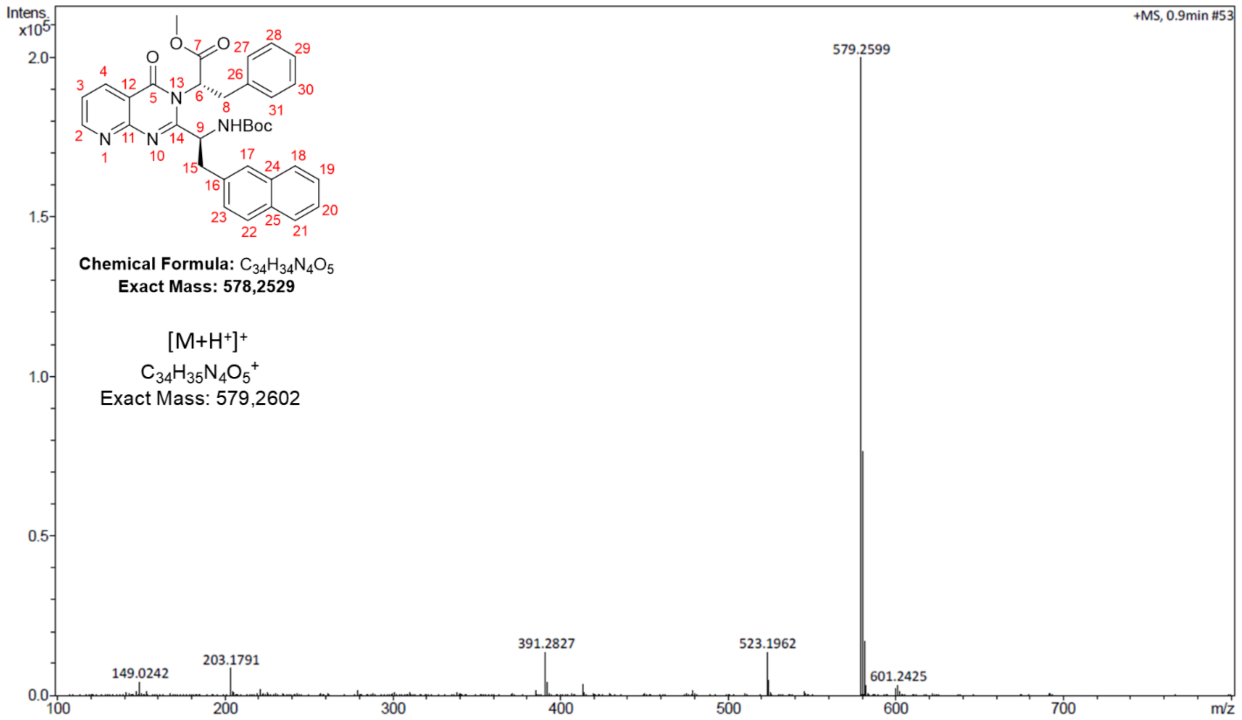

Figure S8. HRESI-MS spectrum of **14** (m/z calcd. for $C_{34}H_{35}N_4O_5^+$ $[M+H]^+$ 579.2602, found 579.2599).

This is an open-access article distributed under the terms of the Creative Commons Attribution Licence.

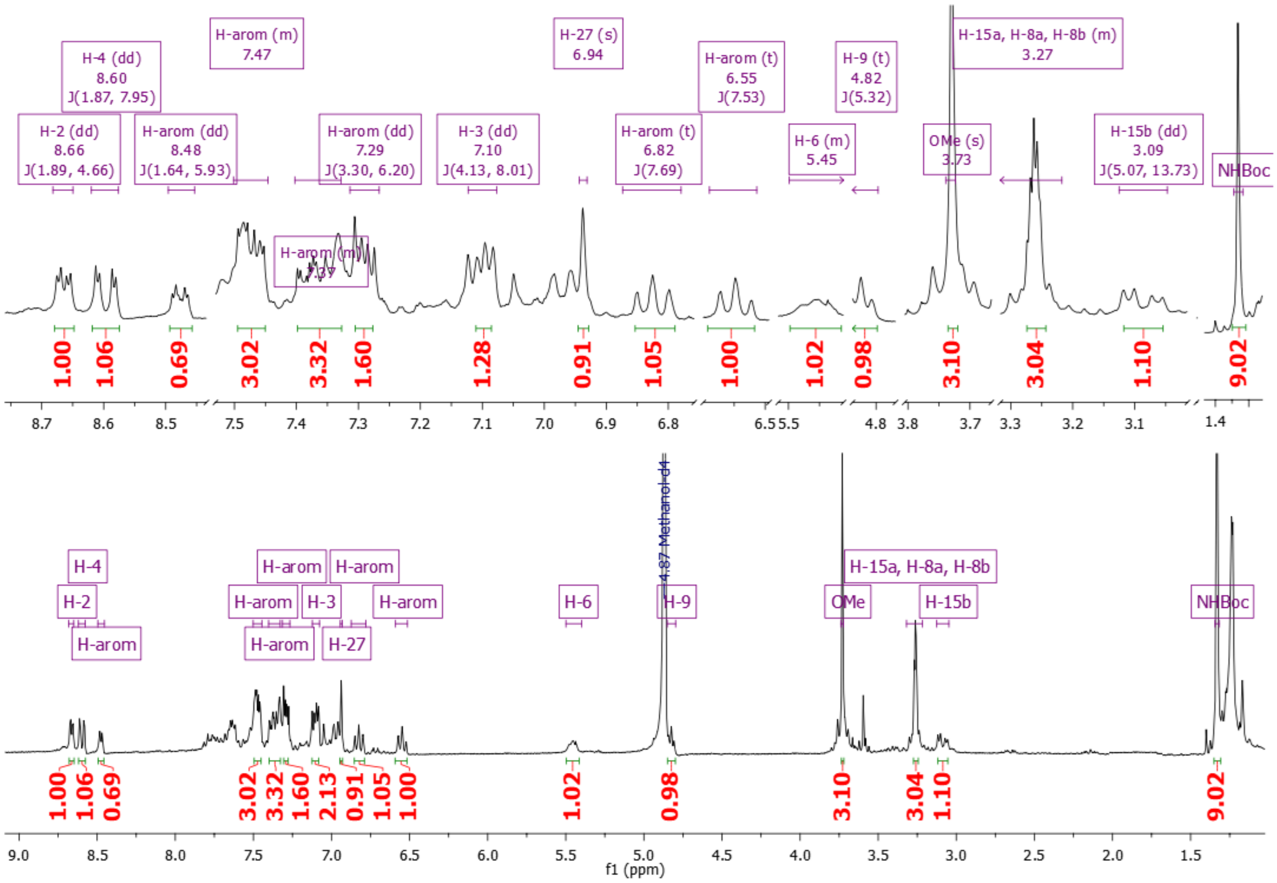

Figure S9. ^{1}H NMR spectrum (300 MHz, $CDCl_3$) of **15**.

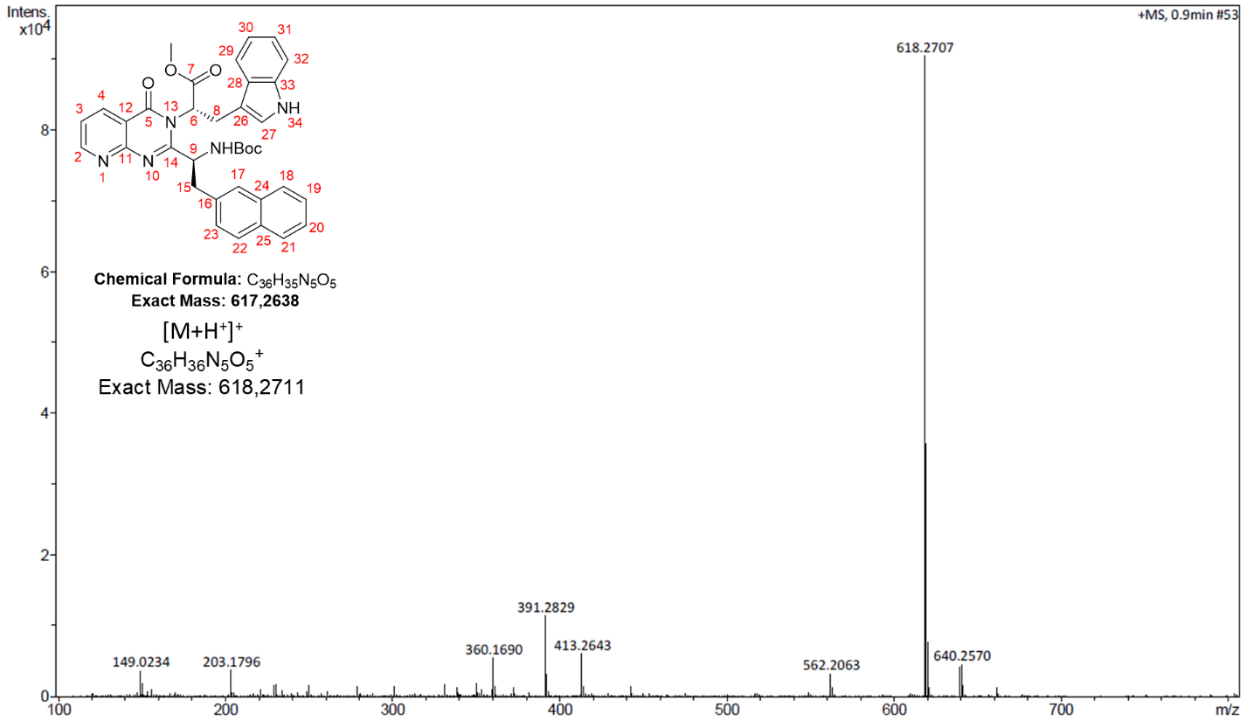

Figure S10. HRESI-MS spectrum of **15** (m/z calcd. for $C_{36}H_{36}N_5O_5^+$ $[M+H]^+$ 618.2711, found 618.2707).

This is an open-access article distributed under the terms of the Creative Commons Attribution Licence.

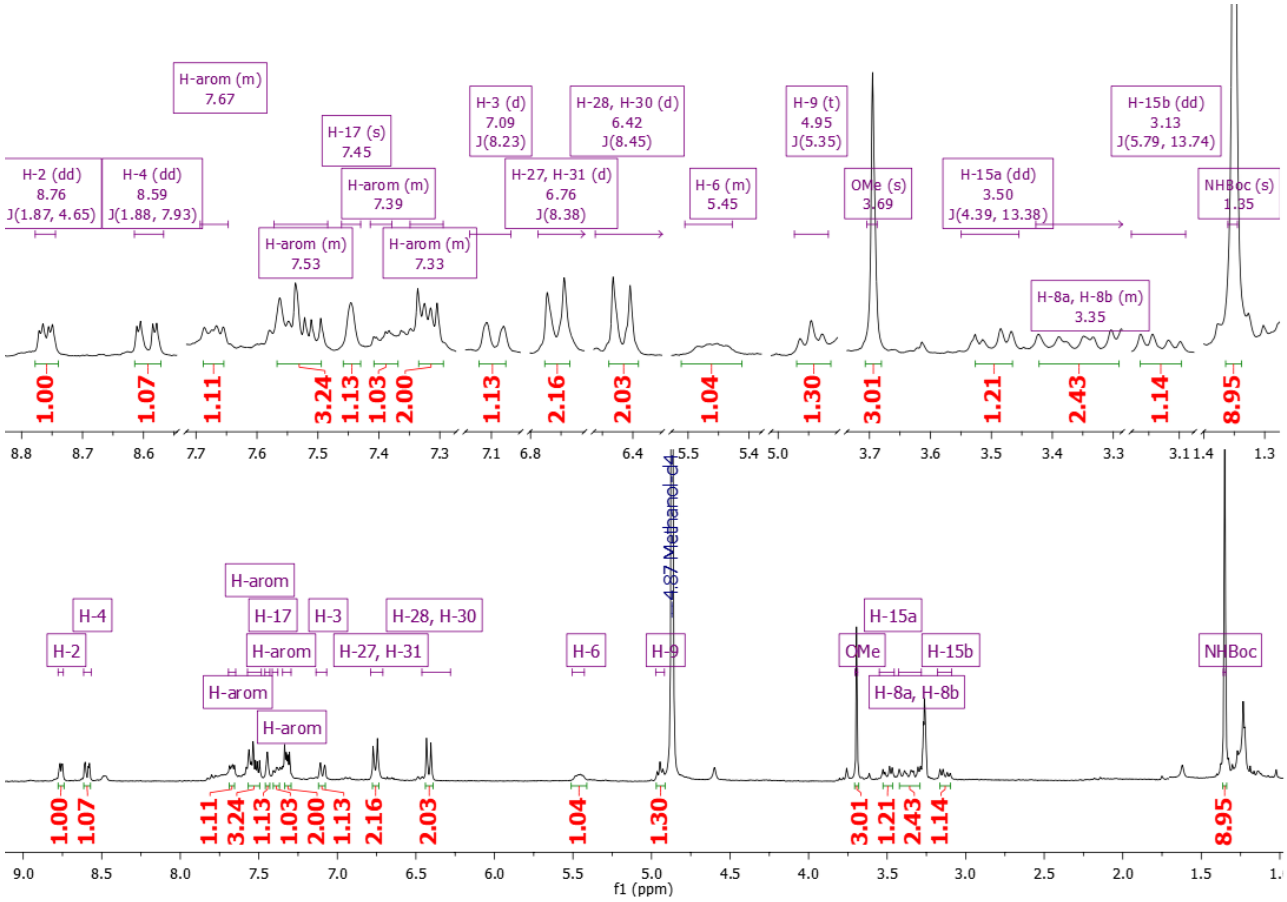

Figure S11. ^{1}H NMR spectrum (300 MHz, $CDCl_3$) of **16**.

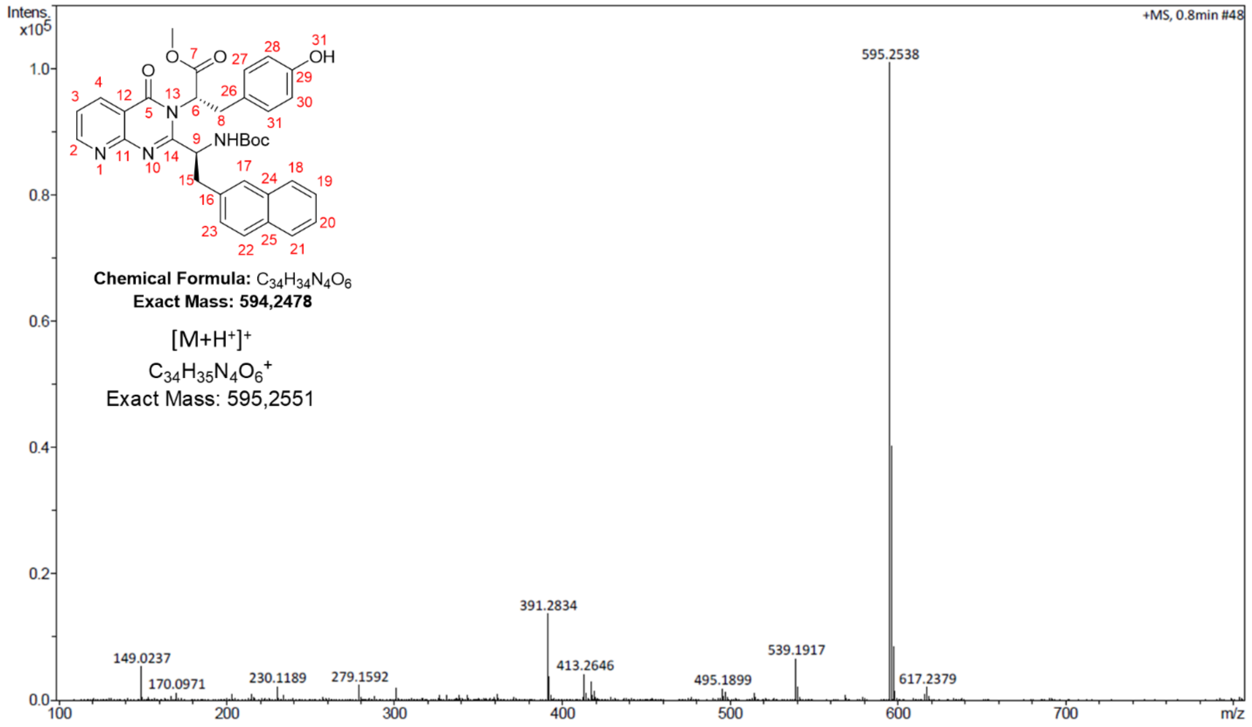

Figure S12. HRESI-MS spectrum of **16** (m/z calcd. for $C_{34}H_{35}N_4O_6^+$ $[M+H]^+$ 595.2551, found 595.2538).

This is an open-access article distributed under the terms of the Creative Commons Attribution Licence.

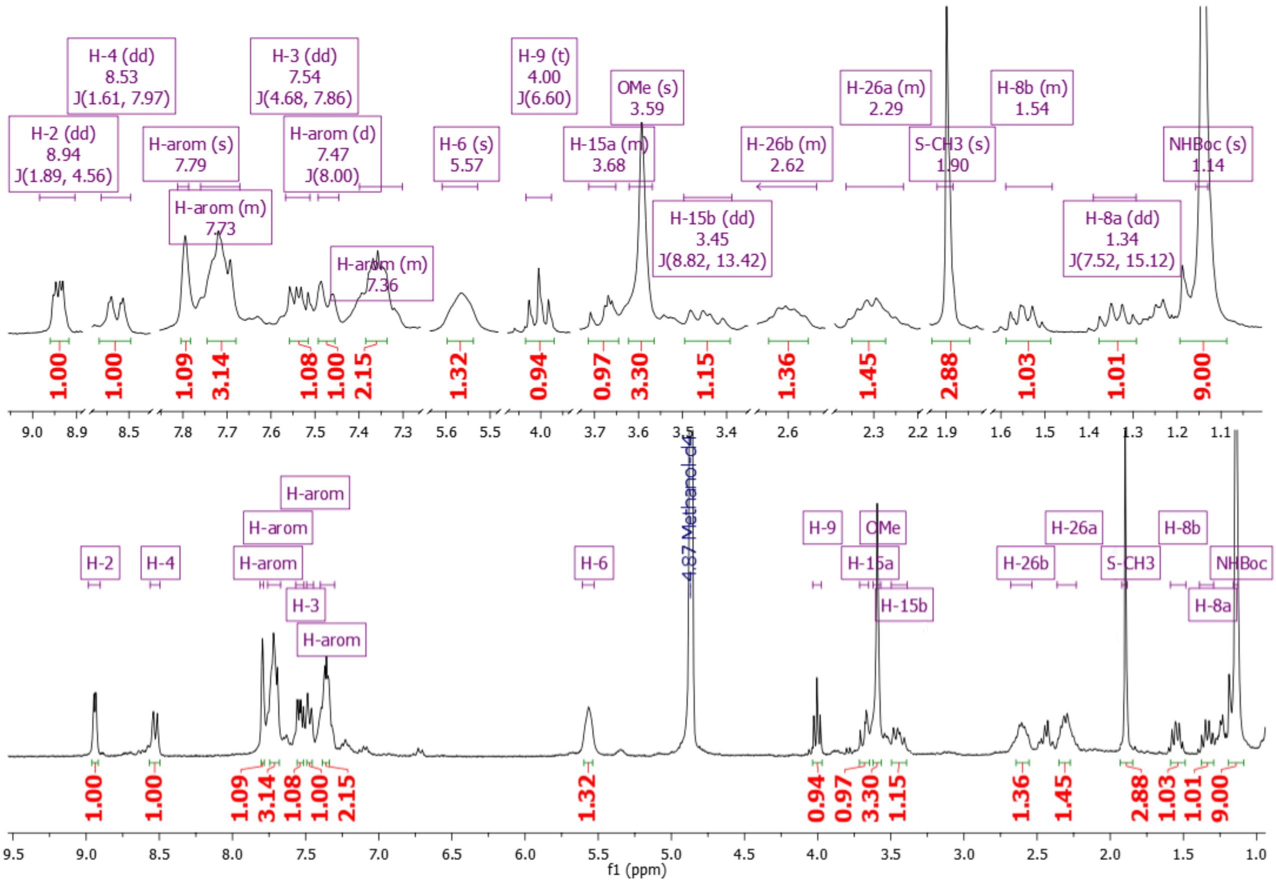

Figure S13. ^{1}H NMR spectrum (300 MHz, $CDCl_3$) of **17**.

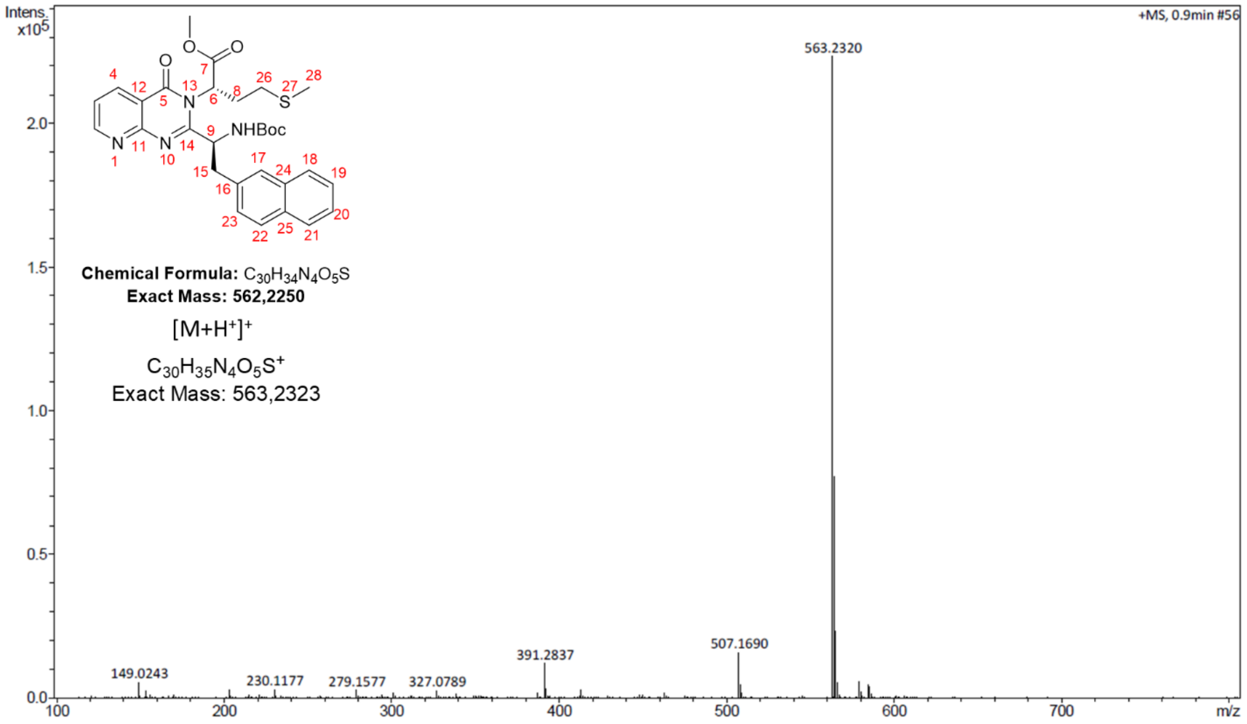

Figure S14. HRESI-MS spectrum of **17** (m/z calcd. for $C_{30}H_{35}N_4O_5S^+$ $[M+H]^+$ 563.2323, found 563.2320).

This is an open-access article distributed under the terms of the Creative Commons Attribution Licence.

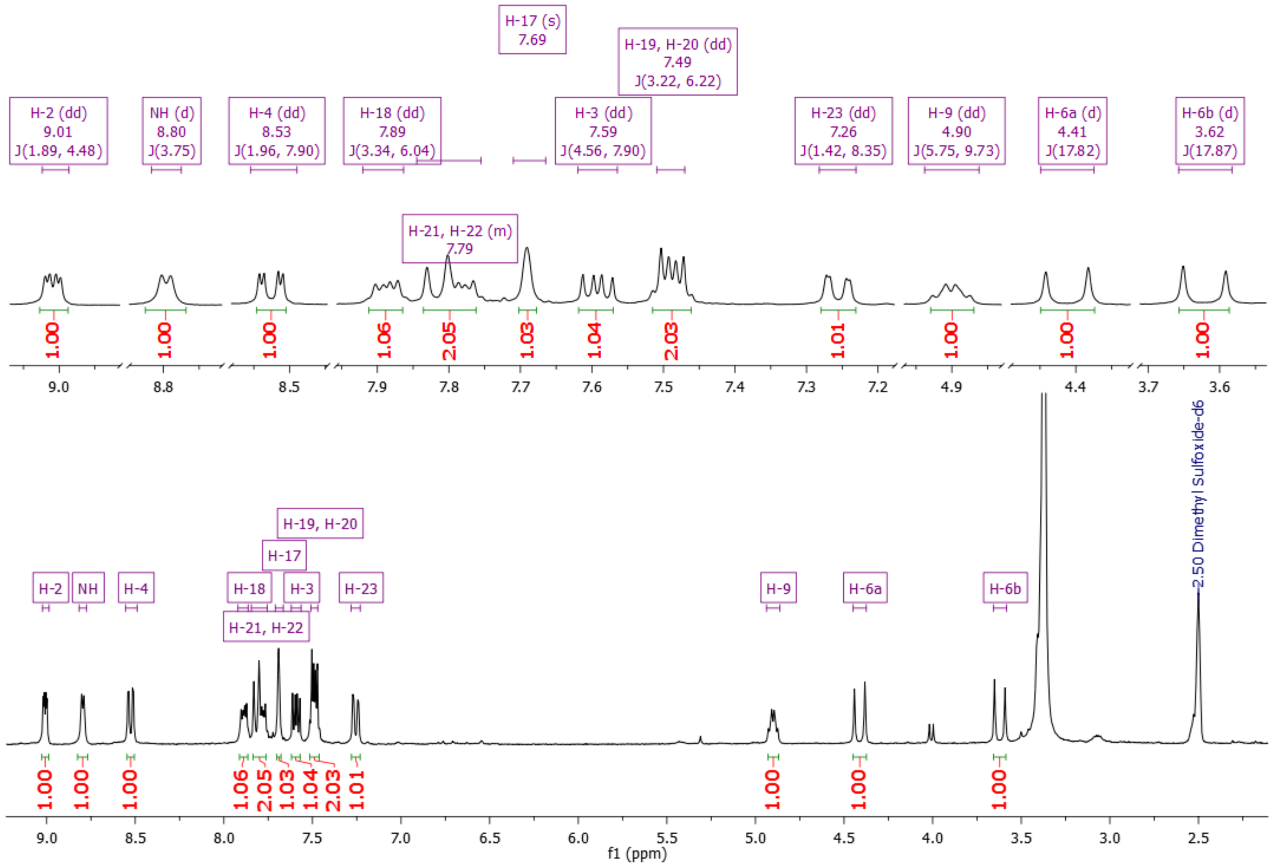

Figure S15. ^{1}H NMR spectrum (400 MHz, DMSO-d$_6$) of **1**.

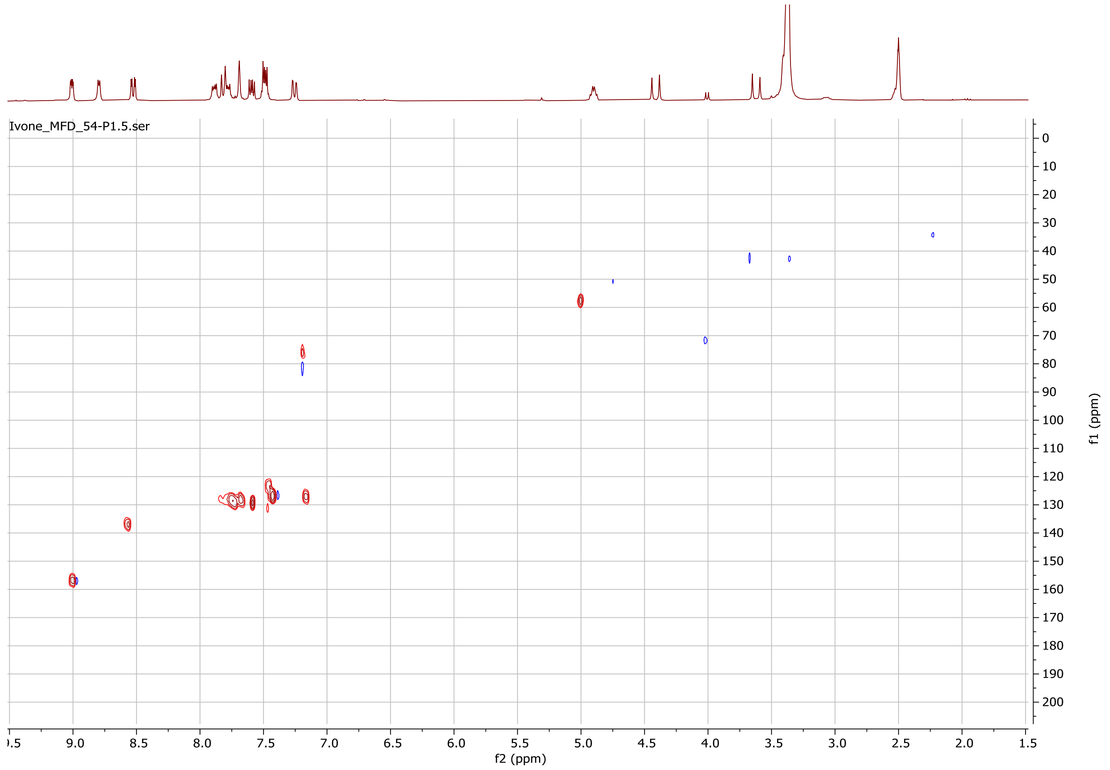

Figure S16. *g*GMQC NMR spectrum (150 MHz, $CDCl_3$) of **1**.

This is an open-access article distributed under the terms of the Creative Commons Attribution Licence.

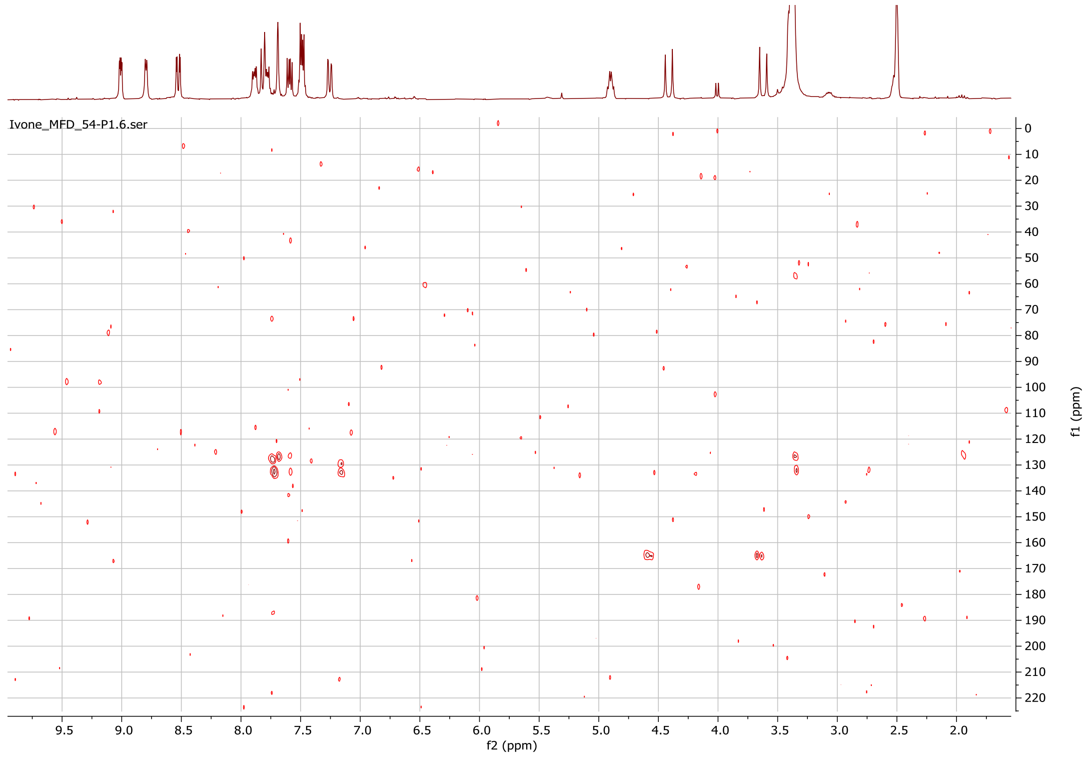

Figure S17. *g*GMBC spectrum (150 MHz, $CDCl_3$) of **1**.

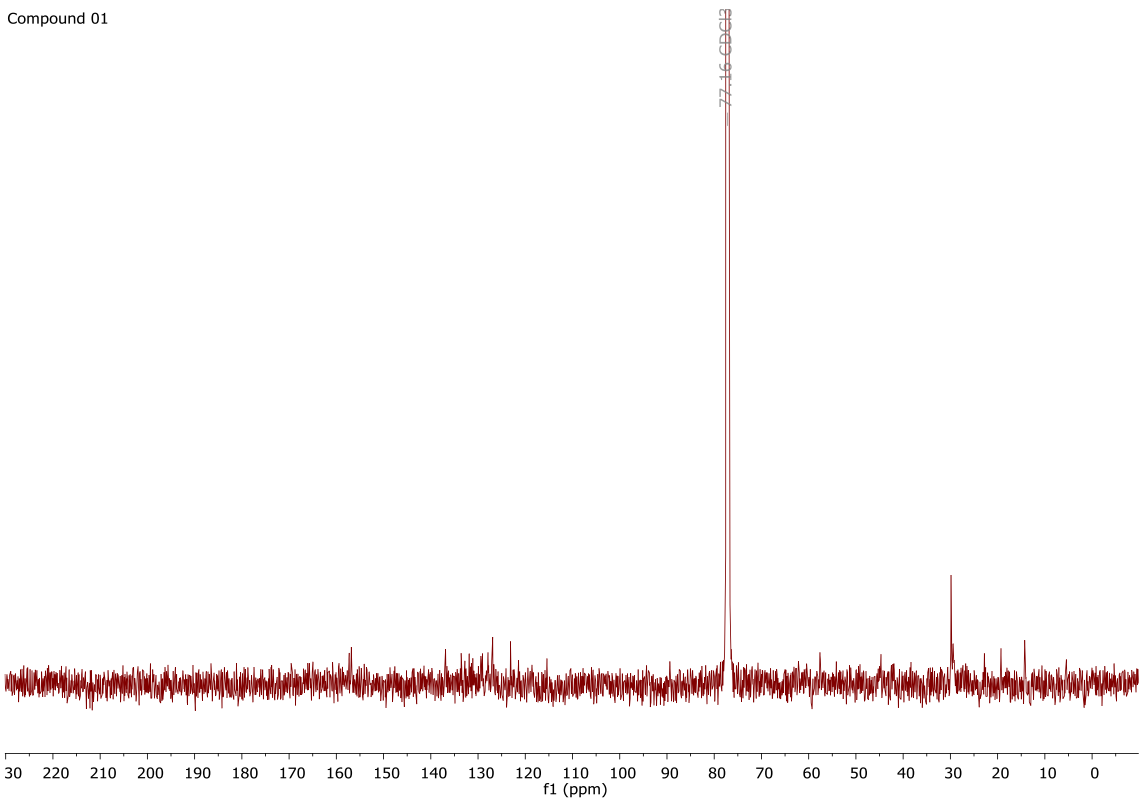

Figure S18. ^{13}C NMR spectrum (150 MHz, $CDCl_3$) of **1**.

This is an open-access article distributed under the terms of the Creative Commons Attribution Licence.

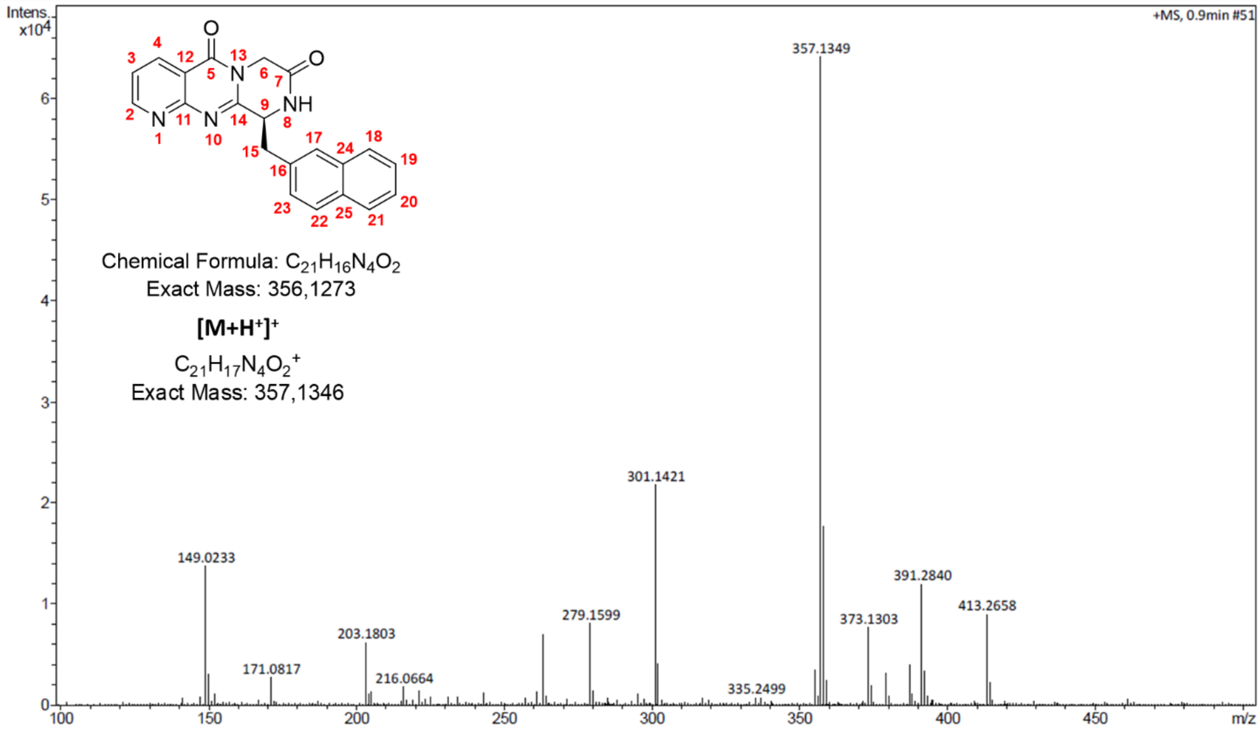

Figure S19. HRESI-MS spectrum of **1** (m/z calcd. for $C_{21}H_{17}N_4O_2^+$ $[M+H]^+$ 357.1346, found 357.1349).

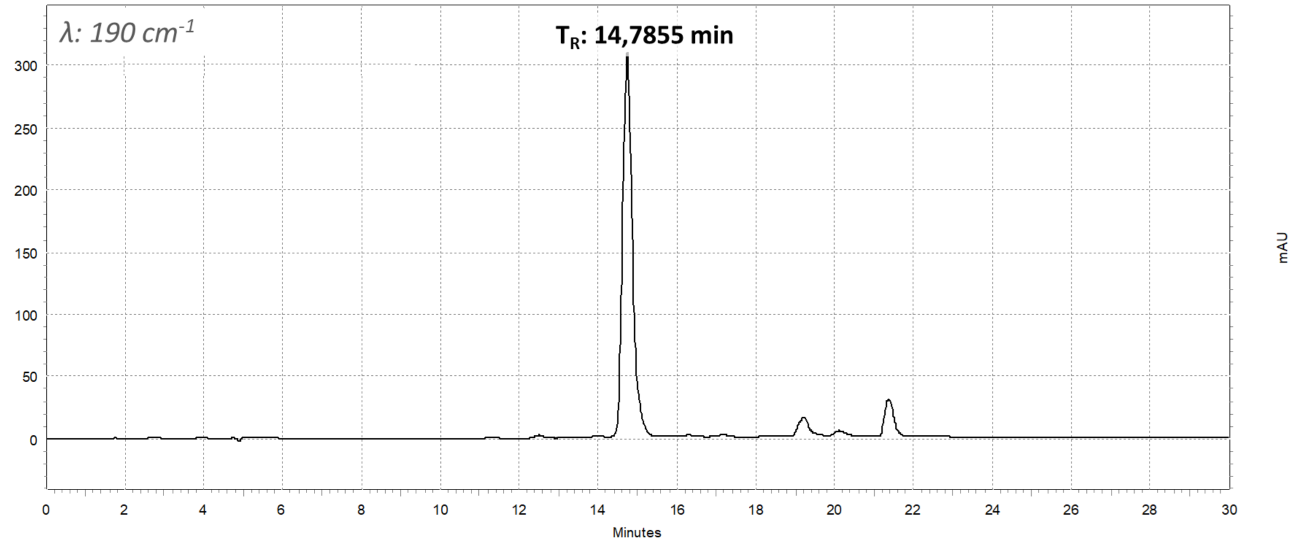

Figure S20. Chromatogram of **1** after purification by HPLC (RT = 14.79 min).

This is an open-access article distributed under the terms of the Creative Commons Attribution Licence.

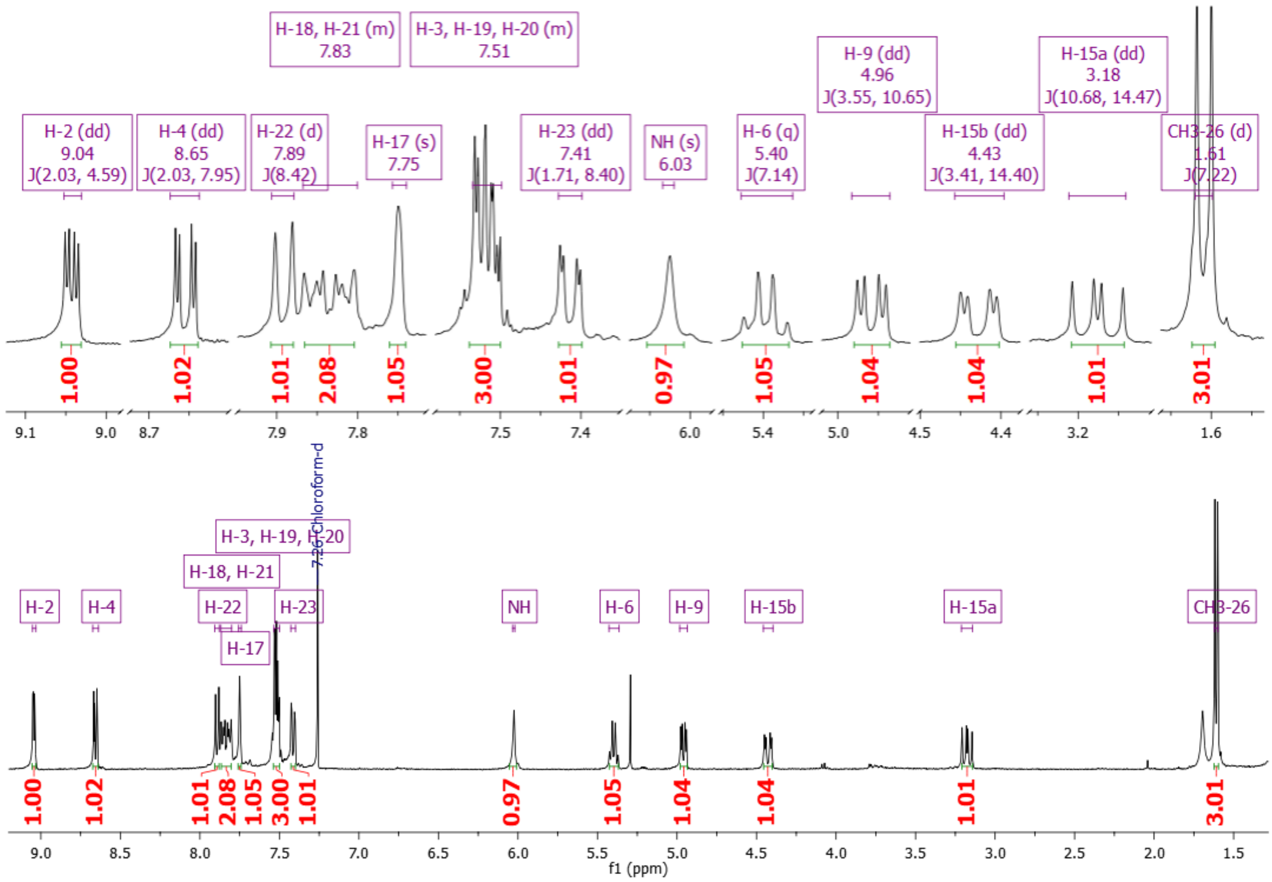

Figure S21. ^{1}H NMR spectrum (400 MHz, $CDCl_3$) of **2**.

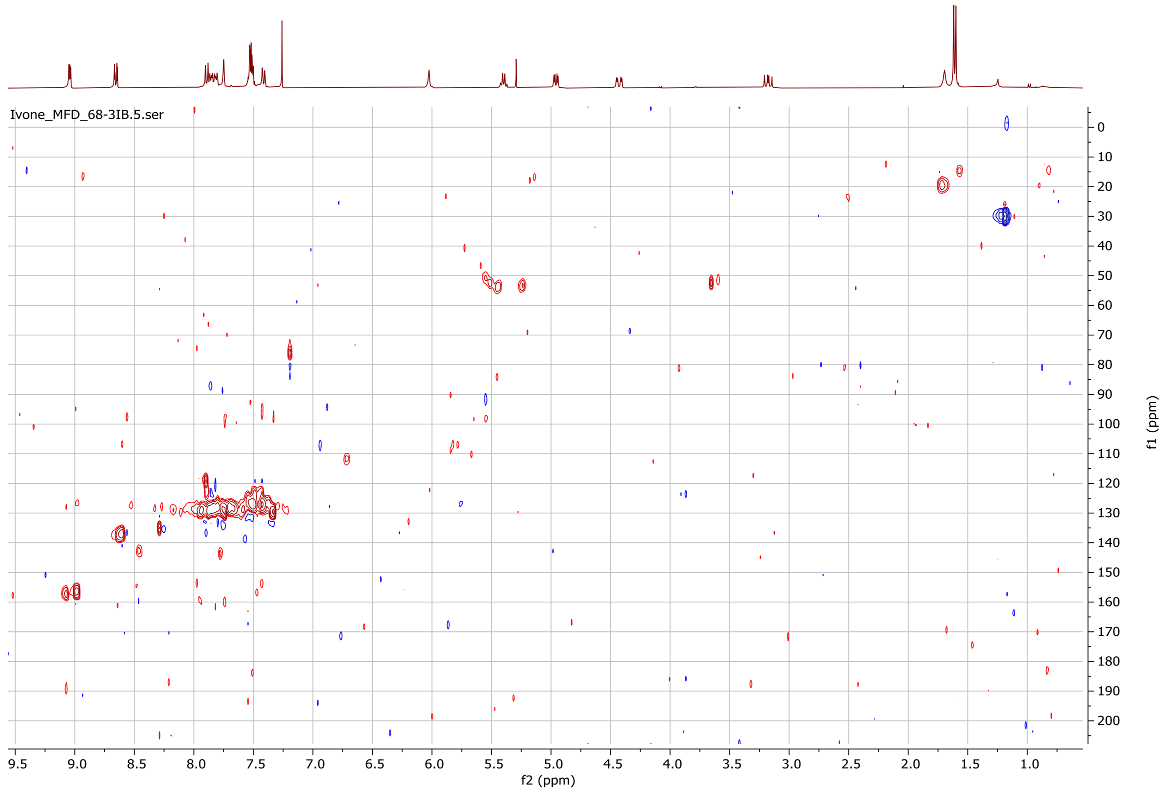

Figure S22. *g*GMQC NMR spectrum (150 MHz, $CDCl_3$) of **2**.

This is an open-access article distributed under the terms of the Creative Commons Attribution Licence.

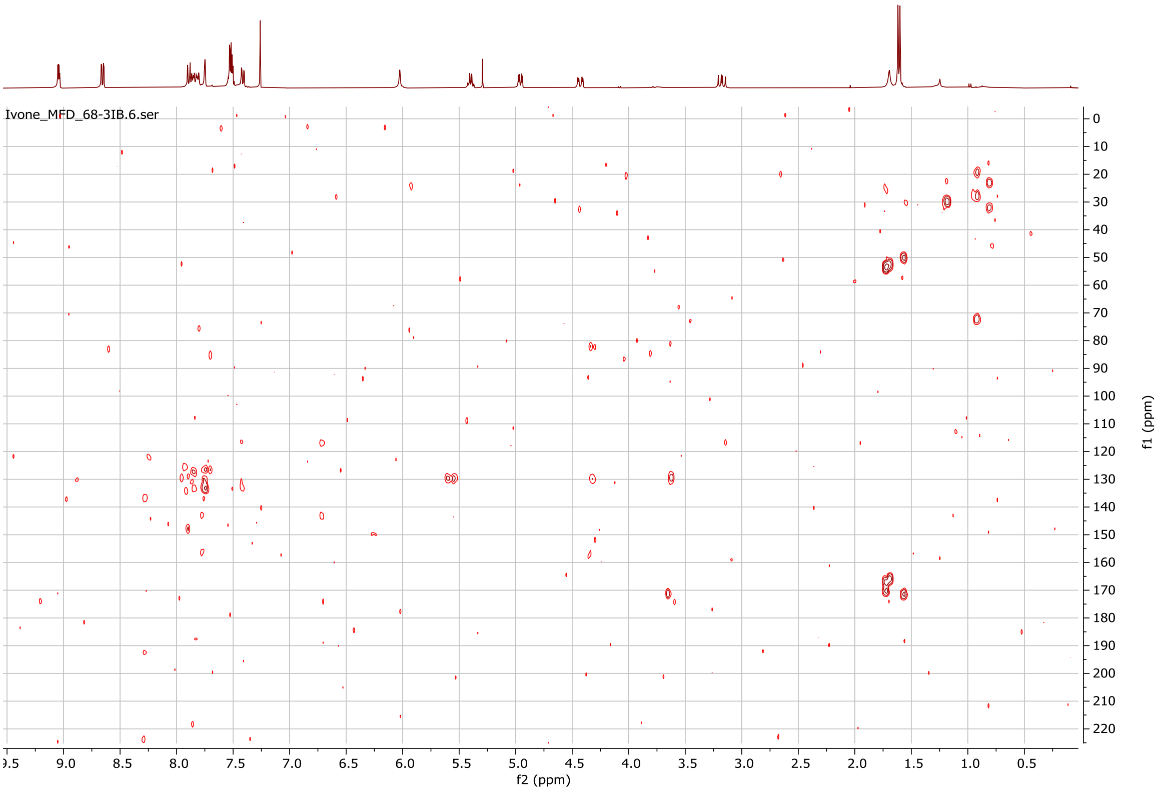

Figure S23. *g*GMBC spectrum (150 MHz, $CDCl_3$) of **2**.

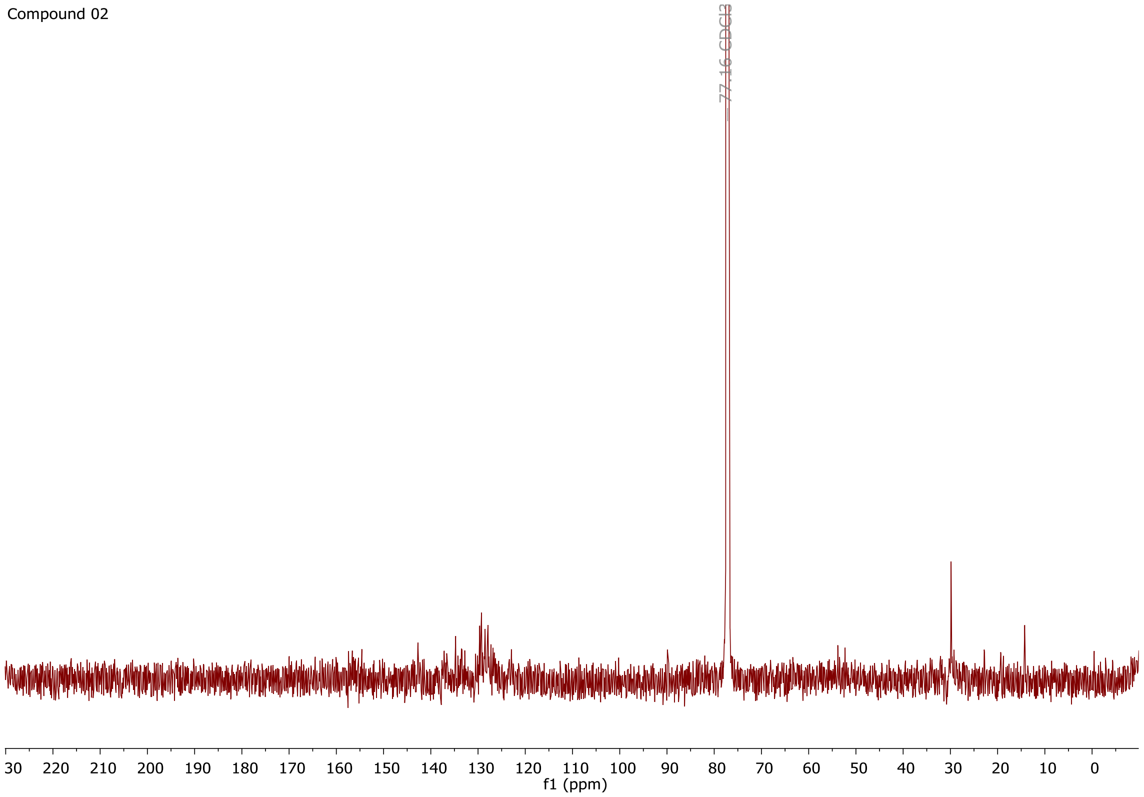

Figure S24. ^{13}C NMR spectrum (150 MHz, $CDCl_3$) of **2**.

This is an open-access article distributed under the terms of the Creative Commons Attribution Licence.

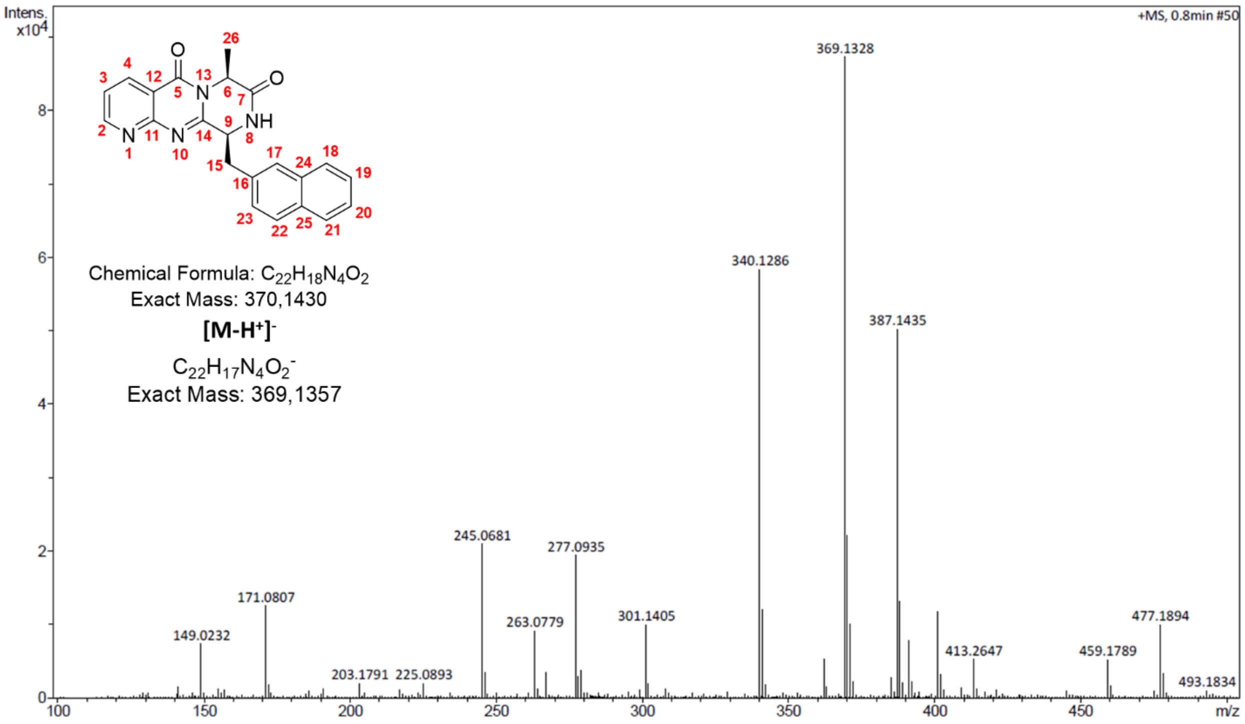

Figure S25. HRESI-MS spectrum of **2** (m/z calcd. for $C_{22}H_{17}N_4O_2^-$ $[M-H]^+$ 369.1357, found 369.1328).

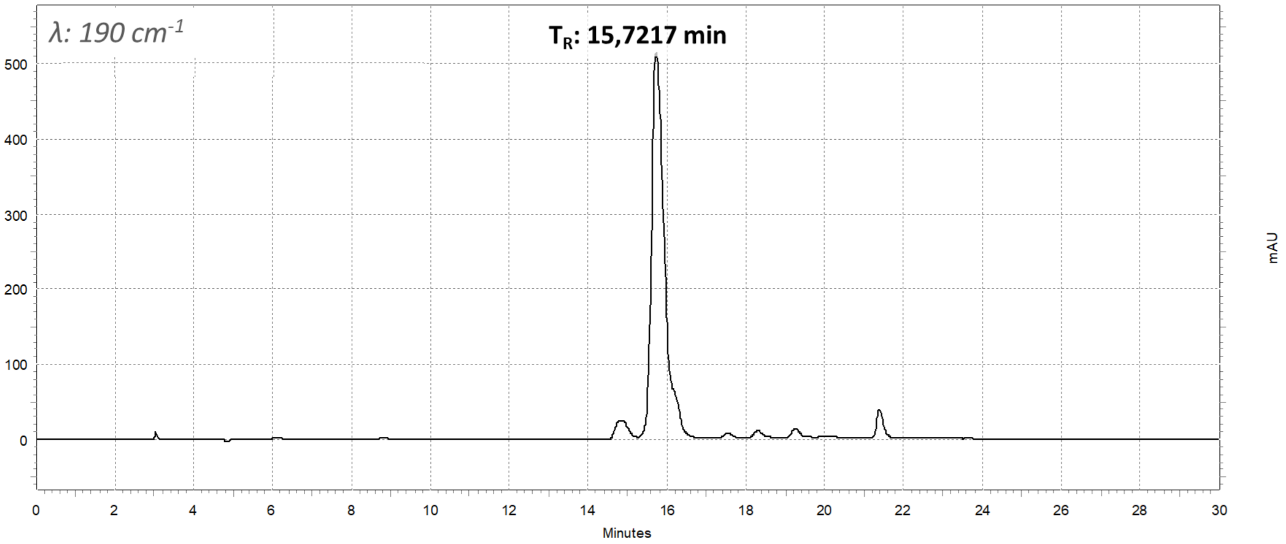

Figure S26. Chromatogram of **2** after purification by HPLC (RT = 15.72 min).

This is an open-access article distributed under the terms of the Creative Commons Attribution Licence.

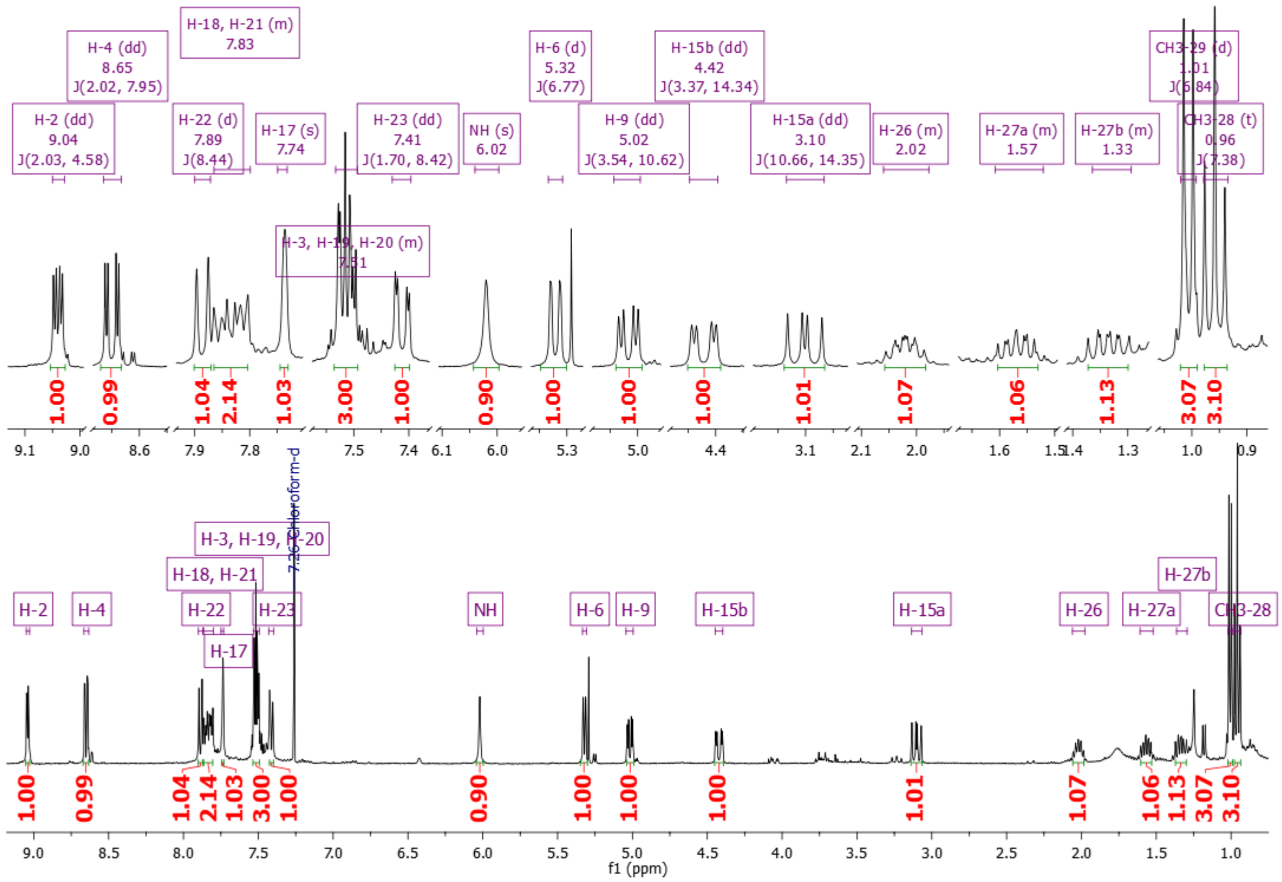

Figure S27. ^{1}H NMR spectrum (400 MHz, $CDCl_3$) of **3**.

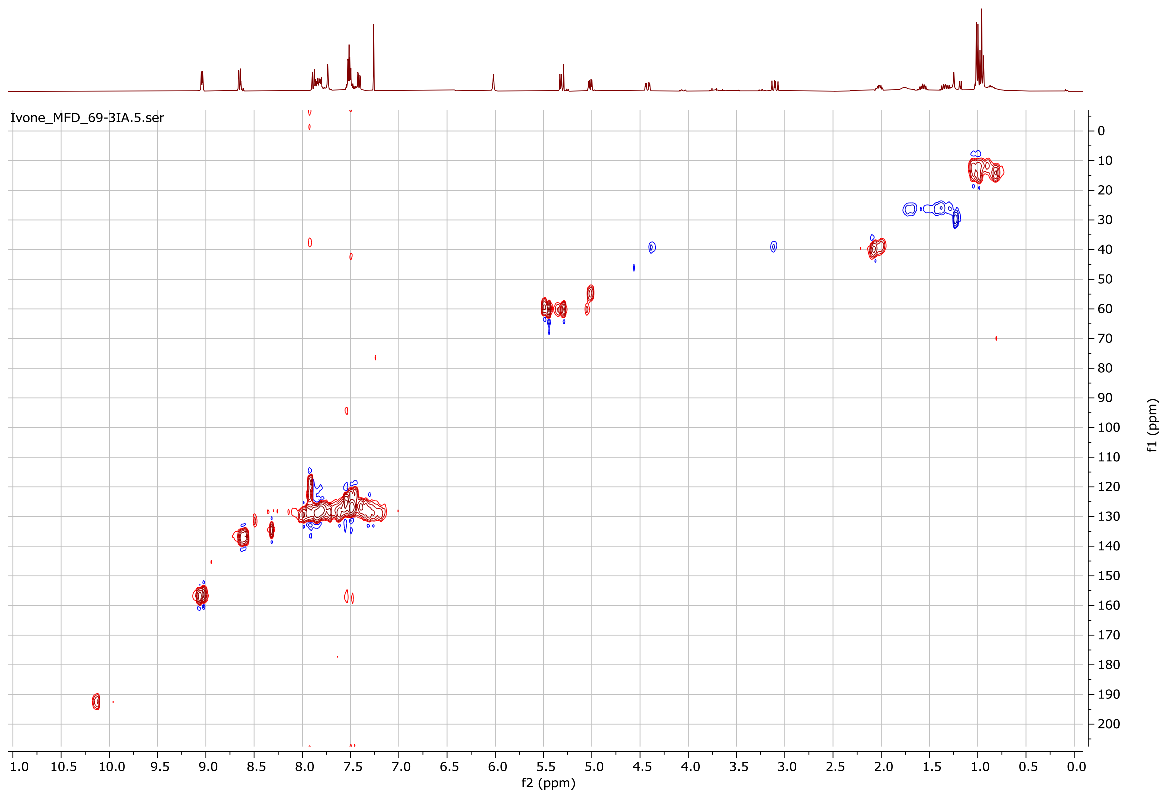

Figure S28. *g*GMQC NMR spectrum (150 MHz, $CDCl_3$) of **3**.

This is an open-access article distributed under the terms of the Creative Commons Attribution Licence.

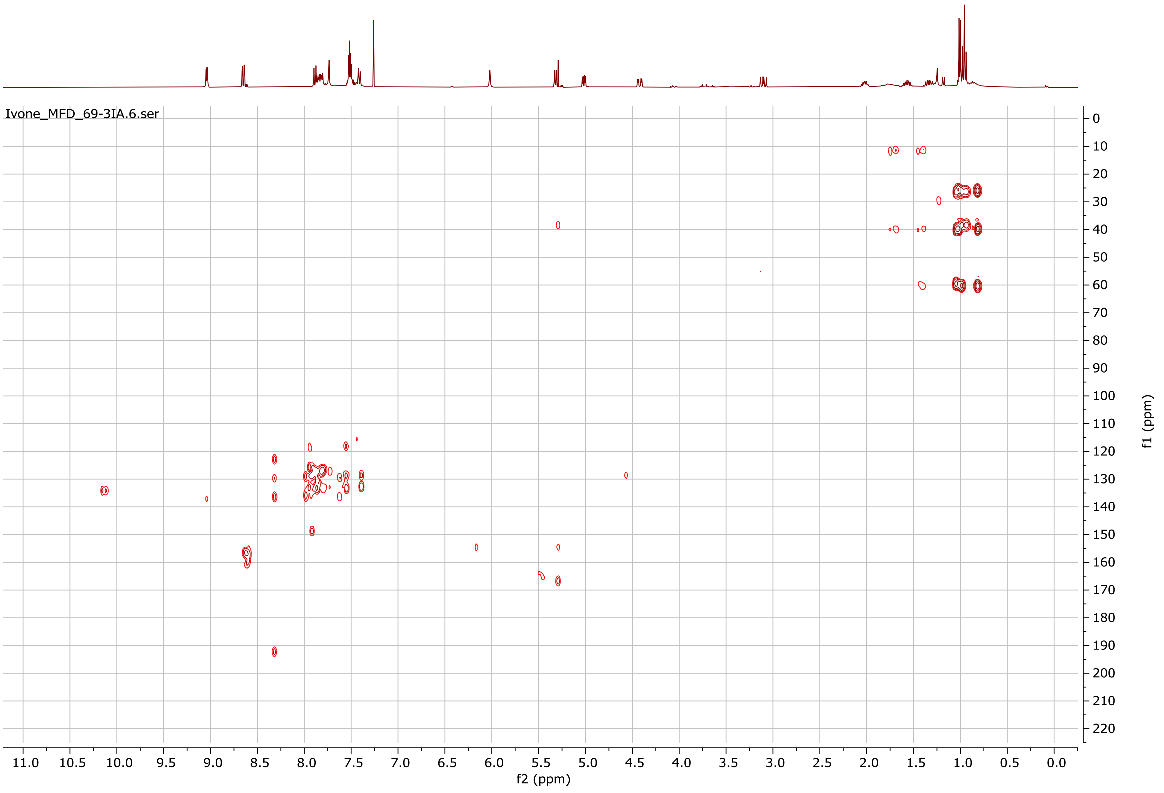

Figure S29. *g*GMBC spectrum (150 MHz, $CDCl_3$) of **3**.

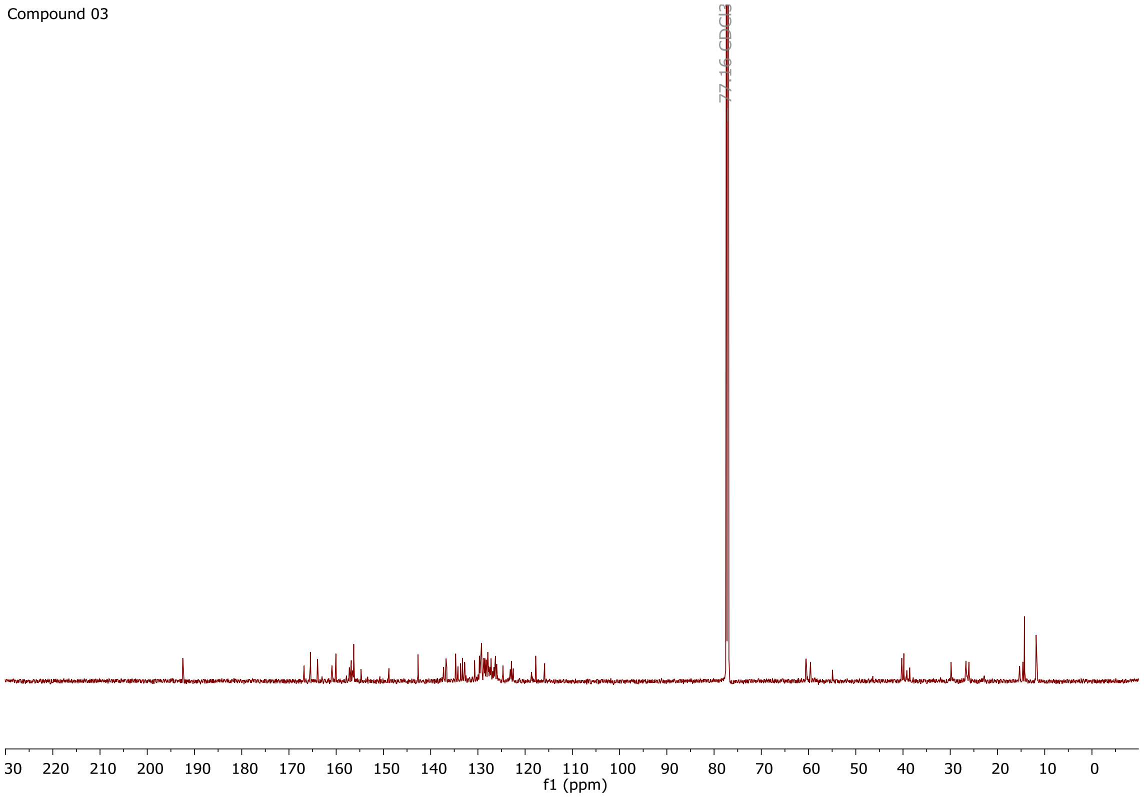

Figure S30. ^{13}C NMR spectrum (150 MHz, $CDCl_3$) of **3**.

This is an open-access article distributed under the terms of the Creative Commons Attribution Licence.

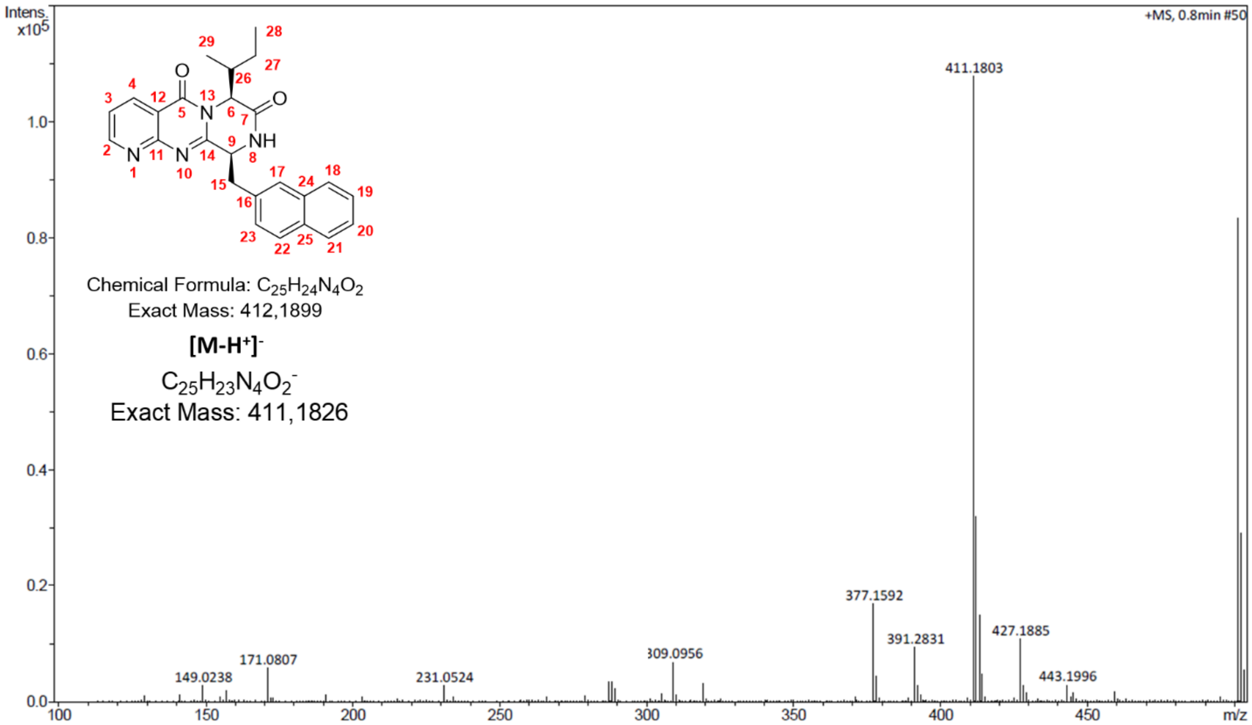

Figure S31. HRESI-MS spectrum of **3** (m/z calcd. for $C_{25}H_{23}N_4O_2^-$ $[M-H]^+$ 411.1826, found 411.1803).

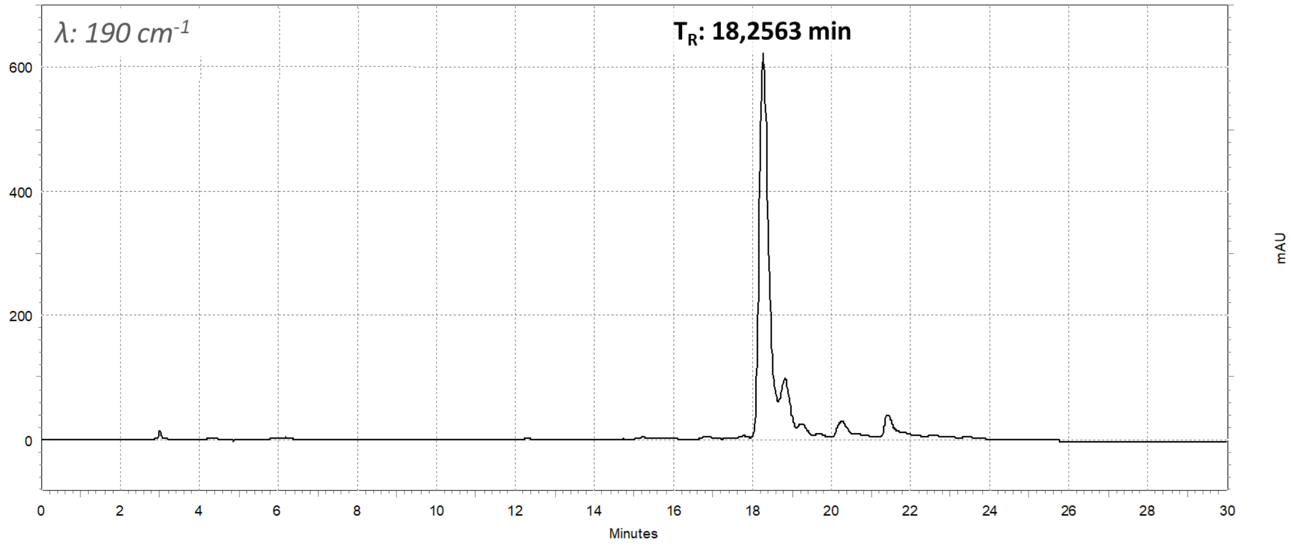

Figure S32. Chromatogram of **3** after purification by HPLC (RT = 18.26 min).

This is an open-access article distributed under the terms of the Creative Commons Attribution Licence.

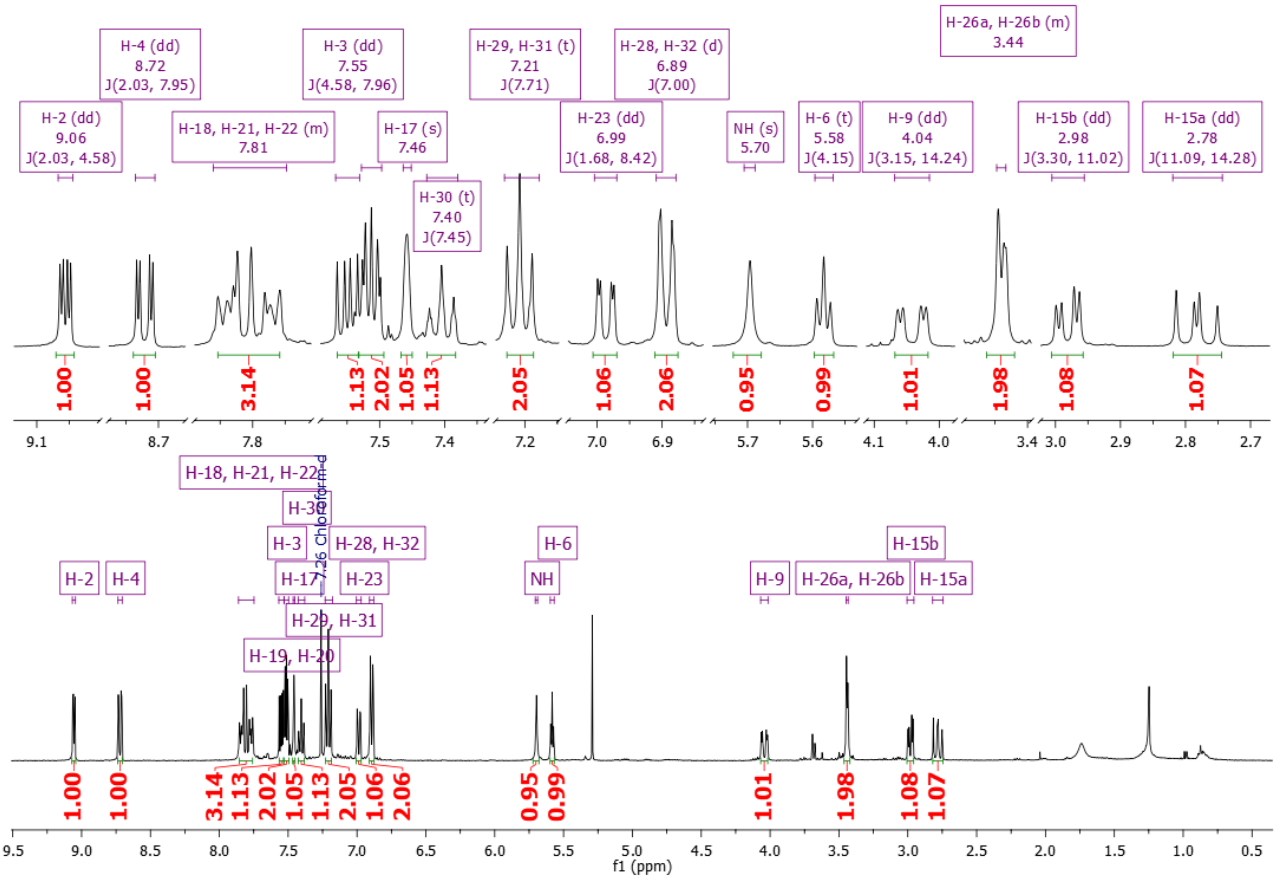

Figure S33. ^{1}H NMR spectrum (400 MHz, $CDCl_3$) of **4**.

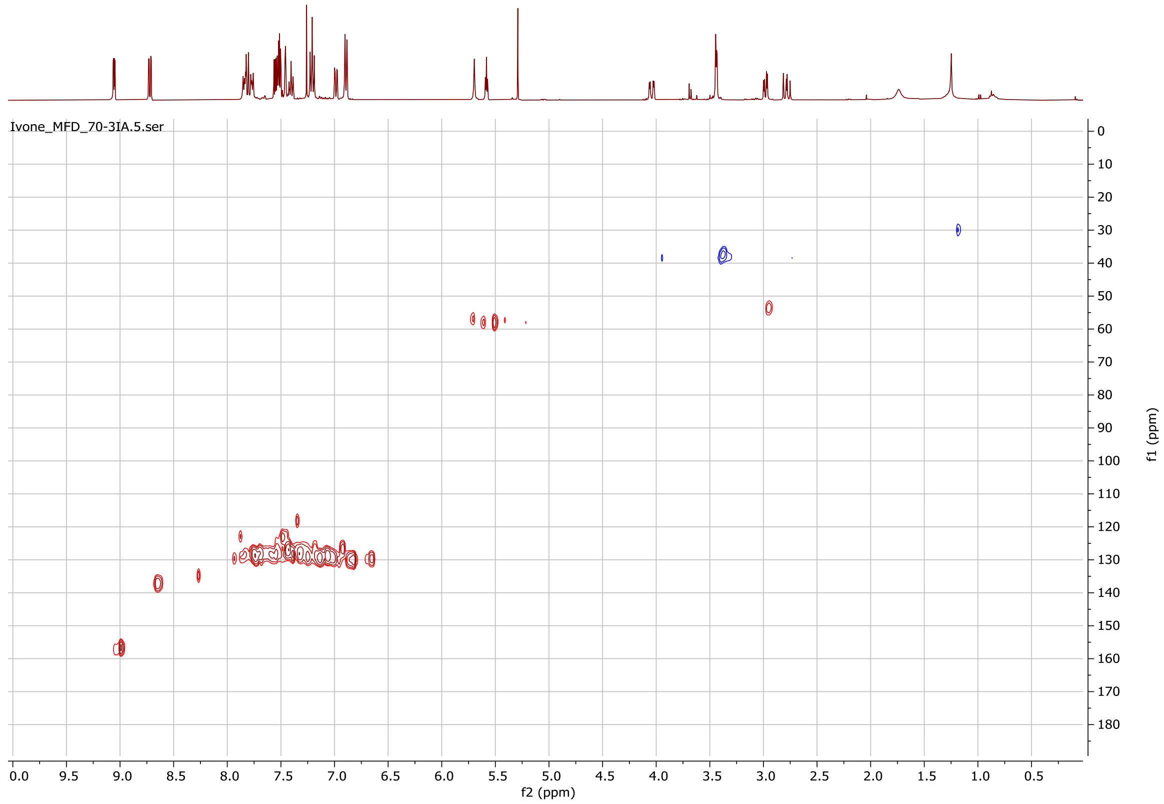

Figure S34. *g*GMQC NMR spectrum (150 MHz, $CDCl_3$) of **4**.

This is an open-access article distributed under the terms of the Creative Commons Attribution Licence.

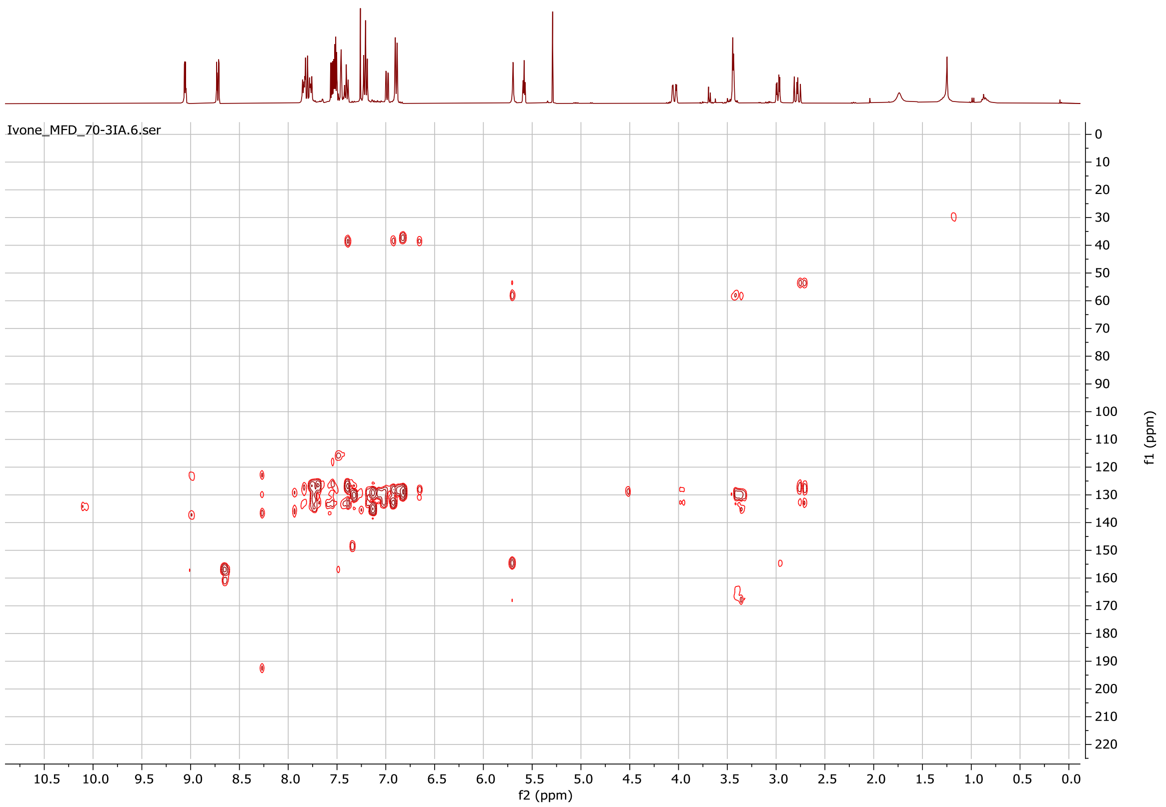

Figure S35. *g*GMBC spectrum (150 MHz, $CDCl_3$) of **4**.

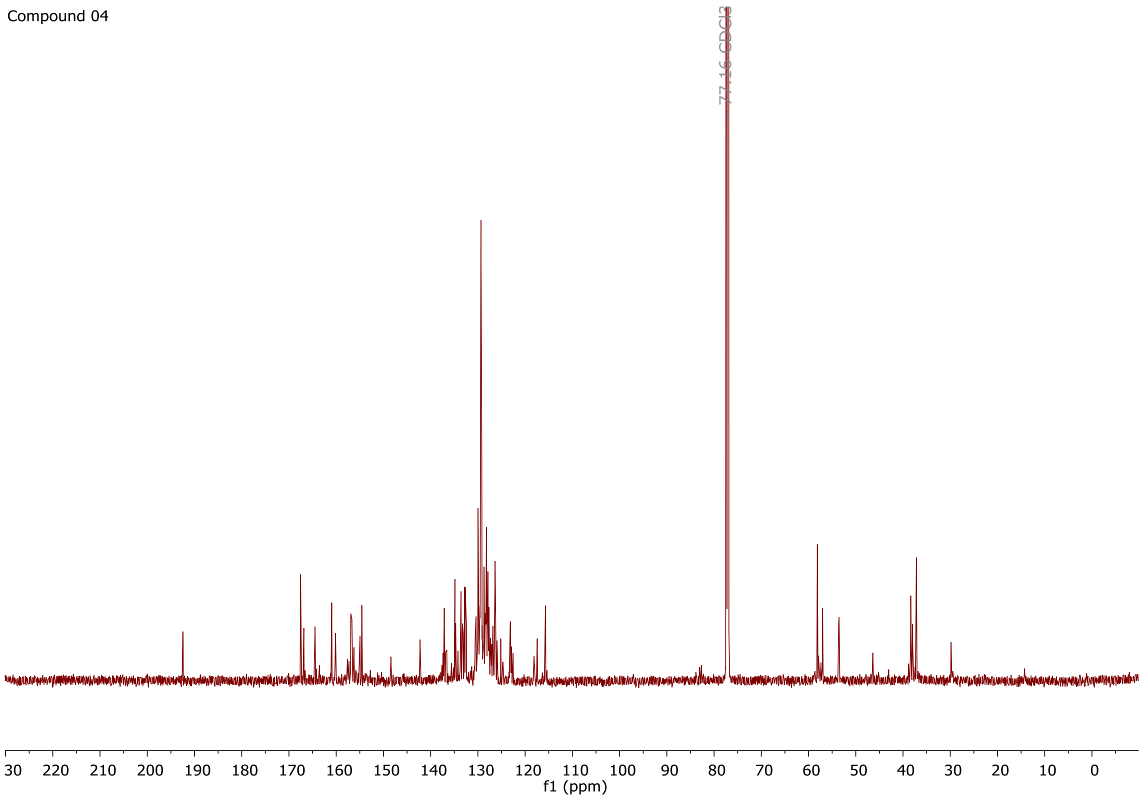

Figure S36. ^{13}C NMR spectrum (150 MHz, $CDCl_3$) of **4**.

This is an open-access article distributed under the terms of the Creative Commons Attribution Licence.

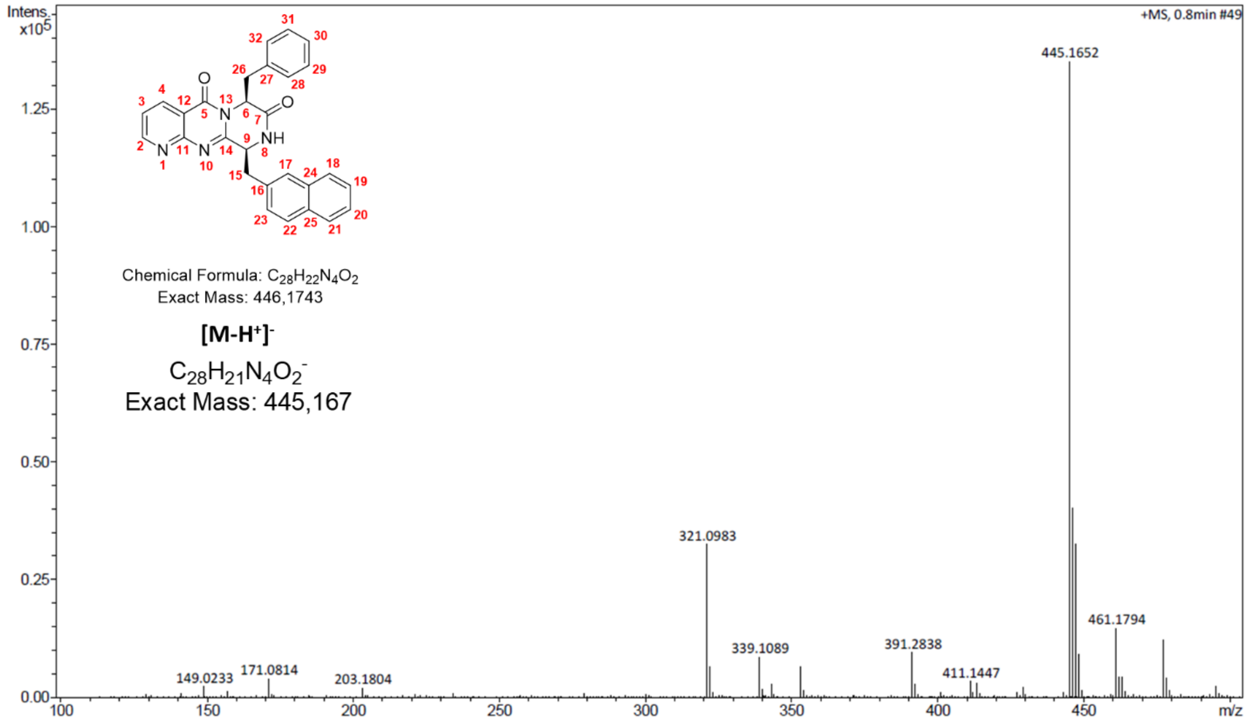

Figure S37. HRESI-MS spectrum of **4** (m/z calcd. for $C_{28}H_{21}N_4O_2^-$ $[M-H]^+$ 445.1670, found 445.1652).

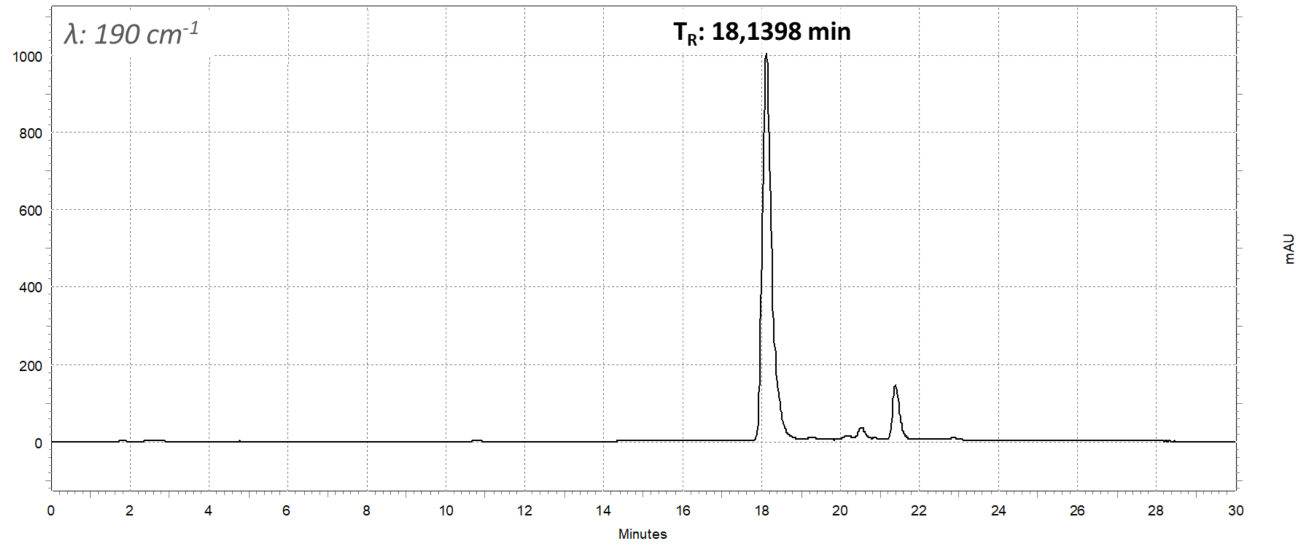

Figure S38. Chromatogram of **4** after purification by HPLC (RT = 18.14 min).

This is an open-access article distributed under the terms of the Creative Commons Attribution Licence.

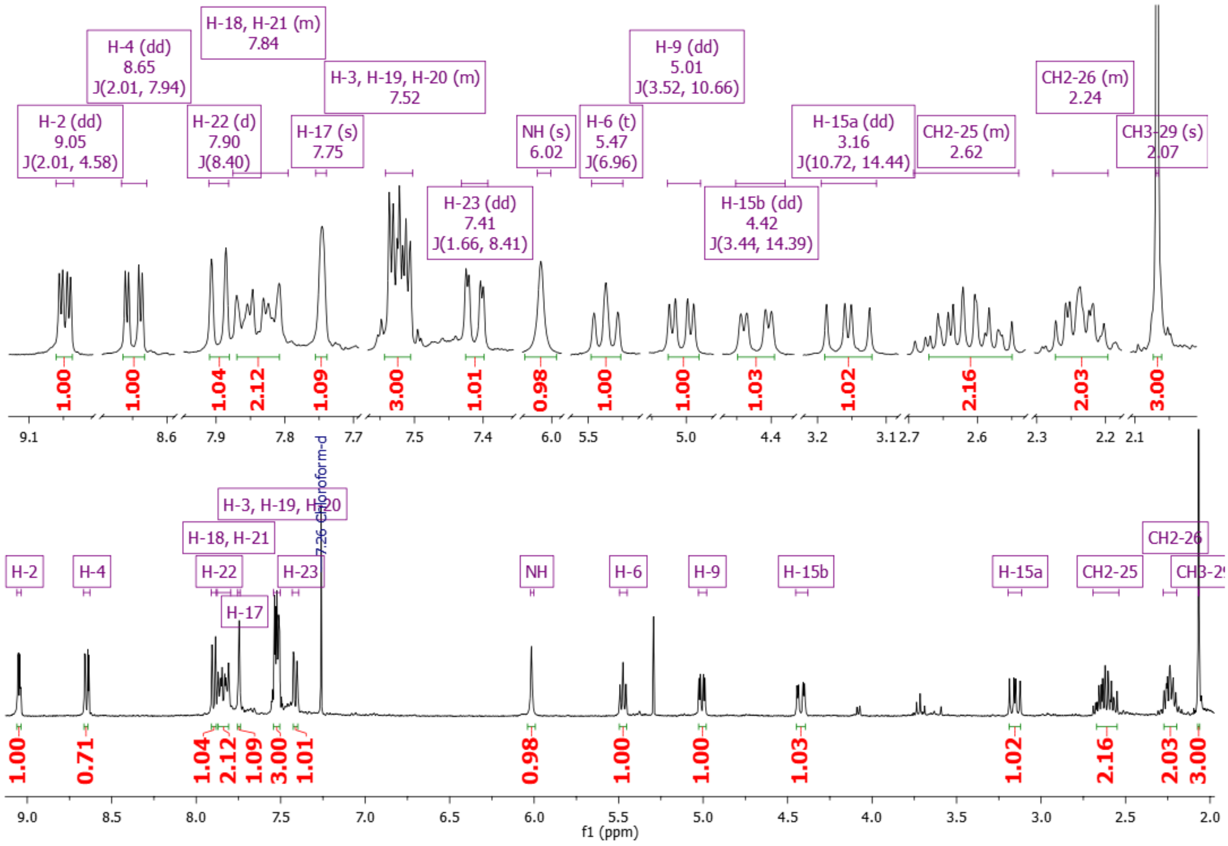

Figure S39. ^{1}H NMR spectrum (400 MHz, $CDCl_3$) of **7**.

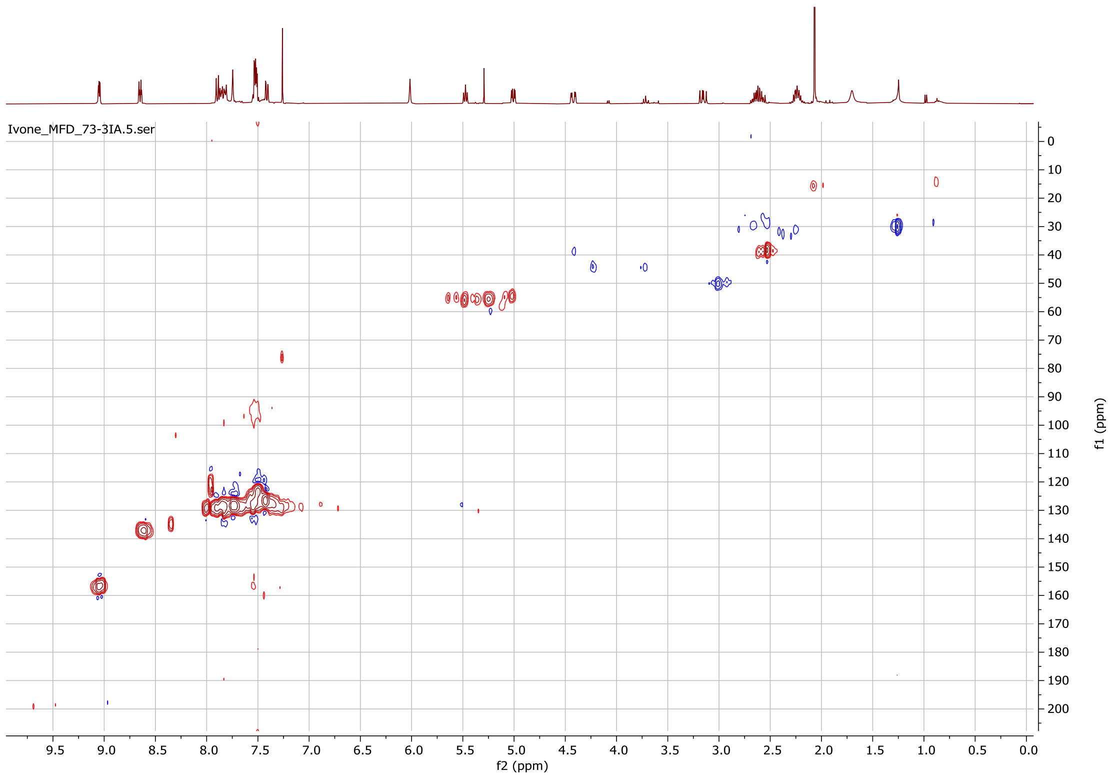

Figure S40. *g*GMQC NMR spectrum (150 MHz, $CDCl_3$) of **7**.

This is an open-access article distributed under the terms of the Creative Commons Attribution Licence.

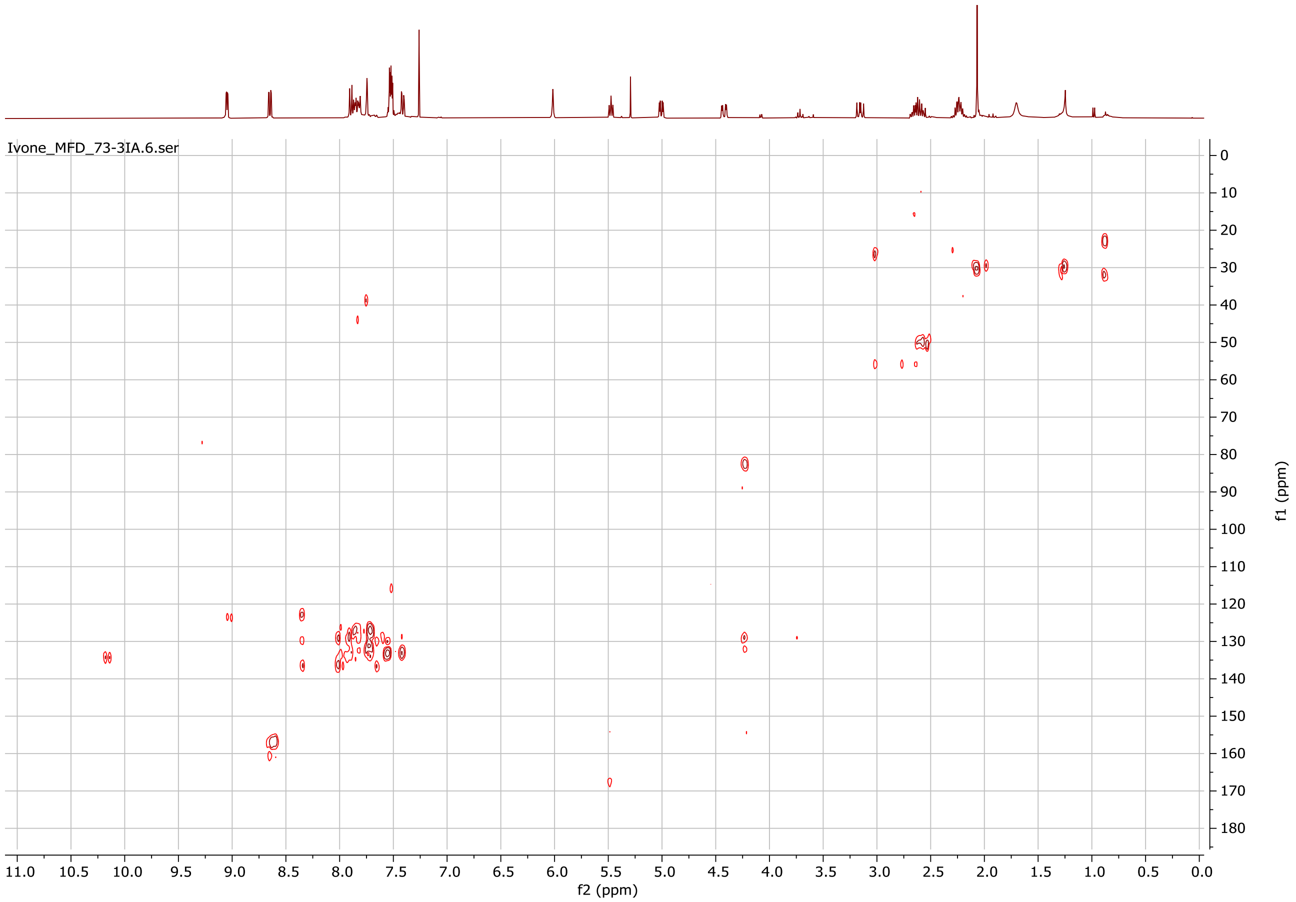

Figure S41. *g*GMBC spectrum (150 MHz, $CDCl_3$) of **7**.

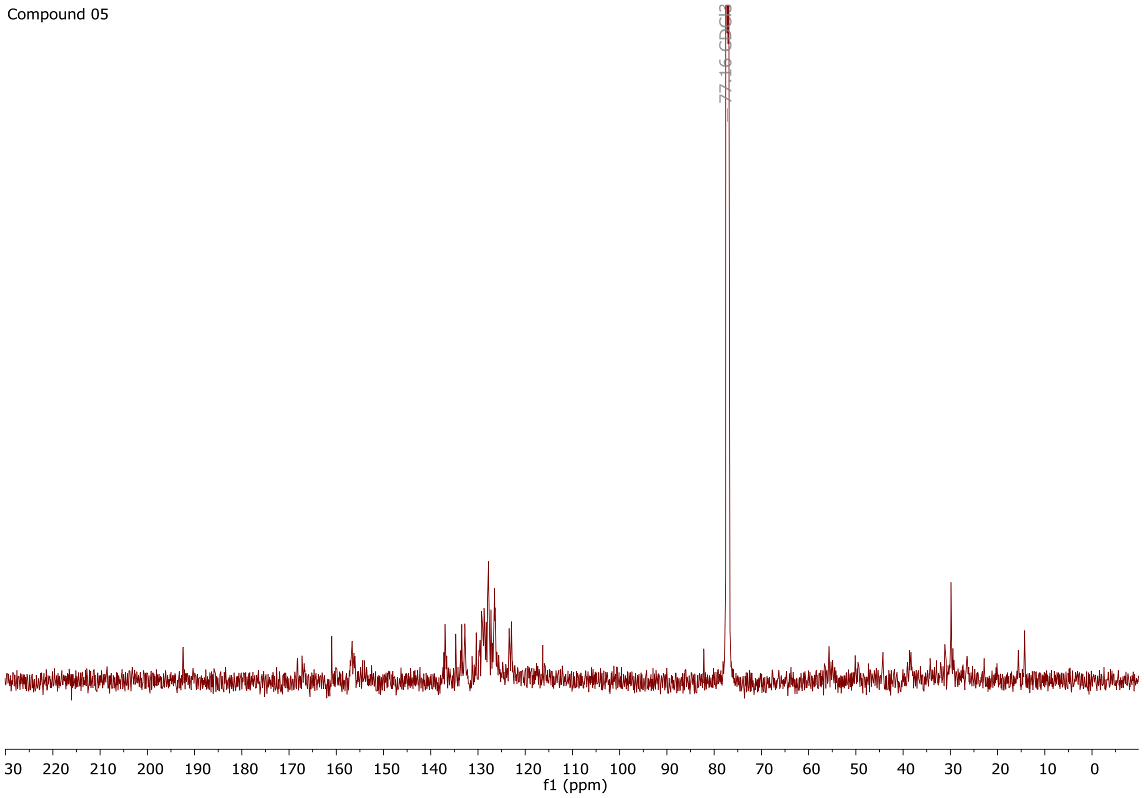

Figure S42. ^{13}C NMR spectrum (150 MHz, $CDCl_3$) of **7**.

This is an open-access article distributed under the terms of the Creative Commons Attribution Licence.

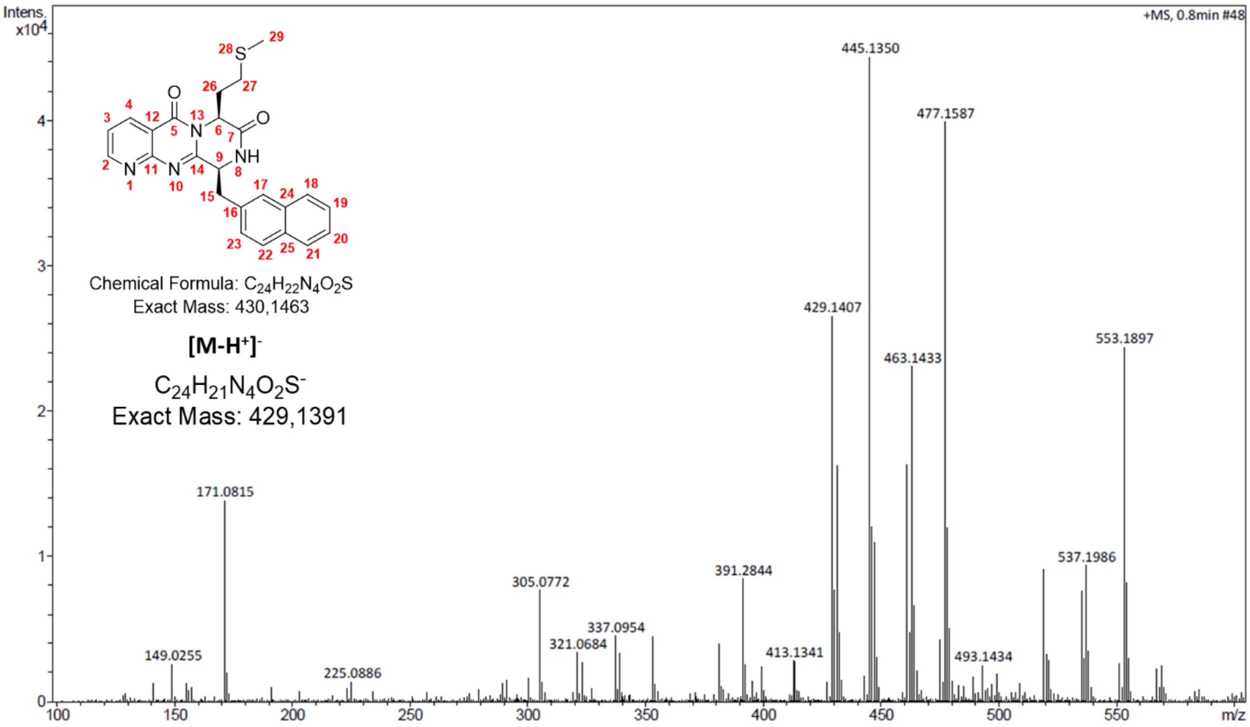

Figure S43. HRESI-MS spectrum of **7** (m/z calcd. $C_{24}H_{21}N_4O_2S^-$ $[M-H]^+$ 429.1391, found 429.1407).

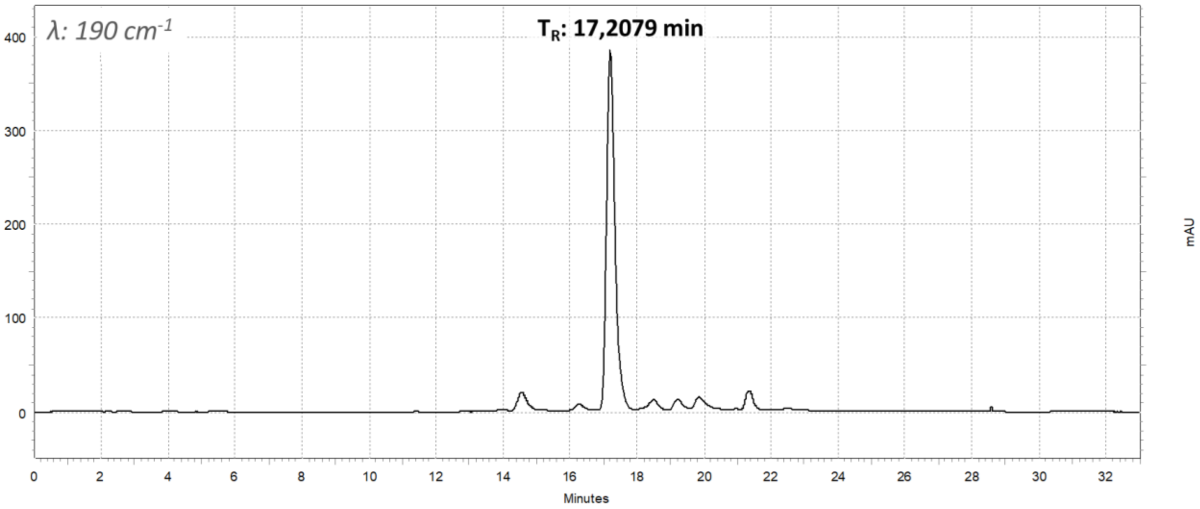

Figure S44. Chromatogram of **7** after purification by HPLC (RT = 17.21 min).

This is an open-access article distributed under the terms of the Creative Commons Attribution Licence.

HGC-27

	Controls (1 μM)		Series A (50 μM)												
	**Rapamycin**	**Gedatosilib**	**C1**	**C2 A**	**C3 A**	**C4 A**	**C5 A**	**C6 A**	**C7 A**	**C2 B**	**C3 B**	**C4 B**	**C5 B**	**C6 B**	**C7 B**
**EXP 1**	57,7262	71,2880	-2,0773	-7,4678	-0,1574	-2,0147	2,5440	2,3873	4,2102	-1,4246	0,4149	5,1287	6,6408	5,9057	7,7134
**EXP 2**	57,3579	70,7842	-6,7662	-11,2944	-4,8581	-8,2895	-0,1758	-1,7711	-1,5887	-8,4121	-5,8528	-1,7826	3,4284	-1,1502	1,5975
**EXP 3**	58,0945	71,7917	2,6115	-3,6412	4,5433	4,2602	5,2637	6,5457	10,0091	5,5629	6,6825	12,0401	9,8531	12,9616	13,8293
**AVERAGE**	**57,7262**	**71,2880**	**-2,0773**	**-7,4678**	**-0,1574**	**-2,0147**	**2,5440**	**2,3873**	**4,2102**	**-1,4246**	**0,4149**	**5,1287**	**6,6408**	**5,9057**	**7,7134**
**STANDARD DEVIATION**	**0,3683**	**0,5038**	**4,6888**	**3,8266**	**4,7007**	**6,2748**	**2,7198**	**4,1584**	**5,7989**	**6,9875**	**6,2676**	**6,9113**	**3,2123**	**7,0559**	**6,1159**

BT20

	Controls (1 μM)		Series B (50 μM)												
	**Rapamycin**	**Gedatosilib**	**C1**	**C2 A**	**C3 A**	**C4 A**	**C5 A**	**C6 A**	**C7 A**	**C2 B**	**C3 B**	**C4 B**	**C5 B**	**C6 B**	**C7 B**
**EXP 1**	59,7159	65,8118	5,6000	-0,9804	-5,5022	-5,8787	2,9241	-4,9474	1,4216	-3,0451	-0,7318	-4,7559	3,2155	-3,7205	1,0763
**EXP 2**	62,4917	69,5286	4,8014	0,2828	-3,8033	4,2694	-2,2739	1,8445	4,3598	-0,4344	1,9336	16,6551	0,9632	6,8239	2,8212
**EXP 3**	56,9402	62,0950	-6,7622	-11,2872	-7,9540	1,5788	-7,6208	0,9986	-10,4499	-1,0292	-11,4453	-10,2241	-8,4042	-4,6712	-1,4092
**AVERAGE**	**59,7159**	**65,8118**	**1,2131**	**-3,9949**	**-5,7532**	**-0,0101**	**-2,3235**	**-0,7014**	**-1,5562**	**-1,5029**	**-3,4145**	**0,5584**	**-1,4085**	**-0,5226**	**0,8294**
**STANDARD DEVIATION**	**2,7758**	**3,7168**	**6,9183**	**6,3468**	**2,0867**	**5,2573**	**5,2726**	**3,7014**	**7,8410**	**1,3683**	**7,0814**	**14,2058**	**6,1622**	**6,3800**	**2,1260**

Figure S45. Results from cell viability (inhibition) assays in HGC-27 and BT20 tumor cell lines following 72-hour exposure to series A and B derivatives (50 μM)

This is an open-access article distributed under the terms of the Creative Commons Attribution Licence.

CAL-27

Series C (50 µM)

	1	2	3	4	7
Exp 1	5,20400	9,41935	93,67593	94,06974	5,89114
Exp 2	4,80090	9,03418	93,64904	94,04453	5,49097
Exp 3	3,91268	8,18545	93,58979	93,98896	4,60918
AVERAGE	4,63919	8,87966	93,63825	94,03441	5,33043
Standard Deviation	0,66067	0,63130	0,04408	0,04133	0,65589

HGC-27

Series C(50 µM)

	1	2	3	4	7
Exp 1	10,60476	24,88577	84,70201	86,34367	16,85475
Exp 2	11,37879	25,53615	84,83447	86,46191	17,57467
Exp 3	13,20531	27,07088	85,14704	86,74094	19,27348
AVERAGE	11,72962	25,83093	84,89451	86,51551	17,90097
Standard Deviation	1,33530	1,12198	0,22851	0,20399	1,24194

BT20

Series C (50 µM)

	1	2	3	4	7
Exp 1	10,16817	29,44905	88,40786	89,48599	18,09370
Exp 2	8,79786	26,21314	87,87617	89,00375	14,33695
Exp 3	7,92411	27,68664	88,11828	89,22335	16,04763
AVERAGE	8,96338	27,78294	88,13411	89,23770	16,15943
Standard Deviation	1,13115	1,62011	0,26620	0,24144	1,88087

Figure S46. Results from cell viability (inhibition) assays in HGC-27, BT20 and BT20 tumor cell lines following 72-hour exposure to series C derivatives (50 µM)

BT20

Cell cycle phase (%)

		G1	G2	S
Control	Exp 1	30,80	19,40	49,90
	Exp 2	30,70	20,90	48,40
	Average	**30,80**	**20,10**	**48,60**
Gedatolisib (2.5 µM)	Exp 1	49,20	50,80	0,00
	Exp 2	48,80	51,00	0,19
	Average	**49,00**	**50,90**	**0,10**
**3** (20 µM)	Exp 1	53,60	11,30	35,10
	Exp 2	54,00	11,90	31,10
	Average	**53,80**	**11,60**	**33,10**
**4** (20 µM)	Exp 1	59,20	11,00	29,80
	Exp 2	60,00	11,60	28,20
	Average	**59,60**	**11,30**	**29,00**

CAL27

Cell cycle phase (%)

		G1	G2	S
Control	Exp 1	49,80	11,80	38,40
	Exp 2	50,10	11,50	38,50
	Average	**50,00**	**11,70**	**38,50**
Gedatolisib (2.5 µM)	Exp 1	49,90	50,00	0,10
	Exp 2	51,30	48,70	0,00
	Average	**50,60**	**49,40**	**0,10**
**3** (20 µM)	Exp 1	63,30	4,50	32,20
	Exp 2	64,10	5,00	30,90
	Average	**63,70**	**4,80**	**31,60**
**4** (20 µM)	Exp 1	75,40	2,50	22,10
	Exp 2	76,60	1,30	22,00
	Average	**76,00**	**1,90**	**22,10**

HGC27

Cell cycle phase (%)

		G1	G2	S
Control	Exp 1	35,00	7,20	57,80
	Exp 2	35,40	8,90	55,80
	Average	**35,20**	**8,05**	**56,80**
Gedatolisib (2.5 µM)	Exp 1	53,90	46,10	0,00
	Exp 2	51,30	48,60	0,10
	Average	**52,60**	**47,30**	**0,10**
**3** (20 µM)	Exp 1	63,90	8,50	27,60
	Exp 2	64,20	8,30	27,50
	Average	**64,10**	**8,40**	**27,52**
**4** (20 µM)	Exp 1	71,10	9,50	19,40
	Exp 2	71,00	10,20	18,80
	Average	**71,05**	**9,90**	**19,10**

Figure S47. Cell cycle phases analysis from tumor cell lines (BT20, CAL-27 and HGC-27) after 24 hours-exposure with compounds **3** and **4** (20 µM) and with gedatolisib (2.5 µM).

This is an open-access article distributed under the terms of the Creative Commons Attribution Licence.
